# Coupling photocatalytic water oxidation with reductive transformations of organic molecules

**DOI:** 10.1038/s41467-022-33778-9

**Published:** 2022-10-19

**Authors:** Xinzhe Tian, Yinggang Guo, Wankai An, Yun-Lai Ren, Yuchen Qin, Caoyuan Niu, Xin Zheng

**Affiliations:** College of Science, Henan Agricultural University, Zhengzhou, Henan 450002 P.R. China

**Keywords:** Photocatalysis, Synthetic chemistry methodology

## Abstract

The utilization of readily available and non-toxic water by photocatalytic water splitting is highly attractive in green chemistry. Herein we report that light-induced oxidative half-reaction of water splitting is effectively coupled with reduction of organic compounds, which provides a light-induced avenue to use water as an electron donor to enable reductive transformations of organic substances. The present strategy allows various aryl bromides to undergo smoothly the reductive coupling with Pd/g-C_3_N_4_* as the photocatalyst, giving a pollutive reductant-free method for synthesizing biaryl skeletons. Moreover, the use of green visible-light energy endows this process with more advantages including mild conditions and good functional group tolerance. Although this method has some disadvantages such as a use of environmentally unfriendly 1,2-dioxane, an addition of Na_2_CO_3_ and so on, it can guide chemists to use water as a reducing agent to develop clean procedures for various organic reactions.

## Abstract (web summary)

While reductive coupling strategies in organic synthesis are crucial, most require additional sacrificial or toxic reagents. Here, authors demonstrate water as mild reducing agent in the photochemical reduction of organic compounds paired with photocatalytic water oxidation.

## Introduction

Reductive couplings of aryl halides are of great significance in modern organic synthesis because the resulting biaryl skeletons are widely found in dyes, natural products, pharmaceutical compounds, and optoelectronic molecules^1,2^. As a result, considerable efforts have been devoted to the development of various methods for the reductive couplings^2,3^, where alcohols, amines, formate salt, hydrazine hydrate, magnesium, and solvents were permitted to serve as the reductants^1–8^. Recently, the photocatalytic strategies become popular due to their advantages, including mild reaction conditions and the use of green solar energy. For example, chemists have used photoredox or semiconductor catalysts to enable homo-couplings of aryl halides in the presence of reductants, e.g. triethylamine, *N*,*N*-diisopropylethylamine and methanol^2,3,9–11^.

**Fig. 1:**
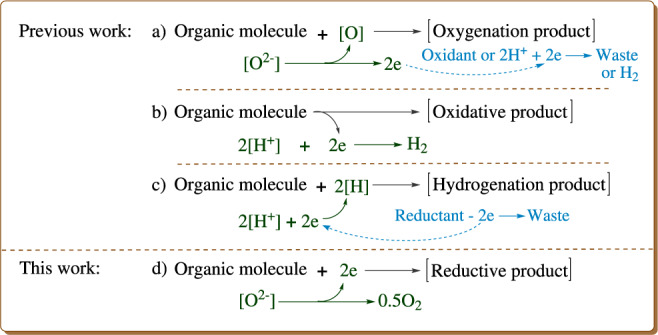
Background of this work. Organic reactions with H_2_O as the oxygen source (**a**), the oxidant (**b**), the hydrogen source (**c**) or the electron donor (**d**).

Compared with the above-mentioned reducing agents, water is more attractive in green chemistry due to its readily available, non-toxic, and non-flammable features^12,13^. Thus, much attention has been devoted to applications of water in various fields, e.g. organic reactions with water as the solvent^14–19^, hydrogen production by water splitting, and so on^20–28^. Recently, several uses of water in organic synthesis have been developed via the challenging photocatalytic water splitting. As shown in Fig. 1a, the first kind is the utilization of water as the oxygen source for the oxygenation of organic molecules by coupling the oxygenation with light-induced water oxidation half-reaction where oxidants such as [Co^III^(NH_3_)_5_Cl]^2+^ and (NH_4_)_2_Ce(NO_3_)_6_ have been added as the electron acceptor^29–32^. Subsequently, additional oxidant-free methods have been developed for the oxygenation of olefin and benzene C − H bonds by coupling the oxygenation with the two half-reactions of the water splitting (Fig. 1a)^31,32^. The second kind is the proton reduction half-reaction coupled with oxidation of organic compounds (Fig. 1b)^33–36^, and has provided a strategy for oxidative transformations of alcohols, thiols, and benzylamines^33–36^. The third kind is that the proton reduction is coupled with the hydrogenation (Fig. 1c)^37–40^, showing the use of water as the hydrogen source for the hydrogenation of olefins, nitro compounds, aldehydes, and halogenated compounds, in which reductants such as metal powders, triethanolamine, Na_2_SO_3_ and so on have been added to reduce H_2_O to hydrogen. Similar methods have also been applied in the deuteration of halogenated compounds with D_2_O^39,40^. To our knowledge, there is no report regarding coupling photocatalytic water oxidation half-reaction with the reduction of organic compounds (Fig. 1d) before submitting this manuscript. Therefore, we focused on overcoming this challenge to develop a method for using water as the reductant to enable the reductive coupling of aryl bromides.

On the other hand, graphite phase carbon nitride (g-C_3_N_4_)-based materials have been emerging as attractive photocatalysts for the hydrogen evolution^33–36,41,42^, the water oxidation^43,44^, oxidative degradation of pollutants, and the CO_2_ reduction^45,46^, due to the unique electronic band structure, high thermal and chemical stability of g-C_3_N_4_. In addition, this kind of materials has frequently served as photocatalysts for organic reactions, including reductive^27,47^, oxidative, and nonredox reactions of organic molecules^48–51^, encouraging us to select transition metal/g-C_3_N_4_ to make our idea come true.

In this work, we report that light-induced oxidative half-reaction of water splitting is effectively integrated with the reductive coupling of aryl bromides in the presence of the activated Pd/g-C_3_N_4_ (named as Pd/g-C_3_N_4_*) and Na_2_CO_3_, which provides a method for using green water as the reductant to enable the reductive coupling. Moreover, the use of visible-light as green energy endows this process with more advantages, including mild conditions, good functional group tolerance, and broad substrate applicability.

## Results

### Preparation and characterization of photocatalysts

Pd/g-C_3_N_4_ was prepared based on previous literatures^38,40^. In order to improve the catalytic activity, Pd/g-C_3_N_4_ was irradiated by blue light in the presence of Na_2_CO_3_ and H_2_O to give Pd/g-C_3_N_4_*. According to the TEM image (Supplementary Figs. 1 and 2), the Pd nanoparticles in Pd/g-C_3_N_4_* are uniformly dispersed and has a narrow size distribution in the range of 5–10 nm. In addition, there is a slight difference between the Pd nanoparticles size of Pd/g-C_3_N_4_* and that of Pd/g-C_3_N_4_ (Supplementary Fig. 2), suggesting that the light-irradiated treatment had a slight effect on the distribution of the Pd nanoparticles. As seen from X-ray diffraction patterns (Supplementary Fig. 3), the diffraction peaks from Pd had little change when Pd/g-C_3_N_4_ was activated by our method, which reveals that the activating treatment would not result in significant leaching losses of Pd nanoparticles.

**Fig. 2:**
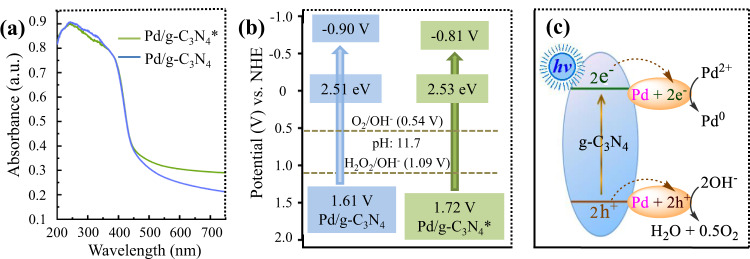
Effect of the light-irradiated treatment on Pd/g-C_3_N_4_. **a** UV-visible diffuse reflection spectra of Pd/g-C_3_N_4_ (blue curve, 2.8 wt%) and Pd/g-C_3_N_4_* (green curve, 2.8 wt%). a.u.: arbitrary units. Source data are provided as a Source Data file. **b** Energy-band positions of Pd/g-C_3_N_4_ (blue, 2.8 wt%) and Pd/g-C_3_N_4_* (green, 2.8 wt%). **c** Rationale for conversion of Pd^II^ to Pd^0^. Pd/g-C_3_N_4_*: the activated Pd/g-C_3_N_4_ by the light-irradiated treatment.

Next, the light capture capacity of the two catalysts was precisely examined by UV-visible diffuse reflection spectra. As shown in Fig. 2a, Pd/g-C_3_N_4_* displays a broader photoresponse performance that ranges from 200 to 750 nm, and the maximum absorption is centered at 200–380 nm. Compared with Pd/g-C_3_N_4_, Pd/g-C_3_N_4_* exhibits a slightly increase in adsorption at 450–750 nm wavelengths. The results from the calculation via the Tauc function show that the optical band gaps of Pd/g-C_3_N_4_ and Pd/g-C_3_N_4_* are 2.51 eV and 2.53 eV, respectively (Supplementary Fig. 4b). The conduction band (CB) positions vs. normal hydrogen electrode (NHE) were also clarified via electrochemical Mott-Schottky experiments (Supplementary Fig. 5), and the results show that the CB in two cases are −0.90 V and −0.81 V (Fig. 2b), respectively, ignoring the difference between the flat band gap and the CB. Thus the valence band (VB) positions in two cases can be estimated to be 1.61 V and 1.72 V, respectively (Fig. 2b), revealing that the electronic band structure of the catalyst had a slight change after our activating treatment.

We examined the chemical state of Pd by X-ray photoelectron spectroscopy (XPS). As shown in Supplementary Fig. 6, the deconvoluted peaks related to the 3*d*_3/2_ and 3*d*_5/2_ orbitals of Pd^0^ are 340.23 and 335.00 eV^52^, while another two shoulder peaks should be assigned to Pd^II^ in PdO^52^. Based on the area of the peaks, the percentages of Pd^0^ in the Pd nanoparticles are 54% for Pd/g-C_3_N_4_ and 73% for Pd/g-C_3_N_4_*, respectively (Supplementary Fig. 6), which indicates that our activating treatment can increase the percentage of Pd^0^. We examined the evolved gas and the results show that the molar ratio of the evolved O_2_, H_2_ and the increasing Pd^0^ is 0.5: 0.3: 0.7, which reveals that about 70% of electrons from the OER half-reaction (2OH^−^ → 0.5O_2_ + H_2_O + 2e^−^) enable the formation of Pd^0^ (Pd^II^ + 2e^−^ → Pd^0^), suggesting the OER half-reaction serves as the sacrificial electron donor for conversion of Pd^II^ to Pd^0^. Thus a rough mechanism for the Pd^0^ formation is presented in Fig. 2c based on the OER mechanism (see below) and the observation above.

### Effect of various conditions on the reductive coupling

**Fig. 3:**
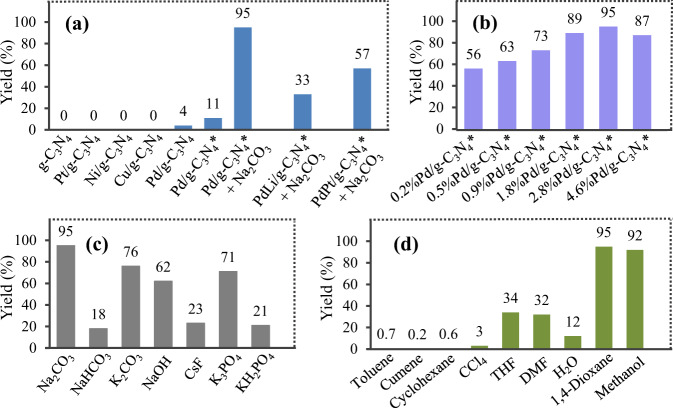
Effect of different conditions on the coupling of bromobenzene. **a** Effect of different photocatalyst systems. The loading of the metal on g-C_3_N_4_ is 2.8 wt% in all cases. M/g-C_3_N_4_: graphite phase carbon nitride-supported metal, M/g-C_3_N_4_*: the activated Pd/g-C_3_N_4_ by the light-irradiated treatment. **b** Effect of different Pd loading amounts. x%Pd/g-C_3_N_4_* means that the loading of Pd on g-C_3_N_4_ is x wt%. **c** Effect of different bases. Yield: yield of biphenyl. **d** Effect of different solvents. THF: tetrahydrofuran, DMF: *N*,*N*-dimethylformamide. For experimental procedures and conditions, see Unit 1.4.2, 1.4.3, and 1.4.4 in the supplementary information. Source data are provided as a Source Data file.

As shown in Fig. 3a, the reductive coupling didn’t occur in the case of bare g-C_3_N_4_, while the deposition of 2.8 wt% Pd on g-C_3_N_4_ gave the biphenyl product in 4% yield, suggesting that the Pd species served as the catalytic sites. Other transition metals, including Pt, Ni, and Cu supported on g-C_3_N_4_ were also test, but no targeted product was observed. Subsequently, we found that the yield increased significantly when Pd/g-C_3_N_4_ was irradiated with blue light prior to the addition of substrate, which indicates that the irradiating process can slightly enhance the catalytic ability of Pd/g-C_3_N_4_. To our delight, an addition of Na_2_CO_3_ resulted in a remarkable increase in the biphenyl yield. PdLi/g-C_3_N_4_^*^ and PdPt/g-C_3_N_4_^*^ were also examined as the catalysts, but the targeted product was obtained in only 33% and 57% yields, respectively. Subsequently, effect of the Pd loading on the reaction was investigated, and the results in Fig. 3b show that 2.8 wt% Pd loading is optimum. The yield significantly increased with increasing the Pd loading to 2.8 wt%. However, when the Pd loading increased from 2.8 to 4.6 wt%, the yield dropped, which is possibly rationalized by assuming that aggregation of excess Pd nanoparticles would lead to charge recombination^38^.

Among the screened bases, Na_2_CO_3_ was the most effective (Fig. 3c). Low yields were obtained in the case of using more weakly alkaline, including NaHCO_3_ and KH_2_PO_4_, while Na_2_CO_3_, K_2_CO_3_, K_3_PO_4_, and NaOH allowed the reaction to proceed with 62–95% yields, which indicates that the basicity has an important effect on the reaction. The reaction was highly dependent on the solvent type (Fig. 3d). The solvents, including toluene, cumene, cyclohexane, and CCl_4_ were less effective, possibly due to the poor dispersion of the catalyst in these solvents. On the contrary, the catalyst could be effectively dispersed in 1,4-dioxane and methanol, which allowed the coupling to proceed smoothly.

**Fig. 4:**
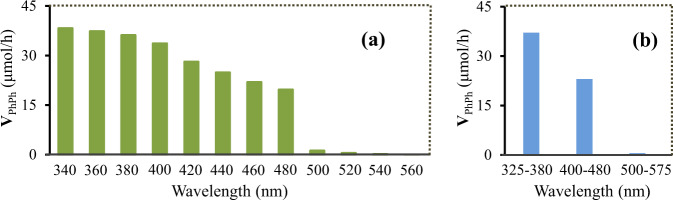
Coupling of bromobenzene under different wavelengths of light. **a** Effect of different monochromatic lights on the produced rate of biphenyl. x nm wavelength represents x ± 10 nm wavelength. **b** Effect of different polychromatic lights on the produced rate of biphenyl. V_PhPh_ represents the produced rate of biphenyl. For experimental procedures and conditions, see Unit 1.4.5 in the supplementary information. Source data are provided as a Source Data file.

Effect of the wavelength variation on the reaction was also investigated. As seen from Fig. 4a and Supplementary Figs. 9–11, both the rate of the biphenyl production and the apparent quantum efficiencies decreased with increasing the wavelength in the range from 340 ± 10 nm to 480 ± 10 nm, which is consistent with the variation tendency regarding the photoabsorption of the catalyst (Fig. 2a), revealing that the reaction rate is dependent on the photoresponse of the catalyst, and that the present coupling reaction is mainly triggered by the photoexcitation of the catalyst. As shown in Figs. 4a and 2a, when the wavelength exceeded the photoabsorption edge (490 nm) of Pd/g-C_3_N_4_* semiconductor, the coupling reaction was very sluggish in spite of an evident photoresponse of the catalyst (for the reasons behind these results, see Supplementary Note 1), which coincides with the assumption that the coupling reaction is mainly driven by the light-induced separation of electron-hole pairs in the semiconductor (for the detailed explanations, see Supplementary Note 1). Afterwards, we performed the reaction under irradiation of the light having a broad illumination spectrum. As seen from Fig. 4b, 325–380 nm polychromatic UV light allowed biphenyl to be produced in higher rate than that in the case of 400–480 nm polychromatic blue light, which is attributed to that the catalyst exhibits a stronger response to 325–380 nm UV light than to the blue light (Fig. 2a). The substrate was less reactive in the case of using more than 490 nm polychromatic light, which is consistent with the above performance of the monochromatic light.

### Substrates scope exploration for the reductive coupling

**Fig. 5:**
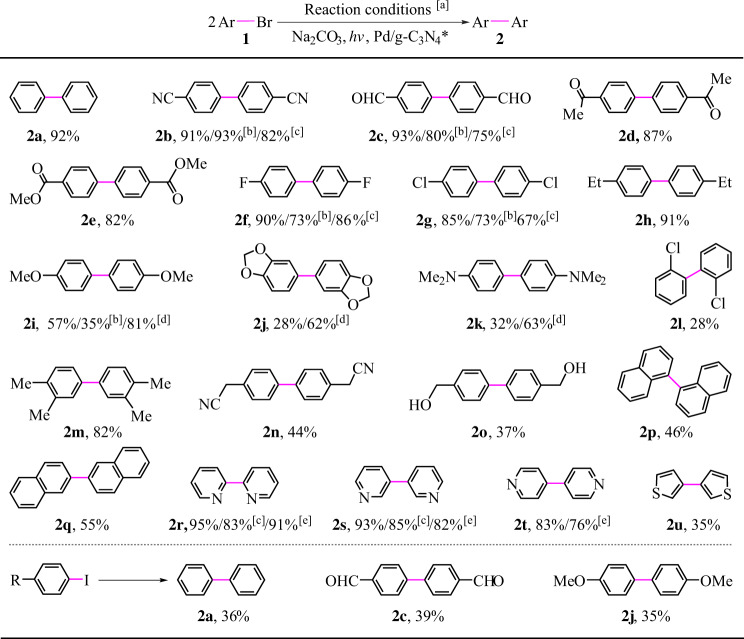
Reductive coupling of various aryl halides. **a** Standard conditions: 0.5 mmol aryl halides, 15 mg Pd/g-C_3_N_4_* (2.8 wt% Pd), 1.5 equiv Na_2_CO_3_, 5 mL H_2_O, 3 mL 1,4-dioxane, argon atmosphere, 20 h, room temperature (25 ^o^C), light source: 420 ± 10 nm LED (incident light intensity: 0.15 W/cm^2^), all the data shown in this figure are the isolated yields unless otherwise specified; **b** 10 h, GC yield; **c** incident light intensity: 0.10 W/cm^2^, GC yield; **d** 365 ± 10 nm LED; **e** 3 h, GC yield.

We set out to evaluate the scope and generality of the present method under the condition of 420 ± 10 nm blue light (Fig. 5). A series of bromobenzenes underwent the coupling smoothly to give the targeted products in low to high yields (**2a**–**2o**). Moreover, the present method was compatible with various groups, e.g. cyano, carbonyl, ester, fluoro, chloro, alkyl, alkoxy, *N*,*N*-dimethylamino, and hydroxy groups (**2b**–**2o**), even the highly reactive aldehyde group was also tolerated (**2c**). In most cases, the major byproducts were from debromination/hydrogenation of aryl bromides. Many bromobenzenes with electron-withdrawing groups (EWG) were converted to the targeted products in high yields (**2b**–**2** **g**), whereas the same conditions didn’t allow the substrates with strongly electron-donating groups (EDG) to be converted to the targeted products (**2i**–**2k**) in high yields. In addition, some of EWG-substituted bromobenzenes underwent the transformation to provide moderate to high yields of coupling products in the case of reducing the reaction time to 10 h or decreasing the incident light intensity to 0.10 W/cm^2^ (see **2b**, **2c**, **2** **f,** and **2** **g**). These results reveal that the presence of the EDG would decrease the substrate reactivity, which has been observed in literatures where homolytic cleavage of phenyl C-Br bonds begins with a single electron transfer (SeT) from the promoter to the substrate^53^.

**Fig. 6:**
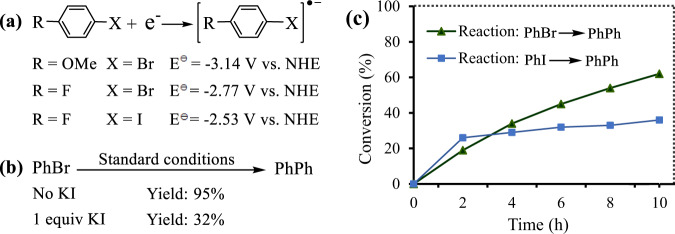
Effect of substituents or halogen ions. **a** Standard redox potential (E) of phenyl halides with different substituents. NHE represents a normal hydrogen electrode. **b** Effect of I^-^ on the reaction. For standard conditions, see Fig. 5. **c** Time course of the conversion of bromobenzene (green) and iodobenzene (blue). PhBr: bromobenzene, PhI: iodobenzene, PhPh represents biphenyl. Source data are provided as a Source Data file.

In theory, the negative effect of the EDG on the reactivity agrees with the SeT mechanism pathways proposed by us (see below) based on the following discussions: The EDG can increase the electron density of the bromobenzene moiety, which would suppress the SeT from the catalyst to the substrate by decreasing the electron-accepting capacity of the substrate. Indeed, Fig. 6a shows that bromobenzene with EDG has a lower redox potential than the substrate with EWG, suggesting that the SeT from the catalyst to the former is more difficult. Subsequently, we changed reaction conditions to improve the reactivity of the substrates containing EDG, and found that 365 ± 10 nm light allowed **2i-k** to be obtained in higher yields. As shown in Fig. 2a, the photoresponse of the catalyst becomes higher with decreasing the wavelength from 420 ± 10 to 365 ± 10 nm, which is obviously an important reason why the change in the wavelength increased the substrate reactivity.

The present reaction was susceptible to the steric hindrance. For example, the reaction of 4-bromochlorobenzene proceeded smoothly to provide **2** **g** in 85% yield, while only 28% yield was obtained in the case of 2-bromochlorobenzene (**1** **l**). We had tried the conversion of bromonaphthalenes, and the coupling of 1- and 2-bromonaphthalenes gave **2p** and **2q** in 46% and 55% yields, respectively. Another kind of good substrates was bromopyridines. Even reducing the reaction time to 3 h or decreasing the incident light intensity to 0.10 W/cm^2^ allowed the targeted products (**2r**, **2** **s**, and **2t**) to be obtained in satisfactory yields. By comparison, the electron-rich heteroaryl bromides, including bromofuranes and bromothiophenes were less reactive, suggesting that an increase in the electron density of the aromatic ring would decrease the substrate reactivity, agreeing with our observations above related to the electronic effect. Strangely, aryl iodides gave the targeted product in lower yields with lower conversions although we and other chemists provided evidence that aryl iodides are more reactive than aryl bromides (see Fig. 6a)^39,40,53^. We inferred that the resulting I^−^ from the coupling of iodobenzenes would block the present reaction by poisoning our photocatalyst. Indeed, when 1 equiv. KI was added to the reaction system, the yield of biphenyl decreased from 92% to 32% (Fig. 6b). This inference is in agreement with experimental results shown in Fig. 6c: The conversion rate of iodobenzene is higher than that of bromobenzene in 2 h, but the former becomes very slow after 2 h with the concentration of the produced I^−^ increasing. In addition, we examined conversion of various aryl bromides under irradiation of UV-light (350 ± 10 nm light or 325–380 nm polychromatic light) and blue light (400–480 nm polychromatic light). Compared with the blue light, the UV-light allowed all the substrates to be completely converted in shorter time (Supplementary Table 3), due to that the catalyst exhibits a stronger response to UV-light than to the blue light (Fig. 2a). It is worth noting that the aldehyde substituent is easily destroyed under irradiation of UV-light (see **2c** in Supplementary Table 3), owing to that the energy of UV-light photons is enough high to enable the homolysis of C-H bond in the aldehyde group in the absence of photocatalysts^54^.

As shown in Supplementary Tables 4 and 5, the most noteworthy characteristic of the present method is the use of green water as the reductant in the reductive couplings (entry 1 vs. entries 2–19)^1,5–9,55–60^. Moreover, the use of visible-light in our method endows this process with more advantages, including mild conditions and green energy (Supplementary Table 4, entry 1). Compared with other reductive aryl-aryl couplings (Supplementary Table 5, entries 2–19)^1,5–9,55–60^, the present reaction exhibited better functional group tolerance (Supplementary Table 5, entry 1). In addition, our method showed broader substrate applicability and allowed various phenyl, naphthyl, thienyl, and pyridinyl bromides to undergo the reductive coupling smoothly, whereas only phenyl and naphthyl bromides were tested in some of literatures^5–7,9,59,60^. Unfortunately, our method couldn’t be applied in the biaryl cross-coupling between different aryl halides, and inert aryl chlorides were less reactive under our conditions.

### Investigation on the recycling of Pd/g-C_3_N_4_*, the reproducibility of the catalyst and the necessity for using Pd/g-C_3_N_4_*, water, 1,4-dioxane, light, and heat

**Fig. 7:**
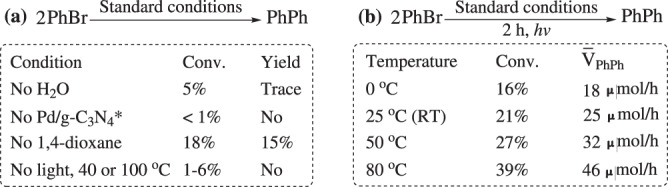
Several control experiments. **a** Investigation on the necessity for using water, Pd/g-C_3_N_4_*, 1,4-dioxane, and light. PhBr: bromobenzene, PhPh: biphenyl, Conv.: conversion of bromobenzene, Yield: yield of biphenyl. **b** Effect of temperature on the reaction. ${\bar{{{{{{\rm{V}}}}}}}}_{{{{{{\rm{P}}}}}}{{{{{\rm{h}}}}}}{{{{{\rm{P}}}}}}{{{{{\rm{h}}}}}}}$: the average rate of biphenyl production in 2 h. For standard conditions, see Fig. 5.

As shown in Supplementary Fig. 14, the catalyst could be recycled for three times with a very slight change in the catalytic activity (for the main reasons why the catalytic efficiency would decrease after four cycling runs, see Supplementary Note 2). When eight different batches of the catalyst were respectively used, the targeted product was obtained in high yields ranging from 92% to 96% (Supplementary Table 6), suggesting that the results regarding the catalytic activity of Pd/g-C_3_N_4_* are reliable and reproducible. The results from six parallel experiments regarding the coupling of bromobenzene also reveal that the reported results in the present paper are reliable and reproducible (Supplementary Table 7). As shown in Fig. 7a, hardly any coupling product was observed in the absence of water or Pd/g-C_3_N_4_*, indicating that both water and Pd/g-C_3_N_4_* were indispensable for the reaction. The absence of 1,4-dioxane would lead to poor dispersion of the catalyst in the solvent, thus the reaction didn’t go well in the pure water (Fig. 7a), which reveals that 1,4-dioxane served as the dispersant in the present reaction. We also performed the control experiments without irradiation, and the results show that the coupling wouldn’t occur under irradiation-free conditions (Fig. 7a), suggesting that the irradiation of light is indispensable for the present reaction. This conclusion was also supported by the following results: the coupling rate linearly increased with increasing the light intensity (Supplementary Fig. 20). This approximately linear relationship is often reported in literatures regarding g-C_3_N_4_ semiconductor-catalyzed organic reactions^61^, and indicates that the reaction is dominated by a single photon absorption event^61^. Similar to many photocatalytic reactions^45,46^, the coupling rate would increase with raising the reaction temperature (Fig. 7b).

### Investigation on who serves as the electron-donor in the reductive coupling

We inferred that water served as the electron donor by the oxidation of H_2_O to O_2_ under our conditions. Indeed, the formation of ^18^O_2_ was observed in the H_2_^18^O-labelling experiment where H_2_^18^O was added into the reaction system (Supplementary Fig. 21a). Maybe the resulting Br^-^ played the role of the electron donor via conversion of Br^-^ to Br_2_, but this possibility was ruled out based on our experimental results: No Br_2_ was detected after reaction (Supplementary Fig. 21c). In addition, it is possible that dioxane or the benzene ring served as the electron donor by the oxidation of them (Supplementary Fig. 21d). To rule out this possibility, we analysed the reaction system using GC-MS and HPLC-MS, but no detectable amount of products from the oxidation of dioxane or the benzene ring was observed. Obviously, the experimental results above confirm the reliability of our conclusion that only water serves as the electron donor in our reactions.

### Reason why the light illumination of Pd/g-C_3_N_4_ slightly improves the catalytic activity

**Fig. 8:**
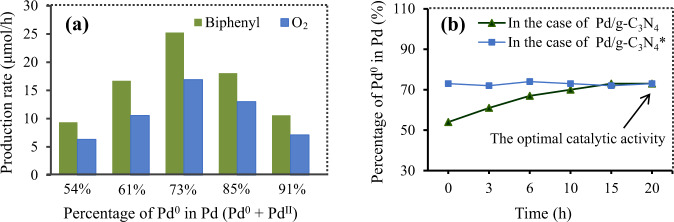
Relationship between the Pd^0^ concentration and the coupling. **a** Effect of the percentage of Pd^0^ on the average rate of the biphenyl (green) and O_2_ (blue) production in 2 h. **b** Time course of the Pd^0^ concentration in the case of Pd/g-C_3_N_4_ (green) or Pd/g-C_3_N_4_* (blue). For experimental procedures, see Unit 1.6 and 1.7 in the supplementary information. Source data are provided as a Source Data file.

As stated above, we irradiated Pd/g-C_3_N_4_ with blue light prior to the addition of aryl halides. Such a treatment would result in a conspicuous increase in the ratio of Pd^0^ to Pd^II^ (Supplementary Fig. 6), but didn’t lead to remarkable changes in the distribution of the Pd nanoparticles, the light capture capacity and the electronic band structure (see Fig. 2). In addition, when different batches of the catalyst were used, the yields of the coupling product fluctuated in a narrow range (Supplementary Table 6), suggesting that small changes in the distribution of the Pd nanoparticles wouldn’t be highly influential of the catalytic activity (Supplementary Note 3). These evidences reveal that the increase in the Pd^0^ concentration is the main reason why the catalytic activity is slightly improved. In theory, an increase in the percentage of Pd^0^ should be advantageous to the coupling half-reaction because Pd^0^ is the indispensable species for catalyzing this half-reaction^62,63^. However, with increasing the percentage of Pd^0^, the percentage of Pd^II^ would decrease, which should be disadvantageous to the water oxidation half-reaction because Pd^II^ serves as the catalytically active species for this half-reaction^64^. Thus both too-low and too-high percentages of Pd^0^ should be unfavorable to the overall reductive coupling reaction. Indeed, the production rate of biphenyl increases with increasing the percentage of Pd^0^ in Pd (Pd^0^ + Pd^II^) to the optimum value (73%) and then decreases (see Fig. 8a). We monitored the change of the Pd^0^ concentration throughout the reductive coupling (Fig. 8b), and the results reveal that the percentage of Pd^0^ in the case of Pd/g-C_3_N_4_ is still less than the optimum value (73%) regarding the catalytic activity in 10 h. It is also worth noting that increasing the Pd^0^ concentration would suppress the formation of benzene byproduct (Supplementary Table 8). Based on these observations, it can be concluded that the illuminating treatment of Pd/g-C_3_N_4_ prior to the addition of aryl halides makes the Pd^0^ concentration reach the optimum value in a short time (2-3 min), which not only slightly improves its catalytic activity, but also suppresses the formation of the benzene byproduct.

### Reason why Na_2_CO_3_ has a positive effect on the present reaction

**Fig. 9:**
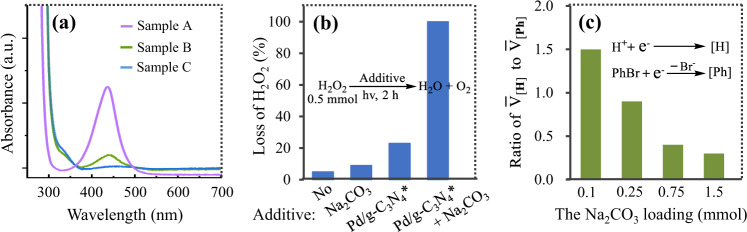
Investigation on the role of Na_2_CO_3_. **a** UV-Vis spectroscopy related to the produced H_2_O_2_ in the coupling of bromobenzene. Sample A (purple curve): 0.4 mmol/L aqueous solution of H_2_O_2_, Sample B (green curve): the produced H_2_O_2_ under standard conditions (no Na_2_CO_3_) in Fig. 5, Sample C (blue curve): the produced H_2_O_2_ under standard conditions in Fig. 5, a.u.: arbitrary units. **b** The H_2_O_2_ decomposition in the presence of additives. **c** Effect of the Na_2_CO_3_ loading on the ratio of ${{{{{{\rm{V}}}}}}}_{[{{{{{\rm{H}}}}}}]}$ to ${\bar{{{{{{\rm{V}}}}}}}}_{[{{{{{\rm{P}}}}}}{{{{{\rm{h}}}}}}]}$. PhBr: bromobenzene. [Ph]: intermediate regarding phenyl radical, [H]: intermediate regarding hydrogen radical. ${\bar{{{{{{\rm{V}}}}}}}}_{[{{{{{\rm{H}}}}}}]}$ and ${\bar{{{{{{\rm{V}}}}}}}}_{[{{{{{\rm{P}}}}}}{{{{{\rm{h}}}}}}]}$ represents the average rate of the [H] and [Ph] production in 4 h, respectively. Source data are provided as a Source Data file.

According to previous literatures^65,66^, the water oxidation often yields H_2_O_2_, and Na_2_CO_3_ can regenerate the photocatalysts by removing the resulting H_2_O_2_ that poisons metal/g-C_3_N_4_^65,66^. To verify this possibility, we analysed the reaction system using UV-Vis spectroscopy with *o*-tolidine as the indicator of H_2_O_2_. It is worth noting that the peak at 438 nm is a typical absorption peak that confirms H_2_O_2_^66^. As shown in Fig. 9a, H_2_O_2_ was observed for Na_2_CO_3_-free reaction system after Pd/g-C_3_N_4_*-catalyzed coupling was performed, while H_2_O_2_ disappeared in the reaction system in the presence of Na_2_CO_3_. These results suggest that the positive effect of Na_2_CO_3_ on the reaction is due to that it can remove the produced H_2_O_2_, or suppress the formation of H_2_O_2_ that poisons our photocatalyst. This conclusion is also verified by the evidences related to the formation mechanism of H_2_O_2_ in literatures^65,66^, and the following results (Fig. 9b): Both Na_2_CO_3_ and Pd/g-C_3_N_4_* were less effective to catalyze the H_2_O_2_ decomposition, whereas the simultaneous presence of Pd/g-C_3_N_4_* and Na_2_CO_3_ resulted in a complete decomposition of H_2_O_2_. As shown in Fig. 9c, the ratio of ${\bar{{{{{{\rm{V}}}}}}}}_{[{{{{{\rm{H}}}}}}]}$ to ${\bar{{{{{{\rm{V}}}}}}}}_{[{{{{{\rm{Ph}}}}}}]}$ becomes smaller and smaller with increasing the Na_2_CO_3_ concentration, suggesting that Na_2_CO_3_ can suppress the H^+^ reduction, which possibly attributes to that an addition of Na_2_CO_3_ decreases the H^+^ concentration in the reaction system. Thus it can be concluded that an inhibition effect of Na_2_CO_3_ on the undesired H^+^ reduction is also one of the main reasons why Na_2_CO_3_ can promote the reaction.

### Reaction mechanism for the reductive coupling

Two kinds of mechanisms for the reductive coupling have been proposed in the previous literatures. One undergoes the oxidative addition of aryl halides to the transition metals^1,3,11^. The other starts with the formation of the aryl radical anion (ArX**·**^**-**^) by the single electron transfer (SeT) from catalysts to aryl halides^3,39,40,67^. Considering that most of light-induced reductive couplings undergo the SeT mechanism pathways^3,39,40,67^, we guessed that the present reaction involved some radical-like species. Indeed, the presence of the radical inhibitor 2,2,6,6-tetramethyl-1-piperidinyloxy or 2,6-ditbutyl-4-methylphenol would prevent the targeted product from being produced (Supplementary Fig. 27a). According to previous literatures^1,2^, the oxidative addition of aryl halides to the Pd^0^ species can occur under thermal conditions, hence light-free conditions should allow the reductive coupling to proceed smoothly in the presence of stoichiometric Pd^0^ species as the electron-donor. On the contrary, when 1 equiv Pd^0^ species contained in the catalyst was used, the light-free coupling of bromobenzene didn’t occur at 100 ^o^C (Supplementary Fig. 27b), suggesting that the oxidative metal addition-based mechanism should be ruled out.

**Fig. 10:**
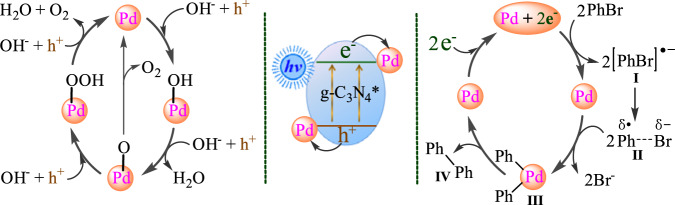
Proposed mechanism. Photocatalytic reductive coupling of bromobenzene using Pd/g-C_3_N_4_*. PhBr: bromobenzene, h^+^: hole, e^−^: electron.

Based on the observations above, a radical mechanism pathway is proposed with the coupling of bromobenzene as the representative. As shown in Fig. 10, g-C_3_N_4_ acts as the light absorber^41,42^, and its electrons are excited from the valence band (VB) to the conduction band (CB) upon irradiation with light. On the one hand, electrons of the CB of g-C_3_N_4_ transfer to Pd^0^ atoms that are just the active sites for the reductive coupling, which has been confirmed in many literatures^62,63^. Then intermediate **I** is produced via the electron transfer under assistance of Pd nanoparticles^1,2,11^, followed by the production of phenyl radical-like species **II**^1,2,11^. Next, the reaction between Pd and two molecules of species **II** provides diphenylpalladium **III**^1,2,11^. Finally, intermediate **III** undergoes reductive elimination to provide the targeted product^1,2,11^.

Subsequently, our attention was paid to clarifying the mechanism of the oxygen evolution (OER) half-reaction. Two kinds of pathways for the water oxidation to O_2_ have been proposed in the previous literatures^65,66^. One is the four-electron one-step process (Supplementary Fig. 28a)^68^, and the other proceeds via 2e^−^/2e^−^ two-step process (Supplementary Fig. 28b)^65,66^. According to the bandgap structure of Pd/g-C_3_N_4_* (see Fig. 2b), both the two processes are thermodynamically permitted. Thus we performed the rotating ring-disk electrode experiments to confirm the mechanism pathway. The electron-transfer number is 4 for the one-step process and 2 for the two-step process^65^, while our results show that the electron-transfer numbers range from 3.49 to 3.54 which are close to 4 (Supplementary Fig. 29), suggesting that the one-step process serves as the major pathway under our conditions. Thus a mechanism (see Fig. 10) is proposed based on the above observations and conventional mechanisms of electrochemical OER under alkaline conditions^68^.

## Discussion

In this work, we report that water is used as the electron-donor to enable the reductive transformations of organic molecules by coupling the light-induced water oxidation half-reaction with the reduction of organic compounds in the presence of Pd/g-C_3_N_4_* photocatalyst. The used photocatalyst is in-situ synthetized by a method where Pd/g-C_3_N_4_ is irradiated by the light in the presence of Na_2_CO_3_ and H_2_O. Such a treatment can slightly improve the activity of Pd/g-C_3_N_4_ by increasing the ratio of Pd^0^ to Pd^II^. In addition, our experimental results reveal that Na_2_CO_3_ has a considerably positive effect on the reaction by inhibiting the proton reduction and removing the produced H_2_O_2_ that poisons our photocatalyst. The present strategy allows various aryl bromides to undergo smoothly the reductive coupling under catalysis of Pd/g-C_3_N_4_*, providing a pollutive reductant-free method for synthesizing biaryl skeletons. Moreover, the use of visible-light as the green energy endows this process with more advantages including mild conditions, good functional group tolerance, and broad substrate applicability. Unfortunately, the present method has some disadvantages, such as the use of environmentally unfriendly 1,2-dioxane and an addition of Na_2_CO_3_. However, we believe that these results can guide chemists to use water as a reductant to develop clean procedures for various organic reactions by changing the composition of the semiconductor photocatalyst.

## Methods

### Procedure for preparation of Pd/g-C_3_N_4_

After 50 mg g-C_3_N_4_ were added to a 100 mL flask equipped with 50 mL ethanol, the system was sonicated for 3 h to make g-C_3_N_4_ to be dispersed in ethanol. Then 100 mL K_2_PdCl_6_ solution (0.01 M) was added, and the mixture was stirred for 10 min. Subsequently, 5 mL of water was added, and the mixture was refluxed at 90 ^o^C for 1 h. Finally, the reaction mixture was cooled to room temperature, the precipitation was collected, washed with ethanol, dried at 60 ^o^C under reduced pressure to give Pd/g-C_3_N_4_.

### Procedure for preparation of Pd/g-C_3_N_4_*

15.00 mg Pd/g-C_3_N_4_ and 79.50 mg Na_2_CO_3_ were added to a 10 mL quartz glass tube equipped with 5 mL H_2_O, 3 mL 1,4-dioxane, and a magnetic stirring under argon atmosphere. Then the reaction mixture was magnetically stirred for 2–3 min under the irradiation (light source: 420 ± 10 nm LED, incident light intensity: 0.15 W/cm^2^). Once the reaction time was reached, the precipitate was filtrated and washed in turn with water and ethanol. The collected solid was dried at 80 ^o^C under reduced pressure to give Pd/g-C_3_N_4_*.

### General procedure for the reductive coupling

15.0 mg Pd/g-C_3_N_4_ and 79.5 mg Na_2_CO_3_ were added to a 10 mL quartz glass tube equipped with 5 mL H_2_O and 3 mL 1,4-dioxane under argon atmosphere. After the reaction mixture was magnetically stirred for 2–3 min under the irradiation (light source: 420 ± 10 nm LED, incident light intensity: 0.15 W/cm^2^. Note: according to our measurement results, when the power of the light source was set as 75 W, the actual incident light intensity in the reaction tube was 0.15 W/cm^2^) to give in-situ Pd/g-C_3_N_4_*, 0.5 mmol aryl bromide was added. Then the reaction tube was sealed and placed in a constant-temperature bath (25 ^o^C) to perform the reductive coupling for 20 h under the irradiation (light source: 420 ± 10 nm LED, incident light intensity: 0.15 W/cm^2^) and argon atmosphere. Once the reaction time was reached, GC analysis of the mixture provided GC yields. The crude product from another parallel experiment was purified by silica gel chromatography to give the desired product.

### Characterization

The photocatalysts were characterized by transmission electron microscope (TEM), X-ray photoelectron spectroscopy (XPS), X-ray diffraction patterns, UV-Vis spectroscopy. The reductive coupling products were confirmed by ^1^H-NMR and ^13^C-NMR spectra. The details of these techniques and the other experimental procedure were shown in the supplementary information.

## Supplementary information

Supplementary Information

## Data Availability

The data that support the findings of this study are available within the paper and its supplementary information files. Extra data are available from the author upon request. Source data are provided with this paper.

## Source data

Source Data
