# Supplementary material for: Coupling photocatalytic water oxidation with reductive transformations of organic molecules
Source: Nat Commun. 2022 Oct 19;13:6186. doi: 10.1038/s41467-022-33778-9 (PMC9581948; doi:10.1038/s41467-022-33778-9)
Supplement: Supplementary file 1 — Supplementary Information [file 41467_2022_33778_MOESM1_ESM.pdf]

Supplementary Information

Coupling photocatalytic water oxidation with reductive transformations of organic molecules

Xinzhe Tian[1,2], Yinggang Guo[1,2], Wankai An[1], Yun-Lai Ren[1] ✉, Yuchen Qin[1], Caoyuan Niu[1], Xin Zheng[1]

[1]College of Science, Henan Agricultural University, Zhengzhou, Henan 450002, P.R. China. [2]These authors contributed equally: Xinzhe Tian, Yinggang Guo. ✉email: renyunlai@126.com

Table of Contents

1. Supplementary methods……………....….……………………………………...…………......1
1.1 General…………...………………...….……………………………………...…………......1
1.1.1 Chemicals………………………….................……………………………….…...........1
1.1.2 Instruments……………………………..………......................................….........1
1.2 Experimental procedure for preparation of the catalysts…...…..…………………….............1
1.2.1 Experimental procedure for preparation of g-C_3N_4...……………...……..………...........2
1.2.2 Experimental procedure for preparation of Pd/g-C_3N_4 (2.8 wt% Pd) ………...............2
1.3 Photoelectrochemical measurements……...……………………………...………….........2
1.4 Experimental procedure for the reductive coupling ……………………..………….........3
1.4.1 Representative experimental procedure for the reductive coupling……………..........3
1.4.2 Experimental procedure for the reductive coupling with various catalysts.................3
1.4.3 Experimental procedure for the reductive coupling with various bases......................4
1.4.4 Experimental procedure for the reductive coupling with various solvents 5
1.4.5 Experimental procedure for the reductive coupling at different wavelengths.............5
1.4.6 Experimental procedure for the reductive coupling of various aryl halides................5
1.5 Experimental procedure for obtaining the AQE………………………...…….………........6
1.6 Computational details for obtaining the standard redox potential………..……......……........7
1.7 Procedure for effect of I^- on the reaction and time course of yields of biphenyl.……............7
1.8 Experimental procedure for catalytic recycling test of Pd/g-C_3N_4*……..….…...……..........8
1.9 Procedure for investigating the reproducibility and the reliability of the results.……............8
1.10 Procedure for investigating the necessity for using Pd/g-C_3N_4*, water, 1,4-dioxane, light and heat………………………………………………………...…..…………...........9
1.11 Procedure for investigating who is the electron donor………………...…..……………........11
1.12 Experimental procedure for effect of the Pd^0 concentration on the reaction……….........11
1.13 Procedure for investigating the reasons why Na_2CO_3 has a positive effect on the coupling reaction……………………………………………..……………...…..……………........12
1.14 Procedure for investigating whether or not the coupling undergoes the SeT mechanism...14
1.15 Procedure for determining the electron transfer number…………...…..………………........14
2. Supplementary notes…………………………………..………..…...…..………………........14
2.1 Supplementary Note 1: Reasons why the coupling reaction was very sluggish in spite of an evident photoabsorption of the catalyst at more than 490 nm wavelengths………..…........14
2.2 Supplementary Note 2: Reasons why the catalytic efficiency would decrease after four cycling runs…………………………………..………...………..…...…..……………........16
2.3 Supplementary Note 3: Discussion regarding the reproducibility and the reliability of the results…………………………..………………………...………..…...…..……..………........17
3. NMR data regarding the coupling products…….………………..……..………..………........18
4. Supplementary tables…….…………………..………...…………...………..…...…..………........22
5. Supplementary figures….………………..………...……………...……..………..…..………........28
6. Copies of product NMR spectra………...………...……………...……..………..……………........38
7. Supplementary references…………..………...……..………...……..…...…..………………........55

1. Supplementary methods

1.1 General

1.1.1 Reaction chemicals

The quality and suppliers of the reagents are listed in Supplementary Table 1. Benzhydrol was synthesized by the reaction between the benzophenone and $LiAlH_4$. All the other chemicals were obtained from commercial vendors and used without further purification.

1.1.2 Instrumtents

^{1}H-NMR and ^{13}C-NMR spectra were recorded on a Bruker 500 MHz instrument with chemical shifts reported in ppm relative to the internal standard tetramethylsilane. GC-MS spectra was recorded on an Agilent 6890/5973N gas chromatography-mass spectrometry instrument. Gas chromatography analyses were performed on a Varian CP-3800 instrument with a FID detector and a CP-WAX 57CB FS capillary chromatographic column (25 m × 0.32 mm). The morphology of Pd/g-$C_3N_4^*$ was investigated by a FEI talos F200S transmission electron microscopy (TEM). X-ray photoelectron spectroscopy (XPS) analysis was performed on a ESCALAB 250Xi. The X-ray diffraction (XRD) analysis was performed on a SmartLab-9KW using Cu Kα radiation (λ = 1.5418 Å) working at 40 mA and 40 kV. The diffraction data were collected from 10 ° to 90° with a resolution of 0.01° (2θ). The UV-Vis absorption spectra were obtained on a JASCO model V-670 spectrometer equipped with an integrating sphere. Cyclic voltammetry curves were conducted on CHI760E Electrochemical Workstation (Shanghai Chen Hua Electrochemical Instrument) by using a three-electrode (a glassy-carbon or ITO working electrode, a saturated calomel reference electrode and a Ag/AgCl wire counter electrode) electrochemical cell. Photocatalytic coupling reactions were performed in a photochemical reactor (PLR MFPR-I, made by Beijing bofilai Technology Co., Ltd. Note: according to our measurement results, when the power of the used LED light source was set as 75 W and 50 W, the actual incident light intensity in the reaction tube was 0.15 W/cm^2 and 0.10 W/cm^2, respectively).

1.2 Experimental procedure for preparation of the catalysts

1.2.1 Procedure for preparation of g-C_3N_4

Polymeric carbon nitride (g-C_3N_4) was prepared based on previous procedures in literatures[1,2]. After 774.42 mg of cyanuric acid and 756.72 mg of melamine were added to a flask equipped with 45 mL of water, the system was shaken for 24 h to give a milky suspension. Subsequently, the obtained suspension was centrifuged, and then dried at 60 ^oC under vacuum. Then the obtained powder was annealed in a crucible at 550 ^oC for 4 h under Ar atmosphere, and then cooled to room temperature to provide yellow g-C_3N_4 powder.

1.2.2 Procedure for preparation of Pd/g-C_3N_4 (2.8 wt% Pd).

Pd/g-C_3N_4 was prepared based on previous procedures in literatures[1,2]. After 50 mg of g-C_3N_4 were added to a 100 mL flask equipped with 50 mL of ethanol, the system was sonicated for 3 h to make g-C_3N_4 to be dispersed in ethanol. Then 100 mL K_2PdCl_6 solution (0.01M) was added and the mixture was stirred for 10 min. Subsequently, 5 mL of water was added and the mixture was refluxed at 90 ^oC for 1 h. Finally, the reaction mixture was cooled to room temperature, the precipitation was collected, washed with ethanol, dried at 60 ^oC under reduced pressure to give Pd/g-C_3N_4 (2.8 wt% Pd). The Pd loading amount was determined by the inductively coupled plasma–mass spectrometry.

1.2.3 Procedure for preparation of Pd/g-C_3N_4* (2.8 wt% Pd). 15.00 mg of Pd/g-C_3N_4 and 79.50 mg of Na_2CO_3 were added to a 10 mL quartz glass tube equipped with 5 mL of H_2O, 3 mL of 1,4-dioxane and a magnetic stirring under argon atmosphere. Then the reaction mixture was magnetically stirred for 2-3 min under the irradiation (light source: 420±10 nm LED, incident light intensity: 0.15 W/cm^2). Once the reaction time was reached, the precipitate was filtrated and washed in turn with water and ethanol. The collected solid was dried at 80 ^oC under reduced pressure to give Pd/g-C_3N_4* (2.8 wt% Pd). The Pd loading amount was determined by the inductively coupled plasma–mass spectrometry.

1.3 Photoelectrochemical measurements

Photoelectrochemical measurements for Mott–Schottky plots and the photocurrent response were performed on three-electrode system using a CHI-660E electrochemical workstation (Chenhua, China). The platinum (Pt) foil and Ag/AgCl electrode (with saturated KCl solution) were used as counter electrode and reference

electrode, respectively. The working electrode was fabricated as follows: Pd/g-C_3N_4 sample (5 mg) was ultrasonically dispersed in 0.5 mL mixed solution (1 mL Nafion (5 wt%) and 9 mL ethanol) for 30 min to obtain the slurry. The slurry (100 μL) was then coated to the surface of FTO substrate (1 cm × 2 cm). After drying at 80 ℃ for 1 h, the working electrode was used for the following electrochemical tests in a Na_2SO_4 aqueous electrolyte solution (0.5 M). For photocurrent response tests, the light was provided by a LED lamp (10 W, 420 ± 10 nm).

1.4 Experimental procedure for the reductive coupling

1.4.1 Representative procedure for the reductive coupling

15.0 mg Pd/g-C_3N_4 and 79.5 mg Na_2CO_3 were added to a 10 mL quartz glass tube equipped with 5 mL H_2O and 3 mL 1,4-dioxane under argon atmosphere. After the reaction mixture was magnetically stirred for 2-3 min under the LED irradiation (note: according to our measurement results, when the power of the used LED light source was set as 75 W and 50 W, the actual incident light intensity in the reaction tube was 0.15 W/cm^2 and 0.10 W/cm^2, respectively) to give in-situ Pd/g-C_3N_4*, 0.5 mmol aryl bromide was added. Then the reaction tube was sealed and placed in a constant-temperature bath (25 ^{o}C) to perform the reductive coupling for 20 h under and argon atmosphere and the LED irradiation. Once the reaction time was reached, GC analysis of the mixture provided GC yields. The crude product from another parallel experiment was purified by silica gel chromatography to give the desired product.

1.4.2 Experimental procedure for the reductive coupling with various catalysts

Procedure for the first five experiments in Supplementary Fig. 7a: 0.5 mmol bromobenzene, 15 mg of M/g-C_3N_4 (2.8 wt% M) or g-C_3N_4 were added to a 20 mL quartz glass tube equipped with 5 mL of H_2O, 3 mL of 1,4-dioxane and a magnetic stirring under argon atmosphere. Then the reaction tube was sealed and placed in a constant-temperature bath (25 ^{o}C) to perform the reductive coupling for 20 h under blue light irradiation (light source: 420 ± 10 nm LED, incident light intensity: 0.15 W/cm^2) and argon atmosphere. Once the reaction time was reached, GC analysis provided the GC yields of the product with 1,2,3,4-tetramethylbenzene as an internal standard.

Procedure for the last four experiments in Supplementary Fig. 7a: 15.00 mg Pd/g-C_3N_4 (2.8 wt% Pd) or PdLi/g-C_3N_4 (2.8 wt% Pd, 1.9 wt% Li) or PdPt/g-C_3N_4 (2.8 wt% Pd, 2.7 wt% Pt), and 79.50 mg Na_2CO_3 (as shown in Fig. 7a, no Na_2CO_3 was added in the sixth experiment) were added to a 10 mL quartz glass tube equipped with 5 mL H_2O, 3 mL 1,4-dioxane and a magnetic stirring under argon atmosphere. After the reaction mixture was magnetically stirred for 2-3 min under blue LEDs (light source: 420±10 nm LED, incident light intensity: 0.15 W/cm^2) to give in-situ M/g-C_3N_4*, 0.5 mmol bromobenzene was added. Then the reaction tube was sealed and placed in a constant-temperature bath (25 ^{o}C) to perform the reductive coupling for 20 h under blue light irradiation (light source: 420±10 nm LED, incident light intensity: 0.15 W/cm^2) and argon atmosphere. Once the reaction time was reached, GC analysis provided GC yields of the product with an internal standard.

Experimental procedure for Supplementary Fig. 7b: 15.00 mg Pd/g-C_3N_4 (for the loading of Pd, see Fig. 7b) and 79.50 mg Na_2CO_3 were added to a 10 mL quartz glass tube equipped with 5 mL H_2O, 3 mL 1,4-dioxane and a magnetic stirring under argon atmosphere. After the reaction mixture was magnetically stirred for 2-3 min under blue LEDs (light source: 420±10 nm LED, incident light intensity: 0.15 W/cm^2) to give in-situ Pd/g-C_3N_4*, 0.5 mmol bromobenzene was added. Then the reaction tube was sealed and placed in a constant-temperature bath (25 ^{o}C) to perform the reductive coupling for 20 h under blue light irradiation (light source: 420±10 nm LED, incident light intensity: 0.15 W/cm^2) and argon atmosphere. After reaction, GC analysis of the mixture provided yields of the product with 1,2,3,4-tetramethylbenzene as an internal standard.

1.4.3 Experimental procedure for the reductive coupling with various bases

Experimental procedure for Supplementary Fig. 8a: 15.00 mg Pd/g-C_3N_4 (2.8 wt% Pd) and 1.5 equiv base were added to a 10 mL quartz glass tube equipped with 5 mL H_2O, 3 mL 1,4-dioxane and a magnetic stirring under argon atmosphere. After the reaction mixture was magnetically stirred for 2-3 min under blue LEDs (light source: 420±10 nm LED, incident light intensity: 0.15 W/cm^2) to give in-situ Pd/g-C_3N_4*, 0.5 mmol bromobenzene was added. Then the reaction tube was sealed and placed in a constant-temperature bath (25 ^{o}C) to perform the reductive coupling for 20 h under blue light irradiation (light source: 420±10 nm LED, incident light intensity: 0.15 W/cm^2) and argon atmosphere. After reaction, GC analysis of the mixture provided yields of the product with 1,2,3,4-tetramethylbenzene as an internal standard.

1.4.4 Experimental procedure for the reductive coupling with various solvents

Experimental procedure for Supplementary Fig. 8b: 15.00 mg Pd/g-C_3N_4 (2.8 wt% Pd) and 79.50 mg Na_2CO_3 were added to a 10 mL quartz glass tube equipped with 5 mL H_2O, 3 mL solvent and a magnetic stirring under argon atmosphere. After the reaction mixture was magnetically stirred for 2-3 min under blue LEDs (light source: 420±10 nm LED, incident light intensity: 0.15 W/cm^2) to give in-situ Pd/g-C_3N_4*, 0.5 mmol bromobenzene was added. Then the reaction tube was sealed and placed in a constant-temperature bath (25 ^{o}C) to perform the reductive coupling for 20 h under blue light irradiation (light source: 420±10 nm LED, incident light intensity: 0.15 W/cm^2) and argon atmosphere. After reaction, GC analysis provided yields of the product with 1,2,3,4-tetramethylbenzene as an internal standard.

1.4.5 Experimental procedure for the reductive coupling at different wavelengths

Experimental procedure for Supplementary Fig. 9 and 10: 15.00 mg Pd/g-C_3N_4 (2.8 wt% Pd) and 79.50 mg Na_2CO_3 were added to a 10 mL quartz glass tube equipped with 5 mL H_2O, 3 mL 1,4-dioxane and a magnetic stirring under argon atmosphere. After the reaction mixture was magnetically stirred for 2-3 min under the LED irradiation (light source: 420±10 nm, incident light intensity: 0.15 W/cm^2) to give in-situ Pd/g-C_3N_4*, 0.5 mmol bromobenzene was added. Then the reaction tube was sealed and placed in a constant-temperature bath (25 ^{o}C) to perform the reductive coupling for 20 h under the LED irradiation (incident light intensity: 0.15 W/cm^2. For the wavelength of light, see Supplementary Fig. 9 and 10) and argon atmosphere. Once the reaction time was reached, GC analysis of the mixture provided yields of the product and conversions of the substrate with 1,2,3,4-tetramethylbenzene as an internal standard.

1.4.6 Experimental procedure for the reductive coupling of various aryl halides

Experimental procedure for Supplementary Table 2: 15 mg of Pd/g-C_3N_4 (2.8 wt% Pd) and 79.5 mg of Na_2CO_3 were added to a 20 mL quartz glass tube equipped with 5 mL of H_2O, 3 mL of 1,4-dioxane and a magnetic stirring under argon atmosphere. After the reaction mixture was magnetically stirred for 2-3 min under blue LEDs (light source: 420±10 nm LED, incident light intensity: 0.15 W/cm^2) to give in-situ Pd/g-$C_3N_4^*$, 0.5 mmol aryl halides was added. Then the reaction tube was sealed and placed in a constant-temperature bath (25 ^{o}C) to perform the reductive

coupling for 20 h under blue light irradiation (light source: 420 or 365±10 nm LED, incident light intensity: 0.15 or 0.10 W/cm^2) and argon atmosphere. After reaction, the crude product from four parallel experiments was purified by silica gel chromatography to give the desired product. Note: the obtained isolated yields were often lower than those from gas chromatography under the same conditions, which is due to the loss of the product in the course of the chromatographic purification.

Experimental procedure for Supplementary Table 3: 15 mg of Pd/g-C_3N_4 (2.8 wt% Pd) and 79.5 mg of Na_2CO_3 were added to a 20 mL quartz glass tube equipped with 5 mL of H_2O, 3 mL of 1,4-dioxane and a magnetic stirring under argon atmosphere. After the reaction mixture was magnetically stirred for 2-3 min under blue LEDs (light source: 420±10 nm LED, incident light intensity: 0.15 W/cm^2) to give in-situ Pd/g-$C_3N_4^*$, 0.5 mmol aryl halides was added. Then the reaction tube was sealed and placed in a constant-temperature bath (25 ^oC) to perform the reductive coupling under the irradiation (light source: LED, incident light intensity: 0.15 W/cm^2; for the reaction time and the wavelength of the light, see Supplementary Table 3) and argon atmosphere. After reaction, GC analysis of the mixture provided yields of the product with 1,2,3,4-tetramethylbenzene as an internal standard.

1.5 Experimental procedure for obtaining the AQE

The apparent quantum efficiencies (AQE) of the present coupling reaction were calculated according to the following equation (Equation 1, see Ref. 3 and 4), the rate of biphenyl production (V_{PhPh}), the incident light intensity (according to our measurement results, the actual incident light intensity in the reaction tube was 0.15 W/cm^2) and the actual area of shining light regarding the reaction system (according to our measurement results, the actual area of shining light regarding the reaction system was 9.15 cm^2).

(1) AQE = (2 × number of the produced biphenyl molecules) ÷ (number of incident photons) × 100% = (2 × V_{PhPh}) ÷ [(light intensity × $S_{irradiation}$) ÷ (hc ÷ λ)] × 100%

V_{PhPh}: the rate of biphenyl production. light intensity: the actual incident light intensity (0.15 W/cm^2). $S_{Irradiation}$: the actual area of shining light regarding the reaction system (9.15 cm^2). *h*: planck constant. c: speed of light, λ: wavelength of light.

1.6 Computational details for obtaining the standard redox potential

Computational details for Supplementary Fig. 12a: Standard redox potentials ($E^{\ominus}$) vs. NHE in Supplementary Fig. 12a were obtained based on the equation ($\Delta_r G_m^{\ominus} = -nFE^{\ominus}$, n = 1, F = 96.5 kJ • V^{-1} • mol^{-1}) and the obtained standard molar Gibbs free energy change ($\Delta_r G_m^{\ominus}$, see Supplementary Fig. 13) based on the DFT calculation. All DFT calculations performed by Gaussian 09[5] program in the solution phase (solvent = water) with the IEF-PCM[6,7] solvent model. The B3LYP[8,9] functional with the standard 6-31G (d, p)[10] basis set (SDD basis set for I) was used for geometry optimization and frequency calculation. The B3LYP functional with the 6-311++G(d, p)[11,12] basis set (LANL08 basis set for I) was used to correct the energy for all stationary points. All the optimized stationary points had been identified as minima (zero imaginary frequencies) via the vibrational analysis.

1.7 Procedure for effect of I^- on the reaction and time course of yields of biphenyl

Experimental procedure for Supplementary Fig. 12b: 15 mg of Pd/g-C_3N_4 (2.8 wt% Pd) and 79.5 mg of Na_2CO_3 were added to a 20 mL quartz glass tube equipped with 5 mL of H_2O, 3 mL of 1,4-dioxane and a magnetic stirring under argon atmosphere. After the reaction mixture was magnetically stirred for 2-3 min under blue LEDs (light source: 420±10 nm LED, incident light intensity: 0.15 W/cm^2) to give in-situ Pd/g-$C_3N_4^*$, 0.5 mmol bromobenzene was added (0.5 mmol KI was also added in one of two cases). Then the reaction tube was sealed and placed in a constant-temperature bath (25 ^oC) to perform the reductive coupling for 20 h under blue light irradiation (light source: 420±10 nm LED, incident light intensity: 0.15 W/cm^2) and argon atmosphere. Once the reaction time was reached, GC analysis of the mixture provided the GC yields of the product with 1,2,3,4-tetramethylbenzene as an internal standard.

Experimental procedure for Supplementary Fig. 12c: 15 mg of Pd/g-C_3N_4 (2.8 wt% Pd) and 79.5 mg of Na_2CO_3 were added to a 20 mL quartz glass tube equipped with 5 mL of H_2O, 3 mL of 1,4-dioxane and a magnetic stirring under argon atmosphere. After the reaction mixture was magnetically stirred for 2-3 min under blue LEDs (light source: 420±10 nm LED, incident light intensity: 0.15 W/cm^2) to give in-situ Pd/g-$C_3N_4^*$, 0.5 mmol bromobenzene or iodobenzene was added. Then the reaction tube was sealed and placed in a constant-temperature bath (25 ^oC) to

perform the reductive coupling under blue light irradiation (light source: 420±10 nm LED, incident light intensity: 0.15 W/cm^2) and argon atmosphere. A small amount of reaction liquid was withdrawn every 2 hours to detect the conversion of the substrate by GC.

1.8 Experimental procedure for catalytic recycling test of Pd/g-C_3N_4*

Experimental procedure for Supplementary Fig. 14a: 0.5 mmol bromobenzene, 15 mg of in-situ Pd/g-C_3N_4* (2.8 wt% Pd) and 79.5 mg of Na_2CO_3 were added to a 20 mL quartz glass tube equipped with 5 mL of H_2O, 3 mL of 1,4-dioxane and a magnetic stirring under argon atmosphere. Then the reaction tube was sealed and placed in a constant-temperature bath (25 ^oC) to perform the reductive coupling for 20 h under blue light irradiation (light source: 420±10 nm LED, incident light intensity: 0.15 W/cm^2) and argon atmosphere. After reaction, the mixture was filtered to get solid catalyst. The obtained catalyst was washed with a small amount of water and anhydrous ethanol, dried at 55 ^oC in a vacuum oven, then used as the catalyst for next cycle. After seven cycles, the Pd loading of the recovered catalyst was detected by the inductively coupled plasma–mass spectrometry to be 1.1 wt%. After seven cycles, the recovered catalyst was characterized by TEM analysis to give Supplementary Fig. 15a and b.

Experimental procedure for recycling of Pd/g-C_3N_4* in the absence of Na_2CO_3: 0.5 mmol bromobenzene and 15 mg of in-situ Pd/g-C_3N_4* (2.8 wt% Pd) were added to a 20 mL quartz glass tube equipped with 5 mL of H_2O, 3 mL of 1,4-dioxane and a magnetic stirring under argon atmosphere. Then the reaction tube was sealed and placed in a constant-temperature bath (25 ^oC) to perform the reductive coupling for 20 h under blue light irradiation (light source: 420±10 nm LED, incident light intensity: 0.15 W/cm^2) and argon atmosphere. After reaction, the mixture was filtered to get solid catalyst. The obtained catalyst was washed with a small amount of water and anhydrous ethanol, dried at 55 ^oC in a vacuum oven, then used as the catalyst for next cycle. After seven cycles, the Pd loading of the recovered catalyst was detected by the inductively coupled plasma-mass spectrometry to be 2.0 wt%.

1.9 Procedure for investigating the reproducibility and reliability of the results

Experimental procedure for investigating the catalytic activity of Pd/g-C_3N_4* from different batches (for the results, see Supplementary Table 6): 15.00 mg Pd/g-C_3N_4* (2.8 wt% Pd), 79.50 mg Na_2CO_3 and 0.5 mmol bromobenzene were added to a 10 mL quartz glass tube equipped with 5 mL H_2O, 3 mL 1,4-dioxane and a magnetic stirring under argon atmosphere. Then the reaction tube was sealed and placed in a constant-temperature bath (25 ^oC) to perform the reductive coupling for 20 h under blue light irradiation (light source: 420±10 nm LED, incident light intensity: 0.15 W/cm^2) and argon atmosphere. Once the reaction time was reached, GC analysis of the mixture provided yields of the product and conversions of the substrate with 1,2,3,4-tetramethylbenzene as an internal standard. Note: Each data in Supplementary Table 6 represent the average of six parallel experiments.

Experimental procedure for investigating the catalytic activity of the catalyst in six parallel experiments regarding coupling of bromobenzene: (for the results, see Supplementary Table 7): 15.00 mg Pd/g-C_3N_4 (2.8 wt% Pd) and 79.50 mg Na_2CO_3 were added to a 10 mL quartz glass tube equipped with 5 mL H_2O, 3 mL 1,4-dioxane and a magnetic stirring under argon atmosphere. After the reaction mixture was magnetically stirred for 2-3 min under blue LEDs (light source: 420±10 nm, incident light intensity: 0.15 W/cm^2) to give in-situ Pd/g-C_3N_4*, 0.5 mmol bromobenzene was added. Then the reaction tube was sealed and placed in a constant-temperature bath (25 ^oC) to perform the reductive coupling for 20 h under blue light irradiation (light source: 420±10 nm LED, incident light intensity: 0.15 W/cm^2) and argon atmosphere. Once the reaction time was reached, GC analysis of the mixture provided yields of the product and conversions of the substrate with 1,2,3,4-tetramethylbenzene as an internal standard. Note: six parallel experiments were respectively performed in six reaction tubes at the same time.

1.10 Procedure for investigating the necessity for using Pd/g-C_3N_4*, water, 1,4-dioxane, light and heat

Experimental procedure for Supplementary Fig. 18: 15 mg of Pd/g-C_3N_4 (2.8 wt% Pd) and 79.5 mg of Na_2CO_3 were added to a 20 mL quartz glass tube equipped with 5 mL of H_2O, 3 mL of 1,4-dioxane and a magnetic stirring under argon atmosphere. After the reaction mixture was magnetically stirred for 2-3 min under blue LEDs (light source: 420±10 nm LED, incident light intensity: 0.15 W/cm^2) to give in-situ

$Pd/g\text{-}C_3N_4^*$, 0.5 mmol bromobenzene was added. Then the reaction tube was sealed and placed in a constant-temperature bath (25 ^oC) to perform the reductive coupling for 20 h under blue light irradiation (light source: 420±10 nm LED, incident light intensity: 0.15 W/cm^2) and argon atmosphere. After reaction, GC analysis of the mixture provided the GC yields of the product with 1,2,3,4-tetramethylbenzene as an internal standard. Note: (1) "No H_2O": H_2O was not added. (2) "No $Pd/g\text{-}C_3N_4$*": $Pd/g\text{-}C_3N_4$* was not added. (3) "No 1,4-dioxane": 1,4-dioxane was not added. (4) "No light, 40 or 100 ^oC": the reaction was performed under irradiation-free condition and the reaction temperature was 40 or 100 ^oC.

Experimental procedure for Supplementary Fig. 19: 15 mg of $Pd/g\text{-}C_3N_4$ (2.8 wt% Pd) and 79.5 mg of Na_2CO_3 were added to a 20 mL quartz glass tube equipped with 5 mL of H_2O, 3 mL of 1,4-dioxane and a magnetic stirring under argon atmosphere. After the reaction mixture was magnetically stirred for 2-3 min under blue LEDs (light source: 420±10 nm LED, incident light intensity: 0.15 W/cm^2) to give in-situ $Pd/g\text{-}C_3N_4^*$, 0.5 mmol bromobenzene was added. Then the reaction tube was sealed and placed in a constant-temperature bath (for the temperature, see Supplementary Fig. 19) to perform the reductive coupling for 7 h under blue light irradiation (light source: 420±10 nm LED, incident light intensity: 0.15 W/cm^2) and argon atmosphere. After reaction, GC analysis of the mixture provided the produced amount of the product with 1,2,3,4-tetramethylbenzene as an internal standard.

Experimental procedure for Supplementary Fig. 20: 15 mg of $Pd/g\text{-}C_3N_4$ (2.8 wt% Pd) and 79.5 mg of Na_2CO_3 were added to a 20 mL quartz glass tube equipped with 5 mL of H_2O, 3 mL of 1,4-dioxane and a magnetic stirring under argon atmosphere. After the reaction mixture was magnetically stirred for 2-3 min under blue LEDs (light source: 420±10 nm LED, incident light intensity: 0.15 W/cm^2) to give in-situ $Pd/g\text{-}C_3N_4^*$, 0.5 mmol bromobenzene was added. Then the reaction tube was sealed and placed in a constant-temperature bath (25 ^oC) to perform the reductive coupling under blue light irradiation (light source: LED, 420±10 nm; for the incident light intensity, see Supplementary Fig. 20. The light intensity was measured under the quartz window in the reactor. In other words, the data regarding the light intensity in Supplementary Fig. 20 represent the actual incident light intensity in the reaction tube) and argon atmosphere. After reaction, GC analysis of the mixture provided the produced amount of the product with 1,2,3,4-tetramethylbenzene as an internal standard.

1.11 Procedure for investigating who is the electron donor

Experimental procedure for Supplementary Fig. 21: 15 mg of Pd/g-C_3N_4 (2.8 wt% Pd) and 79.5 mg of Na_2CO_3 were added to a 20 mL quartz glass tube equipped with 5 mL of $H_2{}^{18}O$, 3 mL of 1,4-dioxane and a magnetic stirring under argon atmosphere. After the reaction mixture was magnetically stirred for 2-3 min under blue LEDs (light source: 420±10 nm LED, incident light intensity: 0.15 W/cm^2) to give in-situ Pd/g-$C_3N_4{}^*$, 0.5 mmol bromobenzene was added. Then the reaction tube was sealed and placed in a constant-temperature bath (25 ^oC) to perform the reductive coupling for 20 h under blue light irradiation (light source: 420±10 nm LED, incident light intensity: 0.15 W/cm^2) and argon atmosphere. Once the reaction time was reached, GC analysis of the mixture provided GC yields of the product. Formation of $^{18}O_2$ was confirmed by GC-MS.

1.12 Experimental procedure for effect of the Pd^0 concentration on the reaction

Experimental procedure for Supplementary Fig. 22a: 0.5 mmol bromobenzene, 15 mg of Pd/g-C_3N_4 (2.8 wt% Pd; for the percentage of Pd^0 in Pd, see Fig. 22a) and 79.5 mg of Na_2CO_3 were added to a 20 mL quartz glass tube equipped with 5 mL of $H_2{}^{18}O$, 3 mL of 1,4-dioxane and a magnetic stirring under argon atmosphere. Then the reaction tube was sealed and placed in a constant-temperature bath (25 ^oC) to perform the reductive coupling for 2 h under blue light irradiation (light source: 420±10 nm LED, light intensity: 0.15 W/cm^2) and Ar atmosphere. After reaction, GC and GC-MS analysis of the mixture provided the production amounts of biphenyl and $^{18}O_2$.

Experimental procedure for Supplementary Fig. 22b: 0.5 mmol bromobenzene, 15 mg of Pd/g-C_3N_4 (2.8 wt% Pd) or Pd/g-C_3N_4* (2.8 wt% Pd) and 79.5 mg of Na_2CO_3 were added to a 20 mL quartz glass tube equipped with 5 mL of H_2O, 3 mL of 1,4-dioxane and a magnetic stirring under argon atmosphere. Then the reaction tube was sealed and placed in a constant-temperature bath (25 ^oC) to perform the reductive coupling under blue light irradiation (light source: 420±10 nm LED, incident light intensity: 0.15 W/cm^2) and argon atmosphere. Once the reaction time was reached (for the reaction time, see Supplementary Fig. 22b), the mixture was filtered to get solid catalyst. The catalyst was collected, washed with a small amount of water and anhydrous ethanol, and dried at 55 ^oC in a vacuum oven. Finally, XPS analysis of the obtained catalyst provided percentage of Pd^0 in Pd (Pd^0 + Pd^{II}).

Experimental procedure for Supplementary Table 8: 0.5 mmol bromobenzene, 15 mg of Pd/g-C_3N_4 (2.8 wt% Pd; for the percentage of Pd^0 in Pd, see Supplementary Table 8) and 79.5 mg of Na_2CO_3 were added to a 20 mL quartz glass tube equipped with 5 mL of H_2O, 3 mL of 1,4-dioxane and a magnetic stirring under argon atmosphere. Then the reaction tube was sealed and placed in a constant-temperature bath (25 ^oC) to perform the reductive coupling for 2 h under blue light irradiation (light source: 420±10 nm LED, incident light intensity: 0.15 W/cm^2) and argon atmosphere. After reaction, GC analysis of the mixture provided the produced amount of the benzene byproduct.

1.13 Procedure for investigating the reasons why Na_2CO_3 has a positive effect on the coupling reaction

Experimental procedure for Supplementary Fig. 23a (measurement of H_2O_2): According to previous literature,[13] 0.5 mL volume of 1% o-tolidine in 0.1 mol/L HCl and 2 mL volume of 0.8mg/mL suspension of Pt/g-C_3N_4 in water were added into the sample. After being shaken for 5 min, this mixture turned blue due to the oxidation of *o*-tolidine. Then this mixture was acidified with 1 mol/L HCl (2 mL), and was set for 10 h to give yellow dispersion (for the reaction formulation of the yellow species formation, see Supplementary Fig. 24 and Ref. 14). The yellow dispersion was quickly filtered through a 0.22 um membrane filter and the absorption spectrum of the filtrate was immediately recorded with a UV-Vis spectrophotometer. **Note: Sample A:** 0.4 mmol/L aqueous solution of H_2O_2. **Sample B:** the produced H_2O_2 in the coupling of bromobenzene under the following conditions: 0.5 mmol bromobenzene, 15 mg Pd/g-C_3N_4* (2.8 wt% Pd), 5 mL H_2O, 3 mL 1,4-dioxane, Ar atmosphere, 20 h, light source: 420±10 nm LED, incident light intensity: 0.15 W/cm^2. **Sample C:** the produced H_2O_2 in the coupling of bromobenzene under the following conditions: 0.5 mmol bromobenzene, 15 mg Pd/g-C_3N_4* (2.8 wt% Pd), 1.5 equiv Na_2CO_3, 5 mL H_2O, 3 mL 1,4-dioxane, Ar atmosphere, 20 h, light source: 420±10 nm LED, incident light intensity: 0.15 W/cm^2.

Experimental procedure for Supplementary Fig. 23b: The additive (Na_2CO_3 or Pd/g-C_3N_4* or Na_2CO_3 + Pd/g-C_3N_4*) were added to a 20 mL quartz glass tube equipped with 5 mL of H_2O, 3 mL 1,4-dioxane and 0.5 mmol of H_2O_2. Then the reaction tube was stirred magnetically to perform the H_2O_2 decomposition for 2 h

under blue light (420±10 nm LED, incident light intensity: 0.15 W/cm^2) and argon atmosphere. Once the reaction time was reached, UV-Vis spectroscopy analysis of the remaining H_2O_2 was performed based on the procedure for Supplementary Fig. 23b. The concentration of the remaining H_2O_2 in the reaction system was determined by the calibrated curve and fitting by the calibrated curve and fitting equation (the calibrated curve and fitting equation was made using H_2O_2 with different concentration as external standard materials).

Experimental procedure for Supplementary Fig. 23c: 15 mg of Pd/g-C_3N_4 (2.8 wt% Pd) and Na_2CO_3 were added into a 20 mL quartz glass tube equipped with 5 mL of H_2O, 3 mL of 1,4-dioxane and a magnetic stirring under argon atmosphere. After the reaction mixture was magnetically stirred for 2-3 min under blue light (light source: 420±10 nm incident light intensity: 0.15 W/cm^2) to give in-situ Pd/g-$C_3N_4^*$, 0.5 mmol bromobenzene was added. Then the reaction tube was stirred magnetically to perform the reductive coupling for 4 h under blue light (light source: 420±10 nm LED, incident light intensity: 0.15 W/cm^2) and argon atmosphere. Once the reaction time was reached, GC analysis of the mixture provided the production amount of biphenyl and benzene with 1,2,3,4-tetramethylbenzene as an internal standard. H_2 was detected by another Gas chromatography. The average rates of the [H] and [Ph] production ($\overline{V}_{[H]}$ and $\overline{V}_{[Ph]}$) were calculated based on the equation in Supplementary Fig. 25 and the production amount of PhPh, PhH and H_2 (See Supplementary Table 9).

Experimental procedure for Supplementary Table 9: 15 mg of Pd/g-C_3N_4 (2.8 wt% Pd) and Na_2CO_3 were added into a 20 mL quartz glass tube equipped with 5 mL of H_2O, 3 mL of 1,4-dioxane and a magnetic stirring under argon atmosphere. After the reaction mixture was magnetically stirred for 2-3 min under blue light (light source: 420±10 nm LED, incident light intensity: 0.15 W/cm^2) to give in-situ Pd/g-$C_3N_4^*$, 0.5 mmol bromobenzene was added. Then the reaction tube was stirred magnetically to perform the reductive coupling for 4 h under irradiation (light source: 420±10 nm LED, incident light intensity: 0.15 W/cm^2) and argon atmosphere. Once the reaction time was reached, GC analysis of the mixture provided the production amount of biphenyl and benzene with 1,2,3,4-tetramethylbenzene as an internal standard. H_2 was detected by another Gas chromatography. The average rate of the [H] and [Ph] production ($\overline{V}_{[H]}$ and $\overline{V}_{[Ph]}$) was calculated based on the equation in

Supplementary Fig. 25 and the production amount of PhPh, PhH and H_2 (See Supplementary Table 9).

1.14 Procedure for investigating whether or not the coupling undergoes the SeT mechanism

Experimental procedure for Supplementary Fig. 27: 15 mg of Pd/g-C_3N_4 (2.8 wt% Pd) and 79.5 mg of Na_2CO_3 were added to a 20 mL quartz glass tube equipped with 5 mL of H_2O, 3 mL of 1,4-dioxane and a magnetic stirring under argon atmosphere. After the reaction mixture was magnetically stirred for 2-3 min under blue LEDs (light source: 420±10 nm, incident light intensity: 0.15 W/cm^2) to give in-situ Pd/g-$C_3N_4^*$, 0.5 mmol bromobenzene was added. Then the reaction tube was sealed and placed in a constant-temperature bath (25 ^{o}C) to perform the reductive coupling for 20 h under blue light irradiation (light source: 420±10 nm LED, incident light intensity: 0.15 W/cm^2) and argon atmosphere. Once the reaction time was reached, GC analysis of the mixture provided the GC yields of the product with 1,2,3,4-tetramethylbenzene as an internal standard. Note: (1) 2 equiv. radical inhibitor was added in the case of Supplementary Fig. 27a. (2) The reaction was performed under irradiation-free condition, the loading of Pd/g-C_3N_4* was changed to 1.77g and the reaction temperature was 40 or 100 ^{o}C in the case of Supplementary Fig. 27b.

1.15 Procedure for determining the electron transfer number

Procedure for Supplementary Fig. 29: According to previous literature,[15] The electron transfer number was studied by rotating disk-ring electrodes (RRDE). The Pd/g-$C_3N_4^*$ modified bare glassy carbon electrodes at a rotating speed of 1600 rpm in N_2-saturated solution of Na_2CO_3 (0.1mol/L) in water/1,4-dioxane (V/V = 5:3). The disk potential was set at 0.6 V (vs. SCE) under different light intensity irradiation (λ = 420±10 nm). The ring potential was set at 0.9 V (vs. SCE). The results from RRDE experiments are shown in Supplementary Table 10 and Supplementary Fig. 29.

2. Supplementary notes

2.1 Supplementary Note 1: Reasons why the coupling reaction was very sluggish in spite of an evident photoabsorption of the catalyst at more than 490 nm wavelengths

When the wavelength exceeded the photoabsorption edge (490 nm) of Pd/g-C_3N_4*, the catalyst exhibited an evident photoabsorption (Supplementary Fig. 4), while the

reaction was very sluggish (Supplementary Fig. 9 and 10), which is consistent with the assumption that the coupling reaction is mainly driven by the light-induced separation of electron-hole pairs in the semiconductor. Such a phenomenon is often observed in previous literatures regarding reactions catalyzed by g-C_3N_4-supported Pd or other semiconductor-supported transition metals.[3,16-18]

(1) Why the catalyst exhibited an evident photoabsorption at more than 490 nm wavelengths? When the wavelength exceeds the photoabsorption edge (490 nm) of Pd/g-C_3N_4*, this semiconductor shouldn't exhibit an intrinsic optical absorption that results from the separation of electron-hole pairs of the intrinsic semiconductor because the energy of the photons in these cases is lower than the band-gap energy (2.53 eV) of this semiconductor (Supplementary Fig. 4c).[3,16,17] However, as confirmed by the experimental evidences in previous literatures,[16,17] when the wavelength exceeds the photoabsorption edge of Pd/g-C_3N_4, this semiconductor can display considerable extrinsic optical absorption. In addition, the extrinsic optical absorption of the Pd/g-C_3N_4 semiconductor has been demonstrated in previous literatures[17,4] to be mainly owing to the following two factors: one is the interband electronic transitions of the Pd nanoparticles,[17,19] the other is the electronic transitions from the Pd nanoparticles to the conduction band of the semiconductor.[4] Thus it can be inferred that the photoabsorption of our catalyst (Pd/g-C_3N_4*) at more than 490 nm wavelengths is due to the extrinsic optical absorption that results mainly from the above two factors.

(2) Why the coupling reaction was very sluggish at more than 490 nm wavelengths? (a) According to the above description, when the wavelength exceeds the photoabsorption edge (490 nm) of Pd/g-C_3N_4*, the separation of electron-hole pairs in the intrinsic semiconductor wouldn't occur, and the illumination wouldn't result in an intrinsic optical absorption.[3,16,17] Thus the semiconductor-based photocatalysis wouldn't take place under the irradiation of more than 490 nm light. However, according to the description in previous literatures,[4] it is usually inevitable that there is a small or trace amount of < 490 nm light in > 490 nm LED light, which possibly leads to a weak intrinsic optical absorption and a weak semiconductor-based photocatalysis,[4] which is possibly one of the reasons why a small amount of biphenyl product was produced in the case of using > 490 nm light (see Supplementary Fig. 9a). (b) On the other hand, the irradiation of more than 490 nm light can result in an extrinsic optical absorption (see the above description and Ref. 16 and 17). Thus, it is

possible that the present reaction is driven by the extrinsic optical absorption under the irradiation of more than 490 nm light due to the non-semiconductor photocatalysis (for example, Pd nanoparticles can serve as a photocatalyst via the interband electronic transitions of Pd),[20] which is clearly the important reason why the present coupling reaction can occur under the irradiation of more than 490 nm light. However, the extrinsic optical absorption wouldn't lead to a very effective charge separation/transfer in our photocatalyst based on our experimental evidence: Photocurrent responses of Pd/g-C_3N_4* were very weak under the illumination of more than 490 nm light. Thus the extrinsic optical absorption at more than 490 nm wavelength is not very effective to drive the coupling reaction under our conditions. According to the above reasons, it is obvious that the present light-driven coupling reaction should be very sluggish under the irradiation of more than 490 nm light.

(3) Why the phenomenon (the coupling reaction is very sluggish under the irradiation of more than 490 nm light) coincides with the assumption that the present coupling reaction is mainly driven by the light-induced separation of electron-hole pairs in the semiconductor? When the wavelength exceeds the photoabsorption edge (490 nm) of Pd/g-C_3N_4*, the energy of the photons is lower than the band-gap energy (2.53 eV) of this semiconductor (see Supplementary Fig. 4c), which doesn't allow the separation of electron-hole pairs in the intrinsic semiconductor to occur. Thus, if the present coupling reaction is mainly driven by the light-induced separation of electron-hole pairs in the intrinsic semiconductor, the coupling reaction should be very sluggish under more than 490 nm illumination. Therefore, it is obvious that the phenomenon (the coupling reaction is very sluggish under the irradiation of more than 490 nm light) coincides with the assumption that the present coupling reaction is mainly driven by the light-induced separation of electron-hole pairs in the semiconductor.

2.2 Supplementary Note 2: Reasons why the catalytic efficiency would decrease after four cycling runs

As shown in Supplementary Fig. 14, the catalyst could be recycled for three times with a very slight change in the catalytic activity. The following changes related to the catalyst structure seem to be the main reasons why the catalytic efficiency would decrease after four cycling runs. As seen from the TEM plots (Supplementary Fig. 15 vs. Supplementary Fig. 1a and 2a), the reuse of the catalyst would result in an aggregation of Pd nanoparticles. According to our observation, the loss of the catalyst

was inevitable during recovering the catalyst. In addition, the reuse of the catalyst would lead to a decrease in the Pd loading. For example, when the catalyst was reused for seven times, the Pd loading decreased from 2.8 to 1.1 wt%. Maybe Na_2CO_3 plays an important role in decreasing the Pd loading by promoting the leaching of Pd nanoparticle from the catalyst surface, which is confirmed by the following experimental results: Compared with the reaction condition having Na_2CO_3, base-free condition allowed more Pd nanoparticles (2.0 wt%) to be left on g-C_3N_4 after the catalyst was reused for seven times.

2.3 Supplementary Note 3: Discussion regarding the reproducibility and the reliability of the results

Catalytic activity of Pd/g-C_3N_4* from different batches: In order to clarify the reproducibility and reliability, we tested the catalytic activity of different batches of Pd/g-C_3N_4*. As shown in Supplementary Table 6, when eight different batches of the catalyst were respectively used, the targeted product was obtained in high yields ranged from 92% to 96%, revealing that different batches of the catalyst had a slight difference in the catalytic activity, suggesting that the reported results regarding catalytic activity of Pd/g-C_3N_4* are reliable and reproducible. As seen from Supplementary Fig. 16, for two different batches of Pd/g-C_3N_4*, distribution of the Pd nanoparticles has a slight difference, while the ratio of Pd^0 to Pd^{II} is the similar (Supplementary Fig. 17). These results suggest that small changes in Pd size wouldn't be highly influential of the catalytic activity.

Catalytic activity of the catalyst in six parallel experiments regarding coupling of bromobenzene: In order to clarify the reproducibility of the obtained results from the present catalytic reactions, we performed six parallel experiments with coupling of bromobenzene as the model reaction. Among six parallel experiments (Supplementary Table 7), five gave the targeted product in high yields ranged from 92% to 97%, and one gave an abnormal result (66% yield), which suggests that the reported results regarding the reductive couplings in the present paper are reliable and reproducible when ignoring abnormal results. To improve the reliability of the reported data, all the experiments regarding the reductive couplings were carried out for four times, and all the reported data in the present paper are the average values of the results from four parallel experiments. Note: when calculating the average values, the abnormal results from the parallel experiments were ignored.

3. NMR data regarding the coupling products

Biphenyl (2a, known compound)[21]

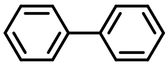

^{1}H NMR (400 MHz, $CDCl_3$): δ (ppm) = 7.58 (d, *J* = 6.6 Hz, 4H), 7.42 (t, *J* = 6.7 Hz, 4H), 7.33 (t, *J* = 6.7 Hz, 2H); ^{13}C NMR (101 MHz, $CDCl_3$): δ (ppm) = 141.3, 128.8, 127.3, 127.2.

4,4'-Dicarbonitrilebiphenyl (2b, known compound)[21]

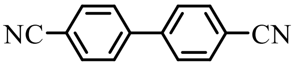

^{1}H NMR (400 MHz, $CDCl_3$): δ (ppm) = 7.80 (d, *J* = 8.3 Hz, 4H), 7.72 (d, *J* = 8.4 Hz, 4H); ^{13}C NMR (101 MHz, $CDCl_3$): δ (ppm) = 143.5, 132.9, 128.0, 118. 5, 112.4.

4,4'-Diformylbiphenyl (2c, known compound)[21]

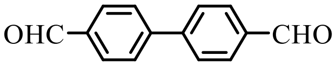

^{1}H NMR (400 MHz, $CDCl_3$): δ (ppm) = 10.11 (s, 2H), 8.02 (d, *J* = 8.4 Hz, 4H), 7.82 (d, *J* = 8.2 Hz, 4H); ^{13}C NMR (101 MHz, $CDCl_3$): δ (ppm) = 191.8, 145.6, 136.0, 130.4, 128.1.

4,4'-Diacetylbiphenyl (2d, known compound)[21]

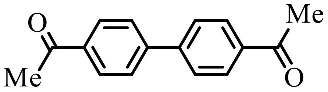

^{1}H NMR (400 MHz, $CDCl_3$): δ (ppm) = 8.08 (d, *J* = 8.3 Hz, 4H), 7.74 (d, *J* = 8.3 Hz, 4H), 2.67 (s, 6H); ^{13}C NMR (101 MHz, $CDCl_3$): δ (ppm) = 197.6, 144.3, 136.6, 129.0, 127.5, 26.7.

4,4'-Dimethylcarboxylatebiphenyl (2e, known compound)[21]

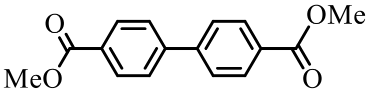

^{1}H NMR (400 MHz, $CDCl_3$): δ (ppm) = 8.15 (d, *J* = 8.2 Hz, 4H), 7.72 (d, *J* = 8.2 Hz, 4H), 3.97 (s, 6H); ^{13}C NMR (101 MHz, $CDCl_3$): δ (ppm) = 166.83, 144.37, 130.22, 129.71, 127.27, 52.25.

4,4'-Difluorobiphenyl (2f, known compound)[21]

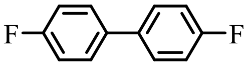

^{1}H NMR (400 MHz, $CDCl_3$): δ (ppm) = 7.54–7.49 (m, 4H), 7.18–7.12 (m, 4H); ^{13}C NMR (101 MHz, $CDCl_3$): δ (ppm) = 162.4 (d, *J* = 246.4 Hz), 136.4 (d, *J* = 3.4 Hz), 128.6 (d, *J* = 8.1 Hz), 115.7 (d, *J* = 21.4 Hz).

4,4'-Dichlorobiphenyl (2g, known compound)[21]

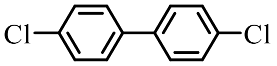

^{1}H NMR (400 MHz, $CDCl_3$): δ (ppm) = 7.50 (td, J_1 = 8.5 Hz, J_2 = 2.3 Hz, 4H), 7.43 (td, J_1 = 8.5 Hz, J_2 = 2.2 Hz, 4H); ^{13}C NMR (101 MHz, $CDCl_3$): δ (ppm) = 138.5, 133.8, 129.1, 128.2.

4,4'-Diethylbiphenyl (2h, known compound)[22]

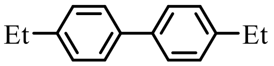

^{1}H NMR (400 MHz, $CDCl_3$): δ (ppm) = 7.50 (d, *J* = 8.1 Hz, 4H), 7.25 (d, *J* = 8.0 Hz, 4H), 2.68 (q, *J* = 7.6 Hz, 4H), 1.26 (t, *J* = 7.6 Hz, 6H); ^{13}C NMR (101 MHz, $CDCl_3$): δ (ppm) = 143.1, 138.7, 128.3, 127.0, 28.6, 15.7.

4,4'-Dimethoxylbiphenyl (2i, known compound)[21]

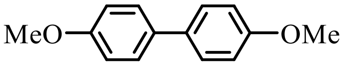

^{1}H NMR (400 MHz, $CDCl_3$): δ (ppm) = 7.51 (d, *J* = 8.7 Hz, 1H), 6.99 (d, *J* = 8.7 Hz, 1H), 3.87 (s, 2H); ^{13}C NMR (101 MHz, $CDCl_3$) δ (ppm) = 158.7, 133.5, 127.8, 114.2, 55.4.

3,3',4,4'-Bis(methylenedioxy)biphenyl (2j, known compound)[23]

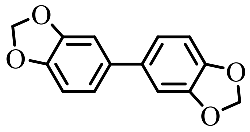

^{1}H NMR (400 MHz, $CDCl_3$): δ (ppm) = 7.01–6.98 (m, 4H), 6.87 (d, *J* = 7.9 Hz, 2H), 6.01 (s, 4H); ^{13}C NMR (101 MHz, $CDCl_3$): δ (ppm) = 148.1, 146.8, 135.4, 120.3, 108.5, 107.6, 101.1.

4,4'-Bis(dimethylamino)biphenyl (2k, known compound)[24]

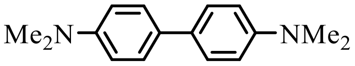

^{1}H NMR (400 MHz, $CDCl_3$): δ (ppm) = 7.51 (d, *J* = 8.7 Hz, 4H), 6.85 (d, *J* = 8.6 Hz, 4H), 3.02 (s, 12H); ^{13}C NMR (101 MHz, $CDCl_3$): δ (ppm) = 149.3, 129.9, 127.0, 113.1, 40.8.

2,2'-Dichlorobiphenyl (2i, known compound)[21]

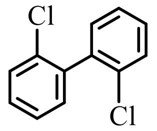

^{1}H NMR (400 MHz, $CDCl_3$): δ (ppm) = 7.50–7.47 (m, 2H), 7.35–7.31 (m, 4H), 7.27–7.25 (m, 2H); ^{13}C NMR (101 MHz, $CDCl_3$): δ (ppm) = 138.37, 133.53, 131.20, 129.46, 129.26, 126.53.

3,3',4,4'-Tetramethylbiphenyl (2m, known compound)[25]

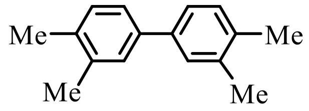

^{1}H NMR (400 MHz, $CDCl_3$): δ (ppm) = 7.37 (s, 2H), 7.33 (d, J = 7.8 Hz, 2H), 7.19 (d, J = 7.7 Hz, 2H), 2.34 (6H, s), 2.31 (6H, s); ^{13}C NMR (101 MHz, $CDCl_3$): 138.9, 136.8, 135.3, 130.0, 128.3, 124.4, 20.0, 19.5.

4,4'-Diacetonitrilebiphenyl (2n, known compound)[24]

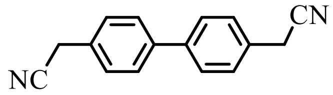

^{1}H NMR (400 MHz, $CDCl_3$): δ (ppm) = 7.62 (d, J = 8.3 Hz, 4H), 7.44 (d, J = 8.4 Hz, 4H), 3.83 (s, 4H); ^{13}C NMR (101 MHz, $CDCl_3$): δ (ppm) = 140.1, 129.3, 128.5, 127.8, 117.8, 23.4.

4,4'-Diyldimethanolbiphenyl (2o, known compound)[26]

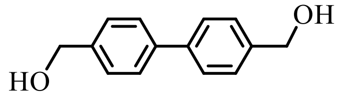

^{1}H NMR (400 MHz, DMSO-d_6): δ (ppm) = 7.62 (d, J = 8.2 Hz, 4H), 7.40 (d, J = 8.2 Hz, 4H), 5.22 (br, 2H), 4.56 (d, J = 12.8 Hz, 4H); ^{13}C NMR (101 MHz, DMSO-d_6): δ (ppm) = 142.1, 138.9, 127.5, 126.7, 63.1.

1,1'-Binaphthalene (2p, known compound)[21]

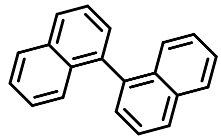

^{1}H NMR (400 MHz, $CDCl_3$): δ (ppm) = 8.00 (dd, J_1 = 8.2 Hz, J_2= 3.5 Hz, 4H), 7.64 (dd, J_1 = 8.2, J_2 = 7.0 Hz, 2H), 7.56–7.50 (m, 4H), 7.46 (d, J = 8.2 Hz, 2H), 7.36–7.31

(m, 2H); ^{13}C NMR (101 MHz, $CDCl_3$): δ (ppm) = 138.5, 133.6, 132.9, 128.2, 127.9, 127.9, 126.6, 126.0, 125.9, 125.4.

2,2'-Binaphthalene (2q, known compound)[24]

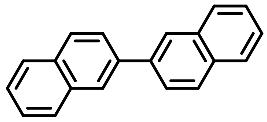

^{1}H NMR (400 MHz, DMSO-d_6): δ (ppm) = 8.22 (s, 2H), 7.91-7.79 (m, 8H), 7.42–7.35 (m, 4H); ^{13}C NMR (101 MHz, DMSO-d_6): δ (ppm) = 137.72, 133.9, 132.8, 129.0, 128.7, 128.0, 127.0, 126.7, 126.1, 125.7.

2,2'-Bipyridine (2r, known compound)[24]

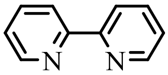

^{1}H NMR (400 MHz, $CDCl_3$): δ (ppm) = 8.69 (d, J = 4.7 Hz, 2H), 8.41 (d, J = 8.0 Hz, 2H), 7.82 (td, J_1 = 7.8 Hz, J_2 = 1.3 Hz, 2H), 7.30 (dt, J_1 = 9.4 Hz, J_2 = 4.5 Hz, 2H); ^{13}C NMR (101 MHz, $CDCl_3$): δ (ppm) = 156.2, 149.2, 136.9, 123.7, 121.1.

3,3'-Bipyridine (2s, known compound)[24]

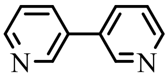

^{1}H NMR (400 MHz, $CDCl_3$): δ (ppm) = 8.84 (d, J = 1.8 Hz, 2H), 8.65 (dd, J_1 = 4.8 Hz, J_2 = 1.5 Hz, 2H), 8.04–7.75 (m, 2H), 7.42–7.39 (m, 2H); ^{13}C NMR (101 MHz, $CDCl_3$): δ (ppm) = 149.3, 148.2, 134.1, 133.5, 123.8.

4,4'-Bipyridine (2t, known compound)[23]

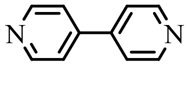

^{1}H NMR (400 MHz, $CDCl_3$): δ (ppm) = 8.73–8.70 (m, 4H), 7.52–7.49 (m, 4H); ^{13}C NMR (101 MHz, $CDCl_3$): δ (ppm) = 150.7, 145.5, 121.4.

3,3'-Bithiophene (2u, known compound)[24]

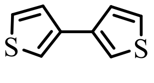

^{1}H NMR (400 MHz, $CDCl_3$): δ (ppm) = 7.41 (dd, J_1 = 2.6 Hz, J_2 = 1.4 Hz, 2H), 7.39–7.35 (m, 4H); ^{13}C NMR (101 MHz, $CDCl_3$): δ (ppm) = 137.3, 126.4, 126.1, 119.8.

4. Supplementary tables

Supplementary Table 1. Quality and suppliers of chemicals

Entry	Compound	Purity	Supplier
1	K_2PdCl_6	98%	Bide Pharmatech Ltd
2	melamine	99.9%	Bide Pharmatech Ltd.
3	1,3,5-triazinane-2,4,6-trione	98%	Bide Pharmatech Ltd.
4	Sodium Carbonate	99.8%	J&K Scientific Ltd.
5	1,4-dioxane	99.5%	J&K Scientific Ltd.
6	1-Bromobenzene	99.5%	Aladdin Chemistry Co., Ltd.
7	1-Iodobenzene	98%	Aladdin Chemistry Co., Ltd.
8	4-Bromoanisole	99%	Aladdin Chemistry Co., Ltd.
9	1-Bromonaphthalene	98%	Aladdin Chemistry Co., Ltd.
10	4-Bromopyridine hydrochloride	98%	Aladdin Chemistry Co., Ltd.
11	3-Bromothiophene	99.09%	Bide Pharmatech Ltd.
12	4-bromo-benzoicacimethylester	99.34%	Bide Pharmatech Ltd.
13	4-Bromoflourobenzene	99.97%	Bide Pharmatech Ltd.
14	4-Bromochlorobenzene	99.92%	Bide Pharmatech Ltd.
15	4-Bromo-1,2-(methylenedioxy)benzene	99.86%	Bide Pharmatech Ltd.
16	2-Chlorobromobenzene	99.88%	Bide Pharmatech Ltd.
17	4-Bromobenzyl alcohol	99.82%	Bide Pharmatech Ltd.
18	2-Bromonaphthalene	98%	Bide Pharmatech Ltd.
19	3-Pyridyl bromide	98%	Bide Pharmatech Ltd.
20	4-Bromobenzonitrile	97%	Aladdin Chemistry Co., Ltd.
21	4-Bromobenzaldehyde	98%	Aladdin Chemistry Co., Ltd.
22	4-Bromophenylacetonitrile	98%	Aladdin Chemistry Co., Ltd.
23	4-Iodobenzaldehyde	98%	Aladdin Chemistry Co., Ltd.
24	4'-Bromoacetophenone	98%	J&K Scientific Ltd.
25	4-Bromoethylbenzene	98%	J&K Scientific Ltd.
26	2-Bromobenzyl cyanide	98%	Shanghai Titan Scientific Co., Ltd.
27	$H_2{}^{18}O$	98%	Aladdin Chemistry Co., Ltd.

Supplementary Table 2. Reductive coupling of various aryl halides under irradiation of 420±10 nm light.[a]

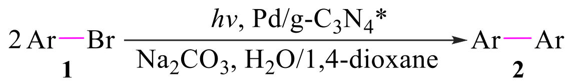

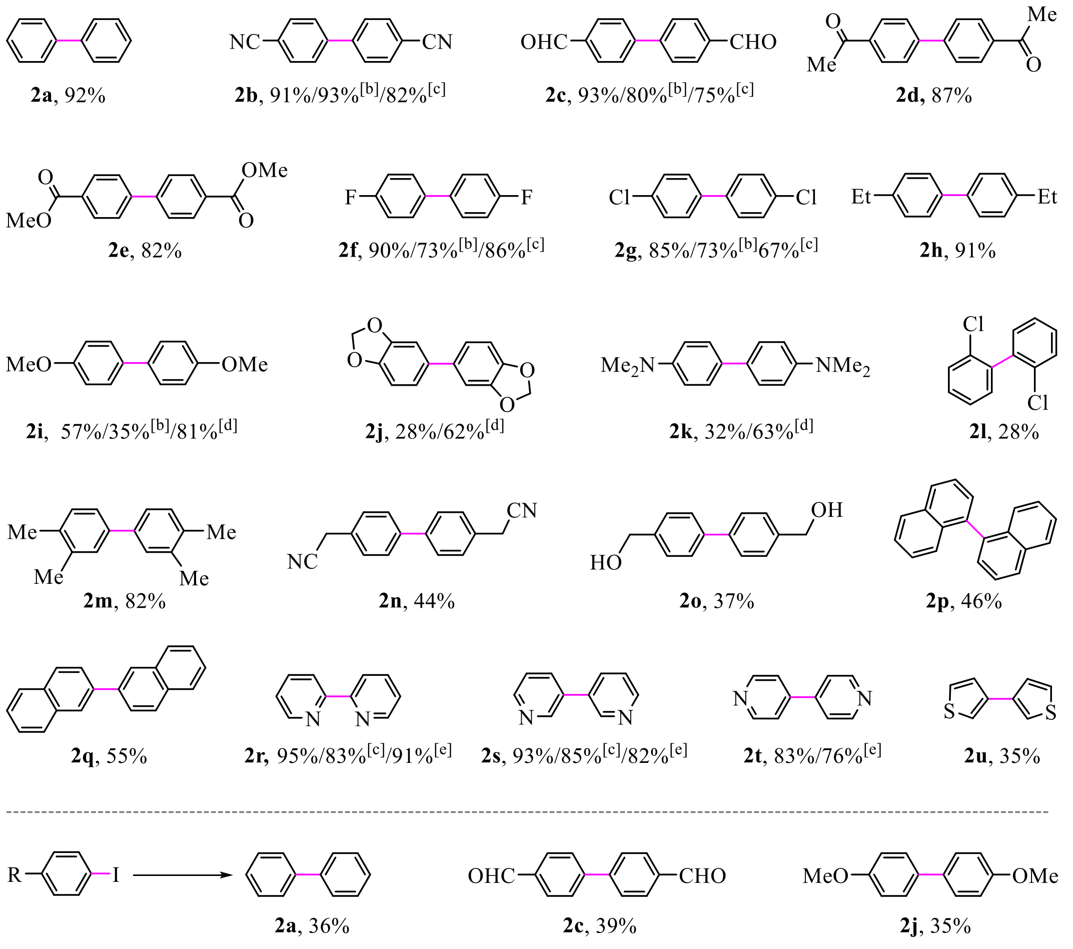

[a] Reaction conditions: 0.5 mmol aryl halides, 15 mg Pd/g-C_3N_4* (2.8 wt% Pd), 1.5 equiv Na_2CO_3, 5 mL H_2O, 3 mL 1,4-dioxane, Ar atmosphere, 20 h, room temperature (25 ^oC), light source: 420±10 nm LED, incident light intensity: 0.15 W/cm^2, all the data shown in this table are the isolated yields unless otherwise specified; [b] 10 h, GC yield; [c] incident light intensity: 0.10 W/cm^2, GC yield; [d] 365±10 nm LED; [e] 3 h, GC yield. Each data in this table represent the average of four parallel experiments. For the detailed conditions and procedure, see Unit 1.4.6.

Supplementary Table 3. Reductive coupling of various aryl halides under irradiation of different lights.[a]

$$2\,\mathrm{Ar{-}Br}\ (\mathbf{1}) \xrightarrow[\mathrm{Na_2CO_3,\ H_2O/1,4\text{-}dioxane}]{h\nu,\ \mathrm{Pd/g\text{-}C_3N_4^*}} \mathrm{Ar{-}Ar}\ (\mathbf{2})$$

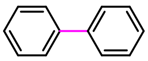 **2a**	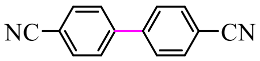 **2b**	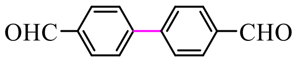 **2c**
420 nm, 23 h, 97%<br>350 nm, 13 h, 96%<br>325-380 nm, 15 h, 93%<br>400-480 nm, 25 h, 98%	420 nm, 12 h, 95%<br>350 nm, 6 h, 92%<br>325-380 nm, 7 h, 93%<br>400-480 nm, 13 h, 96%	420 nm, 13 h, 92%[b]<br>350 nm, 6 h, 56%[c]<br>325-380 nm, 7 h, 60%[d]<br>400-480 nm, 13 h, 89%[e]
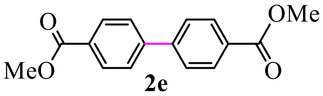 **2e**	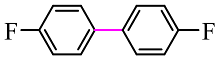 **2f**	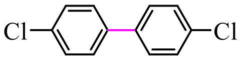 **2g**
420 nm, 17 h, 92%<br>350 nm, 13 h, 90%<br>325-380 nm, 15 h, 91%<br>400-480 nm, 25 h, 95%	420 nm, 15 h, 95%<br>350 nm, 7 h, 93%<br>325-380 nm, 8 h, 92%<br>400-480 nm, 25 h, 96%	420 nm, 16 h, 95%<br>350 nm, 7 h, 92%<br>325-380 nm, 8 h, 91%<br>400-480 nm, 25 h, 97%
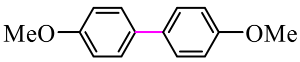 **2i**	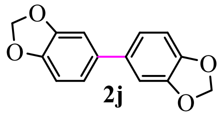 **2j**	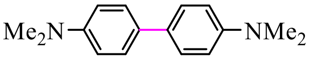 **2k**
420 nm, 35 h, 95%<br>350 nm, 25 h, 91%<br>325-380 nm, 25 h, 93%<br>400-480 nm, 43 h, 96%	420 nm, 42 h, 95%<br>350 nm, 30 h, 93%<br>325-380 nm, 30 h, 90%<br>400-480 nm, 45 h, 94%	420 nm, 40 h, 96%<br>350 nm, 28h, 90%<br>325-380 nm, 28 h, 92%<br>400-480 nm, 45 h, 93%
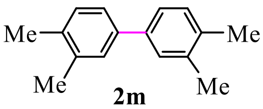 **2m**	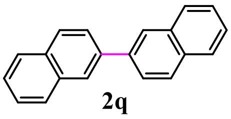 **2q**	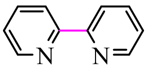 **2r**
420 nm, 25 h, 97%<br>350 nm, 16 h, 96%<br>325-380 nm, 17 h, 93%<br>400-480 nm, 27 h, 98%	420 nm, 35 h, 97%<br>350 nm, 25 h, 96%<br>325-380 nm, 26 h, 93%<br>400-480 nm, 36 h, 98%	420 nm, 3.2 h, 98%<br>350 nm, 2 h, 95%<br>325-380 nm, 2.2 h, 93%<br>400-480 nm, 3.8 h, 97%
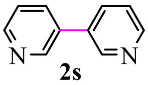 **2s**	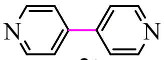 **2t**	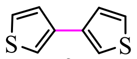 **2u**
420 nm, 3.2 h, 97%<br>350 nm, 2 h, 95%<br>325-380 nm, 2.2 h, 95%<br>400-480 nm, 3.8 h, 96%	420 nm, 3.6 h, 94%<br>350 nm, 2.2 h, 92%<br>325-380 nm, 2.5 h, 93%<br>400-480 nm, 4 h, 97%	420 nm, 40 h, 95%<br>350 nm, 30 h, 91%<br>325-380 nm, 31 h, 92%<br>400-480 nm, 41 h, 93%

[a] Reaction conditions: 0.5 mmol aryl halides, 15 mg Pd/g-C_3N_4* (2.8 wt% Pd), 1.5 equiv Na_2CO_3, 5 mL H_2O, 3 mL 1,4-dioxane, Ar atmosphere, room temperature (25 ^{o}C), light source: LED,

incident light intensity: 0.15 W/cm^2. All the data shown in this table are GC yields. Note: x nm, y h and z% represent wavelength of light, reaction time and GC yield of the targeted product. Note: x nm in this table represents x ±10 nm. Note: conversions of substrates in all experiments in this table were close to 100%. [b] 4,4'-Bibenzoic acid and 4'-formylbiphenyl-4-carboxylic acid were obtained in 1% and 5% yield, respectively. [c] 4,4'-Bibenzoic acid and 4'-formylbiphenyl-4-carboxylic acid were obtained in 7% and 33% yield, respectively. [d] 4,4'-Bibenzoic acid and 4'-formylbiphenyl-4-carboxylic acid were obtained in 6% and 30% yield, respectively. [e] 4,4'-Bibenzoic acid and 4'-formylbiphenyl-4-carboxylic acid were obtained in 1% and 6% yield, respectively. For the detailed conditions and procedure, see Unit 1.4.6.

Supplementary Table 4. Comparison of our method against other methods.

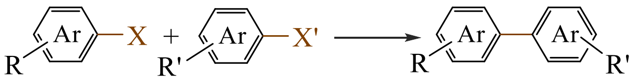

Entry	Ref.	X or X'	Reductant	Catalyst	Energy	T/ ℃	Time/h
1	**This work**	Br, I	H_2O	Pd/g-C_3N_4	*hv* (420 nm)	RT	3-20
2	27	I	MeOH	AuPd@NRCN	*hv* (460 nm)	RT	24
3	28	Br, I	(*i*-Pr)$_2$NH	AuNP/KNb_3O_8	*hv* (UVA)[a]	RT	2-24
4	29	I	NEt_3	[Au_2(dppm)$_2$]Cl_2	*hv* (365 nm)	RT	16
5	30	Cl, Br, I	NEt_3	TiO_2-AA-Pd	*hv* (CFL)[c] and heat	100	3-10
6	31	Cl	EtOH	Au-Pd@NMCI	Heat	RT	6-12
7	32	Br, I	TDAE[b]	Ligand/$NiBr_2$/CoPc	Heat	80	24
8	33	Br, I	Mg	$Ti_{0.97}Pd_{0.03}O_{1.97}$	Heat	100	1-4.5
9	34	I	N_2H_2	Pd(OAc)$_2$	Heat	RT	8
10	35	Br	*t*-BuLi	$FeCl_3$	Heat	-78	0.5
11	36	Cl, Br, I	Glucose	Pd/Fe_3O_4@PDA	Heat	100-120	24
12	37	Cl, Br, I	HCO_2Na	Pd(0)-RGO	Heat	80	5
13	13	Cl, Br, I	Sm	CuCl	Heat	reflux	10-20
14	24	Cl, Br, I	N_2H_2	PMe_3/Ni(cod)$_2$	Heat	110	12
15	38	Cl, Br, I	*i*-PrOH	Pd_5Au_5/NCB	Heat	40	1-3
16	39	Cl, Br, I	EtOH	Pd(OAc)$_2$	Heat	80	3-12
17	40	Br	Hydroquinone	[NBu_4][Pd(DMSO)Cl_3]	Heat	100-140	3-72
18	41	Br, I	Ascorbic acid	PdNPs@*β*-CD	Heat	70	24
19	42	Br, I	Li_2MnR_4	No	Heat	-78	10

[a] UVA: ultraviolet radiation a; [b] TDAE: tetrakis(dimethylamino)ethylene; [c] CFL: visible light from compact fluorescent light.

Supplementary Table 5. Comparison of our method against other methods.

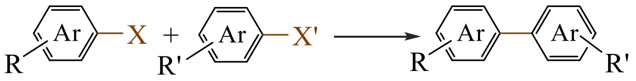

Entry	Ref.	Solvent	Base or additive	Ar	Tested groups (R or R')	Yield/%
1	**This work**	H_2O/dioxane	$NaCO_3$	Phenyl, naphthyl, thienyl, pyridinyl	CHO, C(O)R, CO_2R, CN, CH_2CN, F, Cl, OR, CH_2OH, NR_2, Alkyl	28-95
2	27	MeOH	NaOH	Phenyl	CN, Cl, OR, NO_2, CF_3, Alkyl	0-96
3	28	MeOH	(i-Pr)$_2$NH	Phenyl	Cl, OR, NO_2, Alkyl	21-99
4	29	MeCN/MeOH	K_2HPO_4	Phenyl	CHO, C(O)R, CO_2R, CN, TfO, Cl, NO_2, OR, Alkyl	14-85
5	30	No	NEt_3	Phenyl	Br, OR, NO_2, Alkyl	65-95
6	31	H_2O/EtOH	K_2CO_3	Phenyl, thienyl	C(O)R, OR, NO_2, OH, Alkyl	72-100
7	32	Dioxane	4Å MS	Phenyl, naphthyl	CO_2R, P(O)(OR)$_2$, P(O)R$_2$, C(O)NR$_2$	19-94
8	33	H_2O	No	Phenyl	CN, F, Cl, OR, NH_2, Alkyl	45-99
9	34	DMF/DMSO	K_3PO_4	Phenyl, pyridinyl	CN, F, Cl, Br, OR,CF_3	74-92
10	35	Et_2O	(t-BuO)$_2$	Phenyl, naphthyl, phenanthryl	F, NR_2, OR, CF_3TMS, Alkyl	11-90
11	36	H_2O	Cs_2CO_3	Phenyl, pyridinyl	F, CN, OR, NO_2, Alkyl	58-92
12	37	H_2O or DMF	KOH	Phenyl	H	79-97
13	13	THF	KI	Phenyl, thienyl	Cl, OR, Alkyl	45-62
14	24	Dioxane	K_3PO_4	Phenyl, thienyl, pyridinyl	CO_2R, F, CH_2CN, NR_2, OR, CH(OEt)$_2$, Ph, Alkyl	63-99
15	38	H_2O/i-PrOH	K_2CO_3	Phenyl	CHO, NO_2, OR, CN, OH, Alkyl	81-97
16	39	WEPA[a]/EtOH	No	Phenyl, pyridinyl, phenanthryl	C(O)R, F, Cl, OR, NO_2, Alkyl	25-99
17	40	DMF	K_3PO_4	Phenyl	C(O)R, CN, CF_3, OR, Alkyl	17-95
18	41	H_2O	K_2CO_3	Phenyl	C(O)R, OR, NO_2, Alkyl	40-96
19	42	THF	No	Phenyl, naphthyl, pyridinyl	CO_2R, F, OR, CF_3, Alkyl	33-87

[a] WEPA: pomegranate ash.

Supplementary Table 6. Coupling of bromobenzene catalyzed by different batches of Pd/g-C_3N_4*[a]

$$\mathrm{Ph{-}Br} \xrightarrow[\mathrm{Pd/g\text{-}C_3N_4^*,\ dioxane,\ 20\ h}]{\mathrm{H_2O},\ h\nu,\ \mathrm{Na_2CO_3}} \mathrm{Ph{-}Ph}$$

Batch number	1	2	3	4	5	6	7	8
Yield of biphenyl	96%	92%	93%	95%	92%	92%	93%	95%

[a] Each data in this table represent the average of six parallel experiments. The loading of Pd on g-C_3N_4 is 2.8 wt%. For the experimental conditions and procedure, see Unit 1.9.

Supplementary Table 7. Parallel experiments regarding coupling of bromobenzene[a]

$$\mathrm{Ph{-}Br} \xrightarrow[\mathrm{Pd/g\text{-}C_3N_4^*,\ dioxane,\ 20\ h}]{\mathrm{H_2O},\ h\nu,\ \mathrm{Na_2CO_3}} \mathrm{Ph{-}Ph}$$

Experiment number	1	2	3	4	5	6
Yield of biphenyl	96%	92%	66%	97%	95%	92%

[a] For the experimental conditions and procedure, see Unit 1.9.

Supplementary Table 8. Effect of the Pd^0 concentration on the benzene byproduct[a]

$$\mathrm{Ph{-}Br} \xrightarrow[\mathrm{Pd/g\text{-}C_3N_4^*,\ Dioxane,\ 2\ h}]{\mathrm{H_2O},\ h\nu,\ \mathrm{Na_2CO_3}} \mathrm{Ph{-}Ph + Ph{-}H}$$

Percentage of Pd^0 in Pd (Pd^0 + Pd^{II}) of Pd/g-C_3N_4	54%	61%	73%	85%	91%
Benzene byproduct/μmol	14.6	8.7	2.3	1.8	0.4

[a] Each data in this table represent the average of four parallel experiments. For the experimental conditions and procedure, see Unit 1.12.

Supplementary Table 9. Effect of the Na_2CO_3 loading on the reaction[a]

$$\mathrm{Ph{-}Br} \xrightarrow[\mathrm{Pd/g\text{-}C_3N_4^*,\ Dioxane,\ 4\ h}]{\mathrm{H_2O},\ h\nu,\ \mathrm{Na_2CO_3}} \mathrm{Ph{-}Ph + Ph{-}H + H_2 + O_2}$$

Na_2CO_3/μmol	Biphenyl/μmol	H_2/μmol	Benzene/μmol	$\overline{V}_{[H]} / \overline{V}_{[Ph]}$
100	16.2	27.2	9.5	1.5
250	48.0	43.3	7.3	0.9
750	91.9	31.1	4.1	0.4
1500	88.5	22.8	2.7	0.3

[a] Ratio of $\overline{V}_{[H]}$ to $\overline{V}_{[Ph]}$ was calculated by the equation in Supplementary Fig. 25. Each data in this table represent the average of four parallel experiments. For the experimental conditions and procedure, see Unit 1.13.

Supplementary Table 10. Results from RRDE experiments[a]

Light intensity (mW cm^{-2})	I_{disk} (μA)	I_{ring} (μA)	n
0	0	0	0
2.63	0.59	0.021	3.53
5.72	0.79	0.031	3.49
8.45	1.30	0.046	3.54
11.78	1.89	0.071	3.51

[a] The electron transfer numbers (n) were calculated from equation: $n = 4I_{disk}/(I_{disk} + I_{ring}/N)$ where N is the RRDE collection efficiency, measured to be 0.27. For the experimental conditions and procedure, see Unit 1.15.

5. Supplementary figures

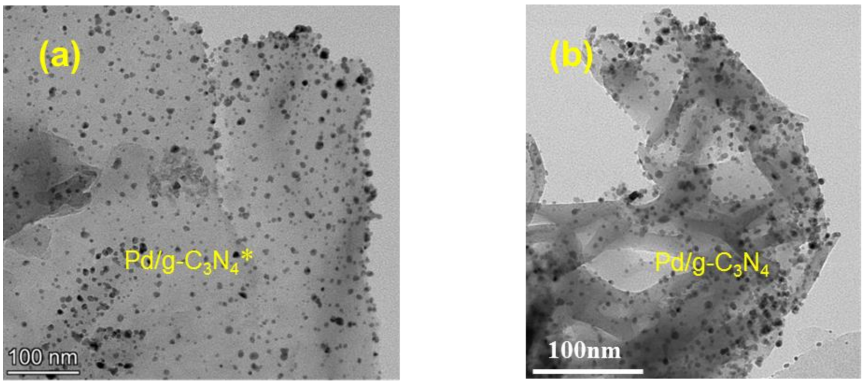

Supplementary Fig. 1 TEM images. (a) TEM images of Pd/g-C_3N_4* (2.8 wt% Pd). (b) TEM images of Pd/g-C_3N_4 (2.8 wt% Pd). Pd/g-C_3N_4: graphite phase carbon nitride-palladium, Pd/g-C_3N_4*: the activated Pd/g-C_3N_4 by the light-irradiated treatment.

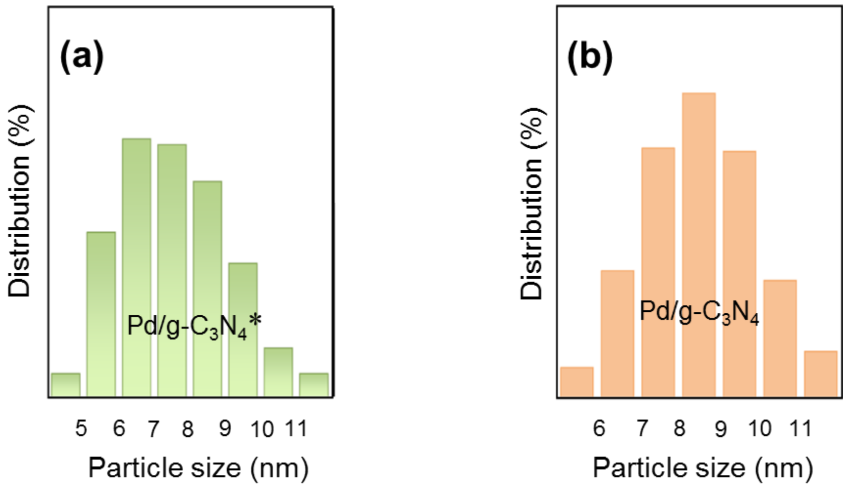

Supplementary Fig. 2 Particle size distribution. (a) Particle size distribution of Pd/g-C_3N_4* (2.8 wt% Pd). (b) Particle size distribution of Pd/g-C_3N_4 (2.8 wt% Pd). Source data are provided as a Source Data file.

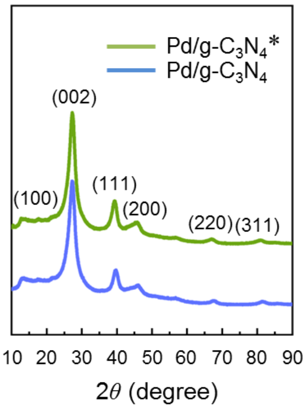

Supplementary Fig. 3 X-ray diffraction (XRD). XRD of Pd/g-C_3N_4* (green, 2.8 wt% Pd) and Pd/g-C_3N_4 (blue, 2.8 wt% Pd). Source data are provided as a Source Data file.

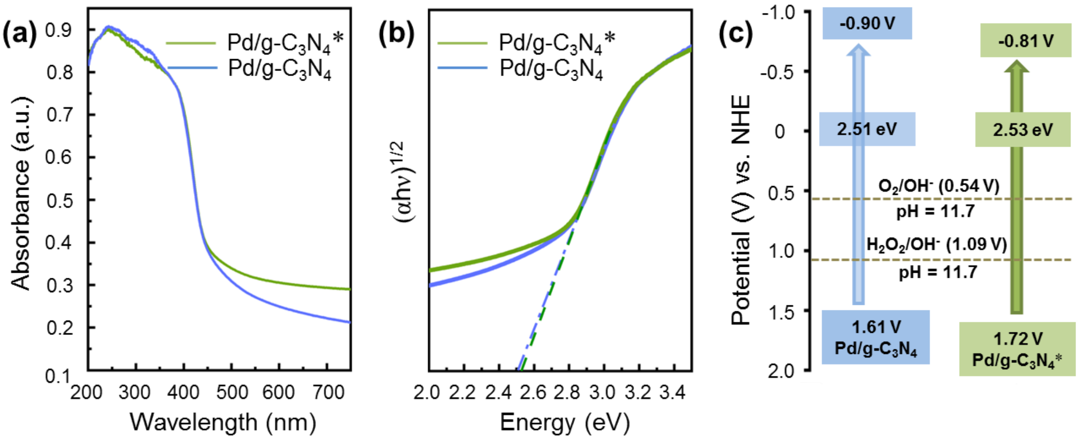

Supplementary Fig. 4 Samples of Pd/g-C_3N_4 (2.8 wt% Pd) and Pd/g-C_3N_4* (2.8 wt% Pd). (a) UV-visible diffuse reflection spectra of Pd/g-C_3N_4 (blue curve, 2.8 wt%) and Pd/g-C_3N_4* (green curve, 2.8 wt%). a.u.: arbitrary units. Source data are provided as a Source Data file. (b) Tauc plots of Pd/g-C_3N_4 (blue curve, 2.8 wt%) and Pd/g-C_3N_4* (green curve, 2.8 wt%). Source data are provided as a Source Data file. (c) Energy-band positions of Pd/g-C_3N_4 (blue, 2.8 wt%) and Pd/g-C_3N_4* (green, 2.8 wt%). Note: pH value of the reaction system is 11.7 under our conditions. Standard electrode potentials (E^{θ}) of O_2/OH^- and H_2O_2/OH^- are 0.40 and 0.95 V, respectively (see Ref. 15 and 43). Thus their redox potentials at pH = 11.7 are calculated to be 0.54 and 1.09 V, respectively, based on Nernst equation.

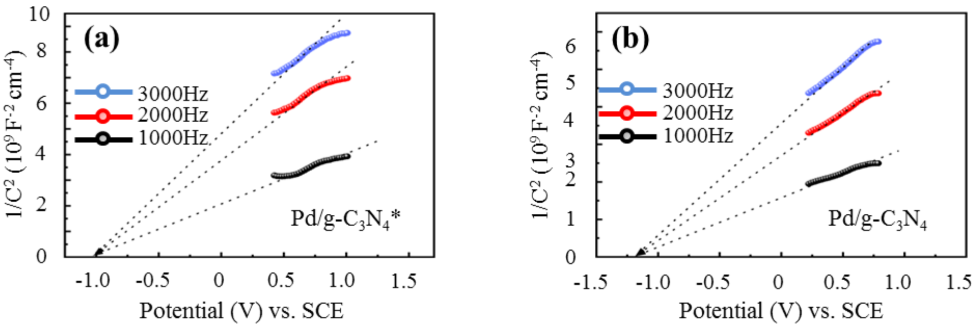

Supplementary Fig. 5 Electrochemical Mott-Schottky plots. (a) Electrochemical Mott-Schottky plots of Pd/g-C_3N_4 (2.8 wt% Pd) in 0.5 mol/L aqueous solution of Na_2SO_4. (b) Electrochemical Mott-Schottky plots of Pd/g-C_3N_4* (2.8 wt% Pd) in 0.5 mol/L aqueous solution of Na_2SO_4. For the measurement conditions, see Unit 1.3. 3000Hz: blue curve, 2000Hz: red curve, 1000Hz: black curve. Source data are provided as a Source Data file.

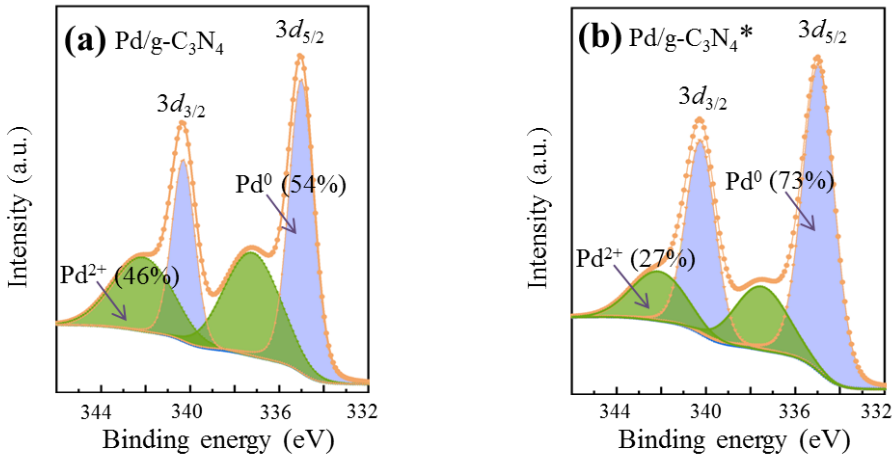

Supplementary Fig. 6 X-ray photoelectron spectroscopy (XPS). XPS spectra of (a) Pd/g-C_3N_4 and (b) Pd/g-C_3N_4*. Pd^{2+}: green, Pd^{0}: light purple. Source data are provided as a Source Data file.

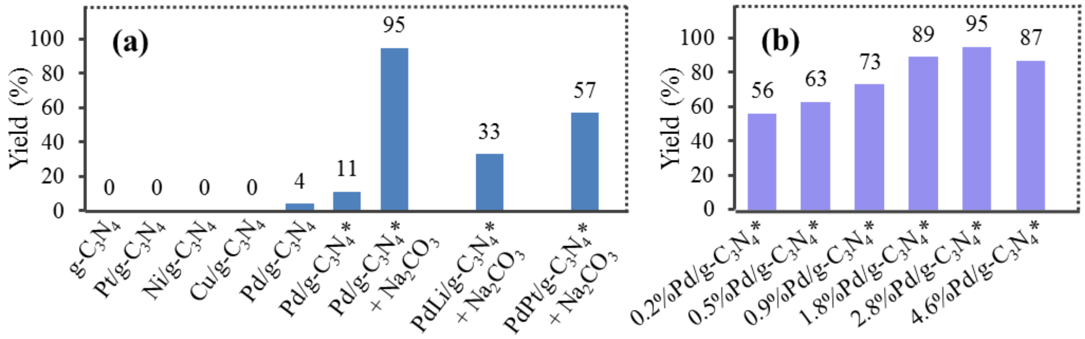

Supplementary Fig. 7 Coupling of bromobenzene with various catalysts. (a) Effect of different photocatalyst systems on the reaction. The loading of the metal on g-C_3N_4 is 2.8 wt% in all cases. M/g-C_3N_4: graphite phase carbon nitride-supported metal, M/g-C_3N_4*: the activated Pd/g-C_3N_4 by the light-irradiated treatment. (b) Effect of different Pd loading amounts on the reaction. Yield: yield of biphenyl. x%Pd/g-C_3N_4* means that the loading of Pd on g-C_3N_4 is x wt%. Note: Each data in this figure represent the average of four parallel experiments. For the experimental conditions and procedure, see Unit 1.4.2. Source data are provided as a Source Data file.

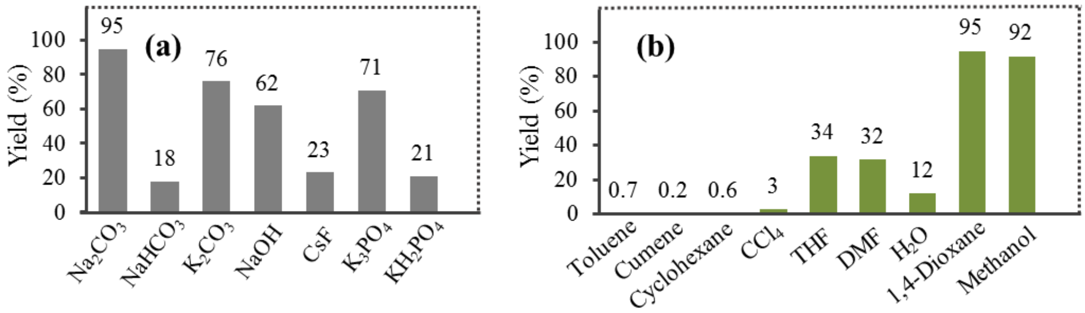

Supplementary Fig. 8 Coupling of bromobenzene with various bases or solvents. (a) Effect of different bases on the reaction. Yield: yield of biphenyl. (b) Effect of different solvents on the reaction. Yield: yield of biphenyl. THF represents tetrahydrofuran. DMF represents *N*,*N*-dimethylformamide. For the experimental conditions and procedure, see 1.4.3 and 1.4.4. Note: Each data in this figure represent the average of four parallel experiments. Source data are provided as a Source Data file.

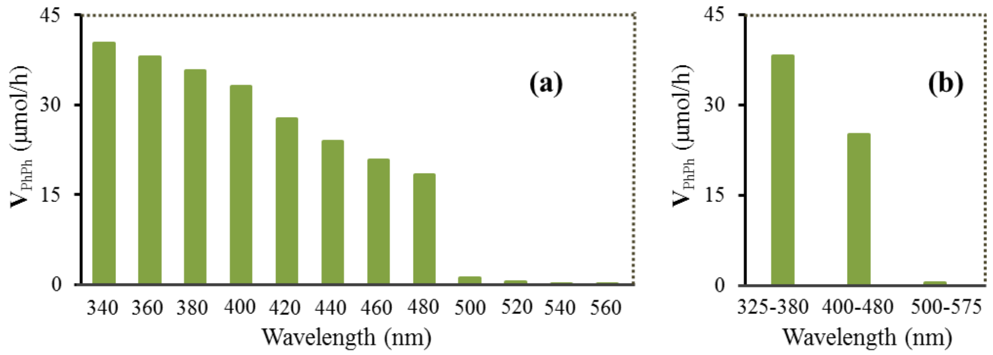

Supplementary Fig. 9 Produced rate of biphenyl under different wavelengths of light. (a) Effect of different monochromatic lights on the produced rate of biphenyl. x nm wavelength in represents x ±10 nm wavelength because the actual wavelength of the light is x ±10 nm in the case of using x nm LED light source. (b) Effect of different polychromatic lights on the produced rate of biphenyl. For the experimental conditions and procedure, see Unit 1.4.5. V_{PhPh}: average rate regarding production of biphenyl (PhPh) in 2 h. Source data are provided as a Source Data file.

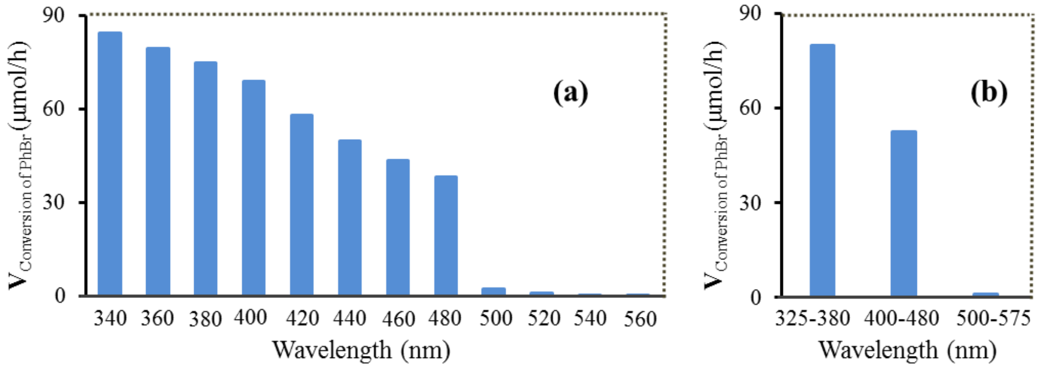

Supplementary Fig. 10 Rate regarding conversion of bromobenzene under different wavelengths of light. (a) Conversion of bromobenzene under illumination of different monochromatic lights. x nm wavelength in represents x ±10 nm wavelength because the actual wavelength of the light is x ±10 nm in the case of using x nm LED light source. (b) Conversion of bromobenzene under illumination of different polychromatic lights. $V_{conversion\ of\ PhBr}$: average rate regarding conversion of bromobenzene (PhBr) in 2 h. For the experimental conditions and procedure, see Unit 1.4.5. Source data are provided as a Source Data file.

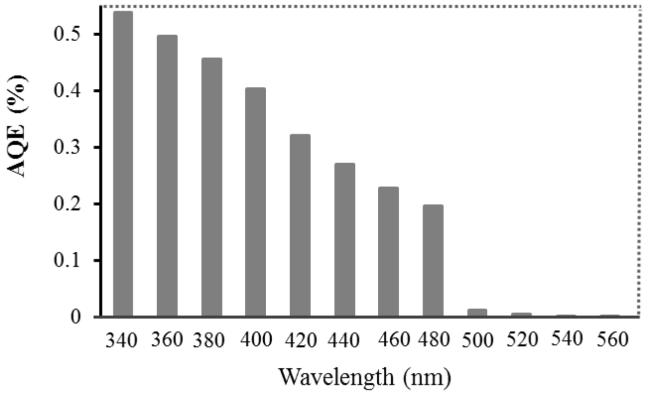

Supplementary Fig. 11 Apparent quantum efficiencies (AQE) of the coupling. The AQE value in this figure was calculated based on the rate of biphenyl production in Supplementary Fig. 9a and the procedure in Unit 1.5 of this supporting information. x nm wavelength in this figure represents x ±10 nm wavelength. Source data are provided as a Source Data file.

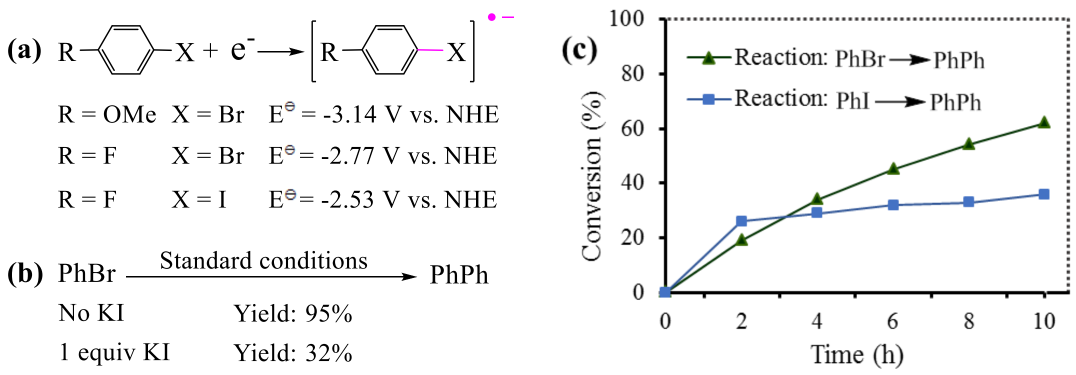

Supplementary Fig. 12 Effect of substituents or halogen ions. (a) Standard redox potential ($E^{\ominus}$) vs. NHE of phenyl halides with different substituents. (b) Effect of I^- on the reaction. (c) Time course of the conversion of bromobenzene (green) and iodobenzene (blue). PhBr: bromobenzene, PhI: iodobenzene, PhPh represents biphenyl. Source data are provided as a Source Data file. Each data in Supplementary Fig. 12b and 12c represent the average of four parallel experiments. For the experimental conditions and procedure, see Unit 1.6 and 1.7.

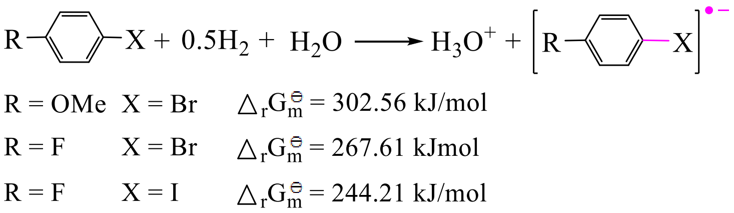

Supplementary Fig. 13 Standard molar Gibbs free energy change ($\Delta_r G_m^{\ominus}$). The obtained standard molar Gibbs free energy change from DFT calculations. For the obtained procedure, see Unit 1.6.

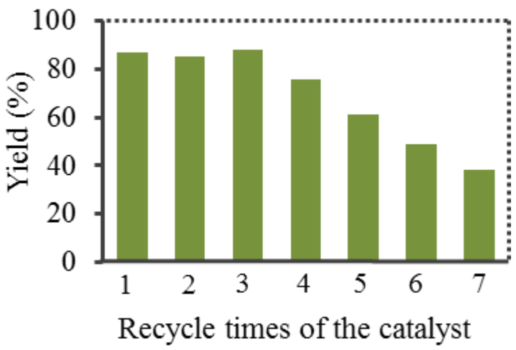

Supplementary Fig. 14 Recycling test of Pd/g-C_3N_4*. For the experimental conditions and procedure, see Unit 1.8. Source data are provided as a Source Data file.

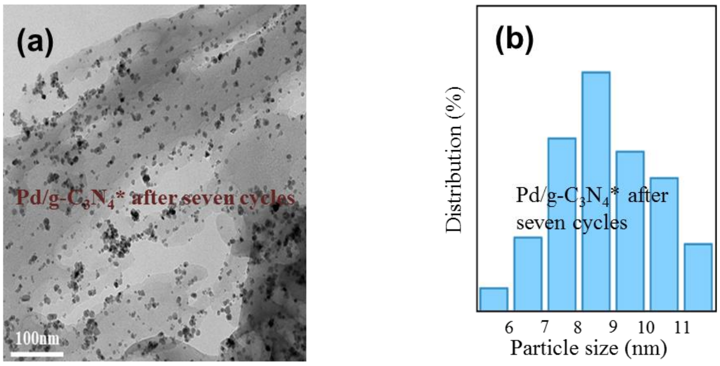

Supplementary Fig. 15 TEM image and particle size distribution. (a) TEM images of Pd/g-C_3N_4* after seven cycles. (b) Particle size distribution of Pd/g-C_3N_4* after seven cycles (Source data are provided as a Source Data file). For the experimental conditions and procedure, see Unit 1.8.

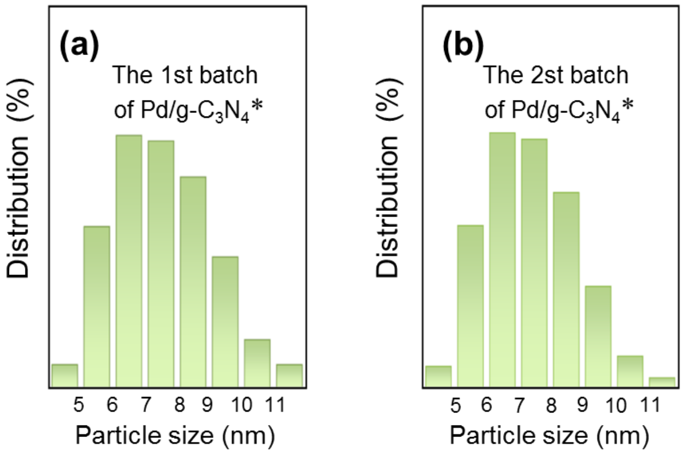

Supplementary Fig. 16 Particle size distribution. (a) Particle size distribution regarding the 1st batch of Pd/g-C_3N_4* in Supplementary Table 6. Fig. 16a is the same figure as Fig. 2a. (b) Particle size distribution regarding the 2st batch of Pd/g-C_3N_4* in Supplementary Table 6. Source data are provided as a Source Data file.

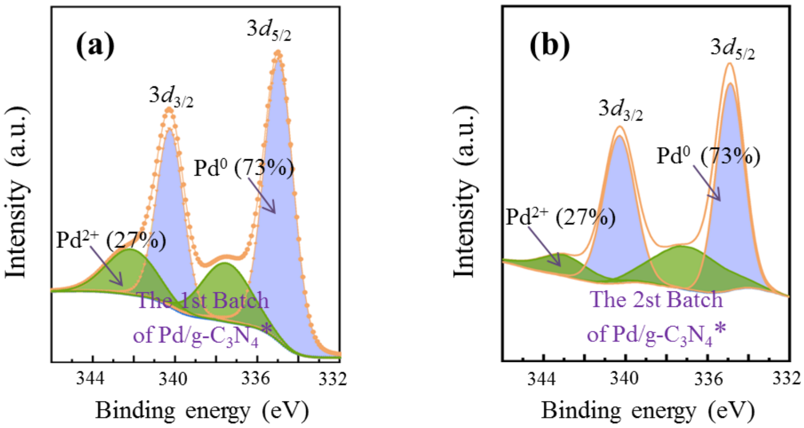

Supplementary Fig. 17 X-ray photoelectron spectroscopy (XPS) for different batches of catalyst. (a) XPS spectra regarding the 1st batch of Pd/g-C_3N_4* in Supplementary Table 6. Fig. 17a is the same figure as Fig. 6b. (b) XPS regarding the 2st batch of Pd/g-C_3N_4* in Supplementary Table 6. Pd^{2+}: green, Pd^0: light purple. Source data are provided as a Source Data file.

$$2\,PhBr \xrightarrow{\text{Standard conditions}} PhPh$$

Condition	Conv.	Yield
No H_2O	5%	trace
No Pd/g-C_3N_4*	< 1%	No
No 1,4-dioxane	18%	15%
No light, 40 or 100 °C	1-6%	No

Supplementary Fig. 18 Several control experiments. PhBr: bromobenzene, PhPh: biphenyl, Conv.: conversion of bromobenzene, Yield: yield of biphenyl. Standard conditions: 0.5 mmol bromobenzene, 15 mg Pd/g-C_3N_4* (2.8 wt%), 1.5 equiv Na_2CO_3, 5 mL H_2O, 3 mL 1,4-dioxane, 20 h, Ar atmosphere, light source: 420±10 nm LED, incident light intensity: 0.15 W/cm^2. Note: Each data in this figure represent the average of four parallel experiments. For the experimental conditions and procedure, see Unit 1.10.

$$2\,PhBr \xrightarrow[\text{2 h, } h\nu]{\text{Standard conditions}} PhPh$$

Temperature	Conv.	$\bar{V}_{PhPh}$
0 °C	16%	18 μmol/h
25 °C (RT)	21%	25 μmol/h
50 °C	27%	32 μmol/h
80 °C	39%	46 μmol/h

Supplementary Fig. 19 Effect of temperature on the reaction. PhBr: bromobenzene, PhPh: biphenyl, Conv.: conversion of bromobenzene, $\bar{V}_{PhPh}$: the average rate of the biphenyl production in 2 h. Standard conditions: 0.5 mmol bromobenzene, 15 mg Pd/g-C_3N_4* (2.8 wt%), 1.5 equiv Na_2CO_3, 5 mL H_2O, 3 mL 1,4-dioxane, 20 h, Ar atmosphere, light source: 420±10 nm LED, incident light intensity: 0.15 W/cm^2. Note: Each data in this figure represent the average of four parallel experiments. For the experimental procedure, see Unit 1.10.

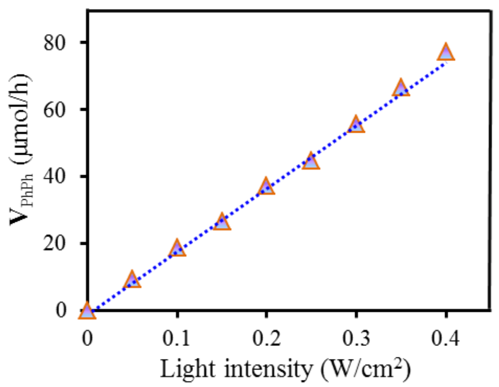

Supplementary Fig. 20 Dependence of biphenyl yield on the incident light intensity. V_{PhPh}: rate regarding production of biphenyl (PhPh). Selectivities of biphenyl product ranged from 97% to 98% in all experiments. For the experimental procedure, see Unit 1.10. Source data are provided as a Source Data file.

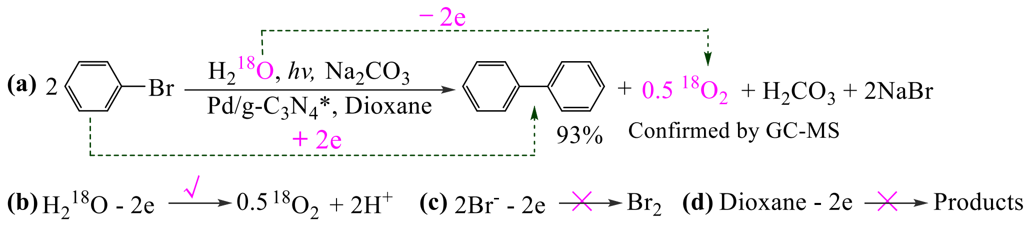

Supplementary Fig. 21 Investigation on who is the electron donor. (a) Reductive coupling of bromobenzene. (b) Half-reaction of water oxidation. (c) Half-reaction of Br^- oxidation. (d) Half-reaction of dioxane oxidation. For the experimental conditions and procedure, see Unit 1.11.

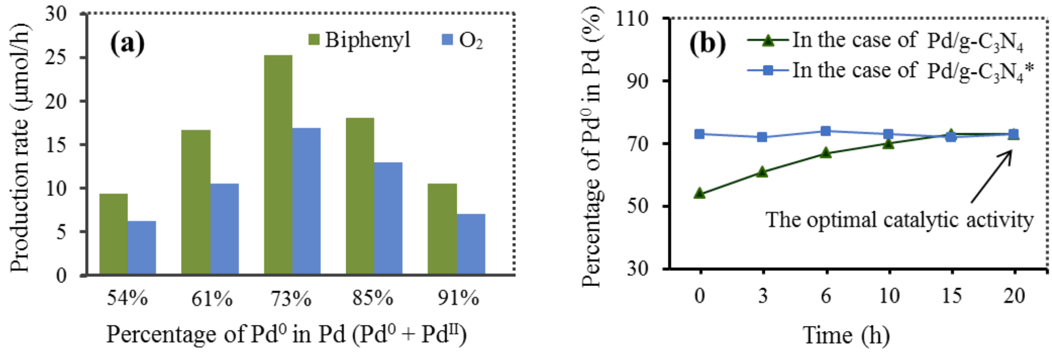

Supplementary Fig. 22 Relationship between the Pd^0 concentration and the coupling. (a) Effect of the Pd^0 concentration on the average rate of the biphenyl (green) and O_2 (blue). (b) Time course of the Pd^0 concentration in the case of Pd/g-C_3N_4 (green) or Pd/g-C_3N_4* (blue). For the experimental procedure, see Unit 1.12. Source data are provided as a Source Data file.

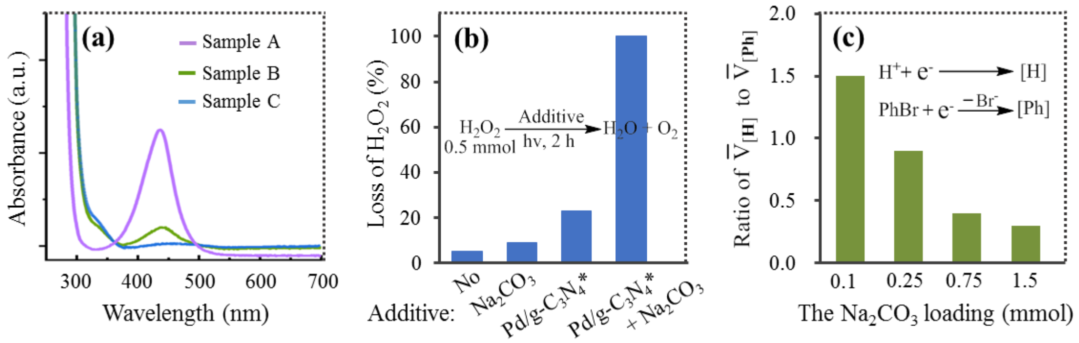

Supplementary Fig. 23 Investigation on the role of Na_2CO_3. (a) UV-Vis spectroscopy related to the produced H_2O_2 in the coupling of bromobenzene. Sample A (purple curve): 0.4 mmol/L aqueous solution of H_2O_2; Sample B (green curve): the produced H_2O_2 under standard conditions (no Na_2CO_3) in Supplementary Table 2; Sample C (blue curve): the produced H_2O_2 under standard conditions in Supplementary Table 2; a.u.: arbitrary units. (b) The H_2O_2 decomposition in the presence of additives (reaction condition: 0.5 mmol H_2O_2, 2 mL H_2O, 2 h, additive, light source (420±10 nm LED, incident light intensity: 0.15 W/cm^2). (c) Effect of the Na_2CO_3 loading on the ratio of $\overline{V}_{[H]}$ to $\overline{V}_{[Ph]}$. PhBr: bromobenzene. [Ph]: intermediate regarding phenyl radical, [H]: intermediate regarding hydrogen radical. $\overline{V}_{[H]}$ and $\overline{V}_{[Ph]}$ are the average rate of the [H] and [Ph] production in 4 h, respectively. $\overline{V}_{[H]}$ and $\overline{V}_{[Ph]}$ was calculated based on the production rate of PhPh, PhH and H_2, see Supplementary Table 9. For the experimental procedure, see Unit 1.13. Source data are provided as a Source Data file.

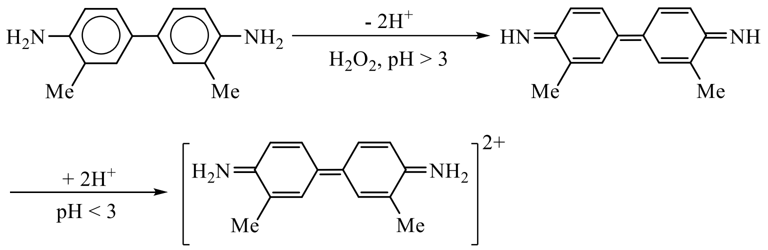

Supplementary Fig. 24 Formation of protonated monomer with absorbance at 438 nm (see Reference 31). Note: o-tolidine is oxidized by H_2O_2 to the dehydrogenated product, which is followed by the protonation of the dehydrogenated product to give the protonated monomer. For the detailed conditions and procedure of the formation of this protonated monomer, see Unit 1.13 in this supplementary information.

a) $\bar{V}_{[H]} = 2\bar{V}_{H_2} + \bar{V}_{PhH}$

b) $\bar{V}_{[Ph]} = 2\bar{V}_{PhPh} + \bar{V}_{PhH}$

c) $\dfrac{\bar{V}_{[H]}}{\bar{V}_{[Ph]}} = \dfrac{2\bar{V}_{H_2} + \bar{V}_{PhH}}{2\bar{V}_{PhPh} + \bar{V}_{PhH}}$

Supplementary Fig. 25 Equations for calculating $\bar{V}_{[H]}$, $\bar{V}_{[Ph]}$ and the ratio of $\bar{V}_{[H]}$ to $\bar{V}_{[Ph]}$. (a) Equations for calculating $\bar{V}_{[H]}$. $\bar{V}_{[H]}$: the average rate of the [H] production in 2 h, $\bar{V}_{H2:}$ the average rate of the H_2 production in 2 h, $\bar{V}_{PhH}$: the average rate of the benzene production in 2 h. (b) Equations for calculating $\bar{V}_{[Ph]}$. $\bar{V}_{[Ph]}$: the average rate of the [Ph] production in 2 h, $\bar{V}_{PhPh}$: the average rate of the biphenyl production in 2 h. (c) Equations for calculating the ratio of $\bar{V}_{[H]}$ to $\bar{V}_{[Ph]}$. These equations can be deduced based on Supplementary Fig. 26. [Ph]: intermediate regarding phenyl radical, [H]: intermediate regarding hydrogen radical.

$$\mathrm{PhBr} + e^- \xrightarrow{-\mathrm{Br}^-} [\mathrm{Ph}] \longrightarrow 0.5\mathrm{PhPh}$$

$$\mathrm{H}^+ + e^- \longrightarrow [\mathrm{H}] \longrightarrow 0.5\mathrm{H}_2$$

$$[\mathrm{Ph}] + [\mathrm{H}] \longrightarrow \mathrm{PhH}$$

Supplementary Fig. 26 Products from reductive half reactions under our conditions. PhBr: bromobenzene, PhPh: biphenyl, PhH: benzene, [Ph]: intermediate regarding phenyl radical, [H]: intermediate regarding hydrogen radical.

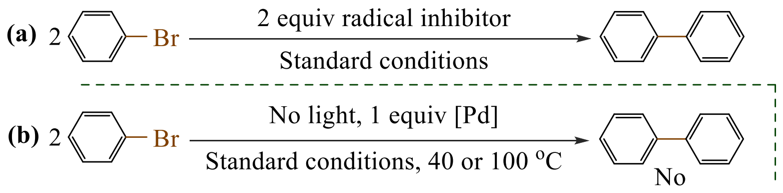

Radical inhibitor	Yield
no	96%
BHT	15%
TEMPO	No

Supplementary Fig. 27 Investigation on whether or not the reaction undergoes the SeT. (a) Effect of radical inhibitors on the reaction. TEMPO: 2,2,6,6-tetramethylpiperidine-1-oxyl, BHT: 2,6-di^{t}butyl-4-methylphenol. (b) Reaction results in the case of no illumination and 1 equiv Pd^0 species. SeT: single electron transfer. [Pd]: Pd^0 species in Pd/g-C_3N_4*. Standard conditions: 0.5 mmol bromobenzene, 15 mg Pd/g-C_3N_4* (2.8 wt%), 1.5 equiv Na_2CO_3, 5 mL H_2O, 3 mL 1,4-dioxane, 20 h, Ar atmosphere, light source: 420±10 nm LED, incident light intensity: 0.15 W/cm^2. For the experimental procedure, see Unit 1.14.

(a) One-step process:

$$2H_2O \xrightarrow{-4e} O_2 + 4H^+$$

(b) Two-step process:

$$2H_2O \xrightarrow{-2e} H_2O_2 + 2H^+ \xrightarrow{-2e} O_2 + 4H^+$$

Supplementary Fig. 28 Two pathways for the water oxidation. (a) Four-electron one-step process for the water oxidation to O_2. Note: this pathway does not include the formation of H_2O_2. (b) $2e^-/2e^-$ two-step process for the water oxidation to O_2. Note: this pathway includes the formation of H_2O_2.

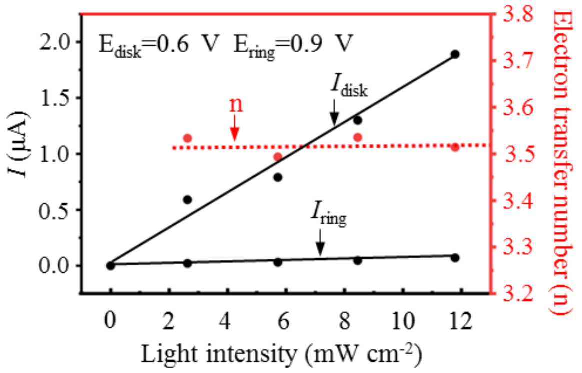

Supplementary Fig. 29 Results from rotating disk-ring electrode (RRDE) experiments. E_{disk}: voltage of disk, E_{ring}: voltage of ring, I_{disk}: current of disk, I_{ring}: current of ring. For the experimental procedure, see Unit 1.14. Source data are provided as a Source Data file.

6. Copies of product NMR spectra

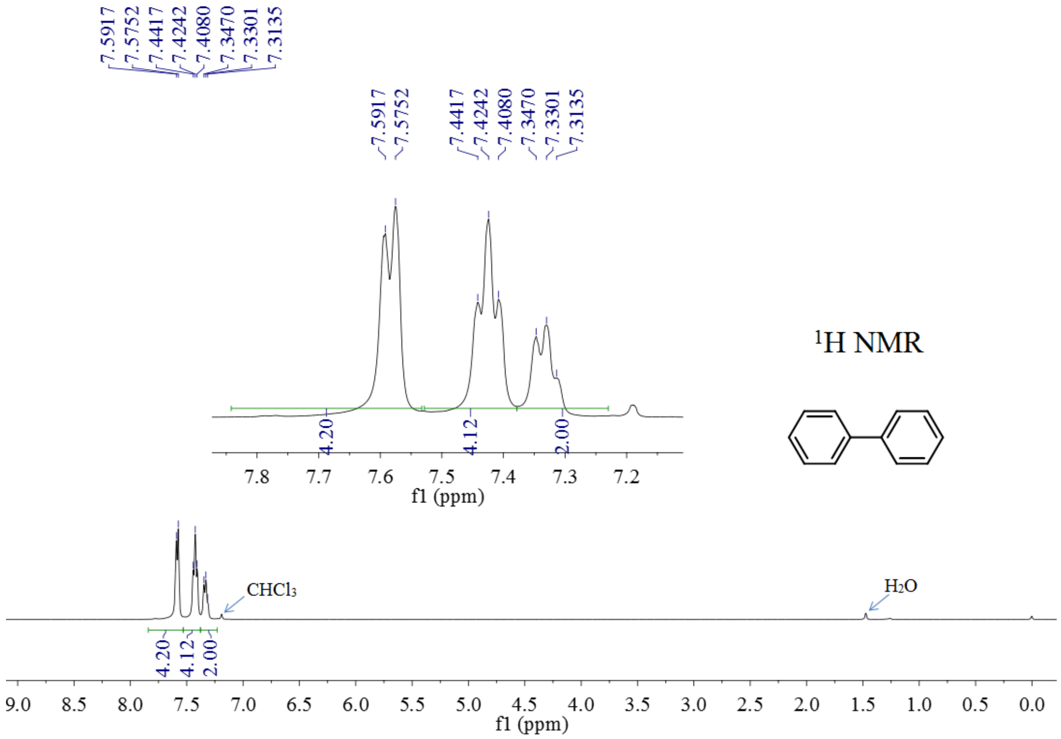

Supplementary Fig. 30: ^{1}H NMR spectrum of biphenyl. Note: this is a known compound.[21] Measurement conditions: 400 MHz, $CDCl_3$, room temperature.

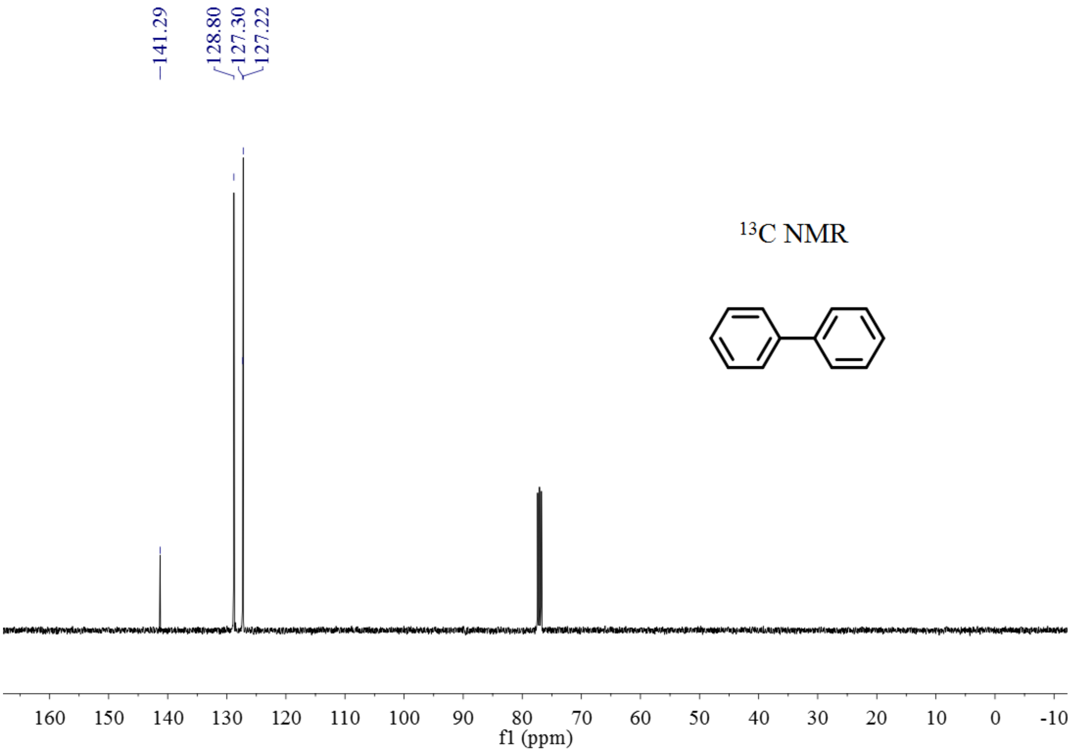

Supplementary Fig. 31: ^{13}C NMR spectrum of biphenyl. Note: this is a known compound.[21] Measurement conditions: 101 MHz, $CDCl_3$, room temperature.

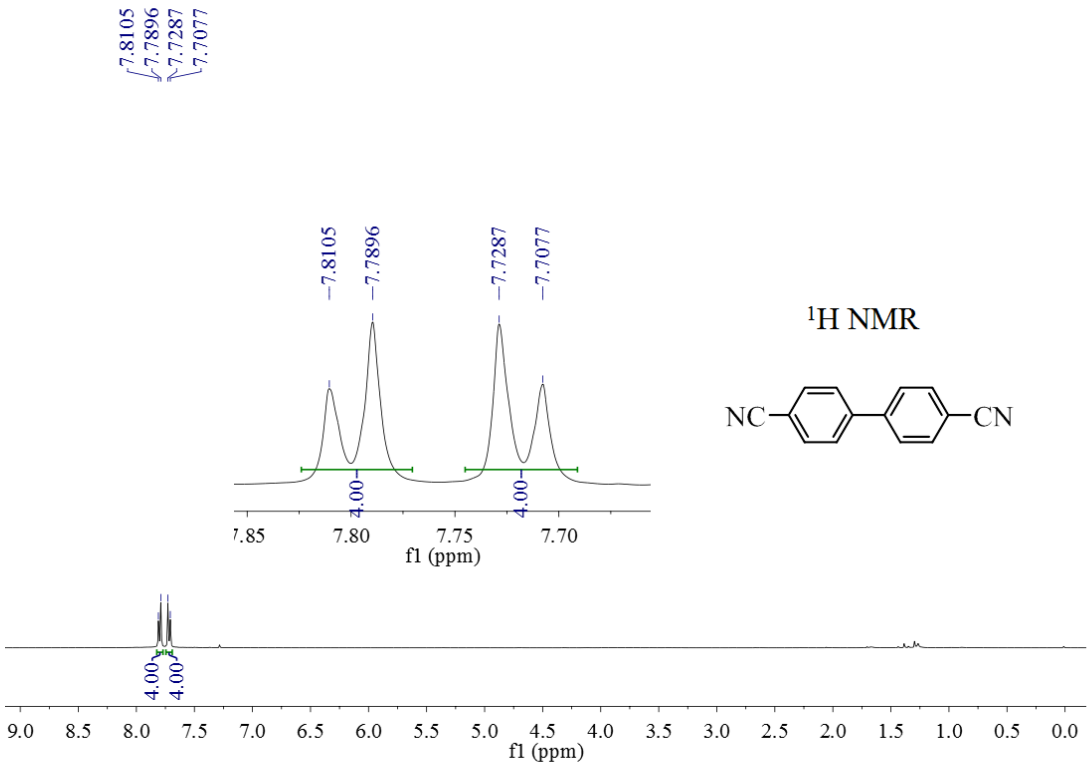

Supplementary Fig. 32: ^{1}H NMR spectrum of 4,4'-dicarbonitrilebiphenyl. Note: this is a known compound.[21] Measurement conditions: 400 MHz, $CDCl_3$, room temperature.

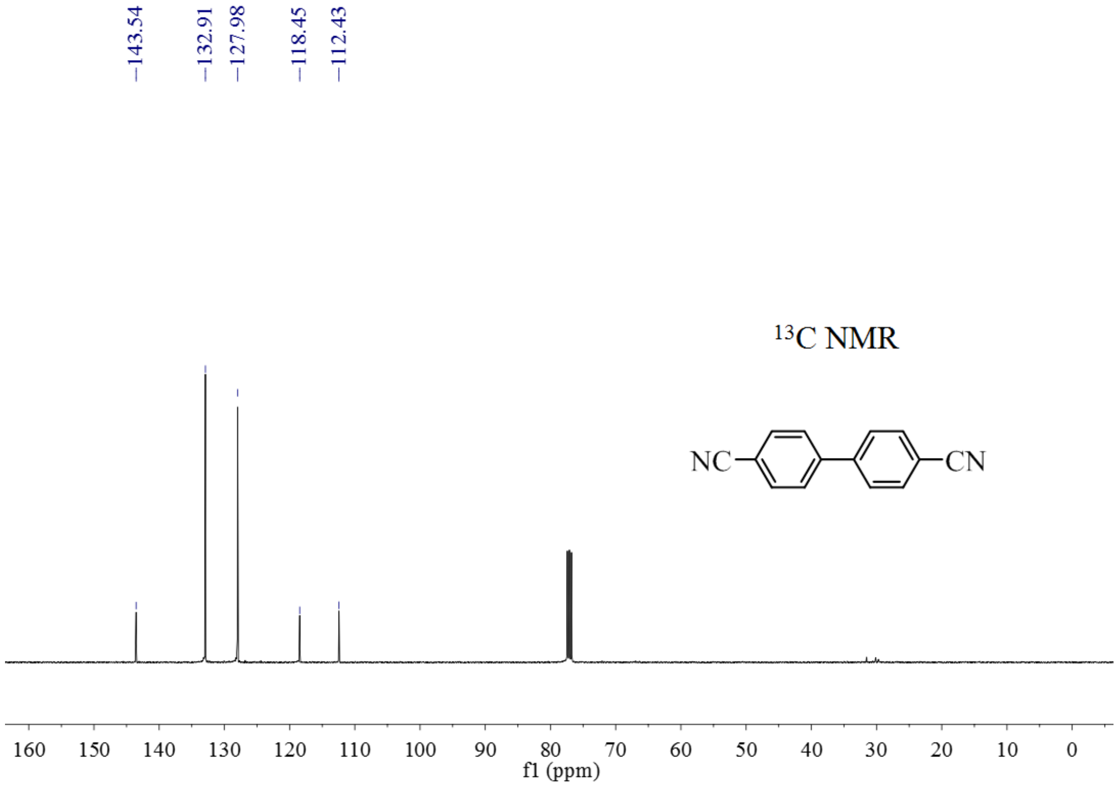

Supplementary Fig. 33: ^{13}C NMR spectrum of 4,4'-dicarbonitrilebiphenyl. Note: this is a known compound.[21] Measurement conditions: 101 MHz, $CDCl_3$, room temperature.

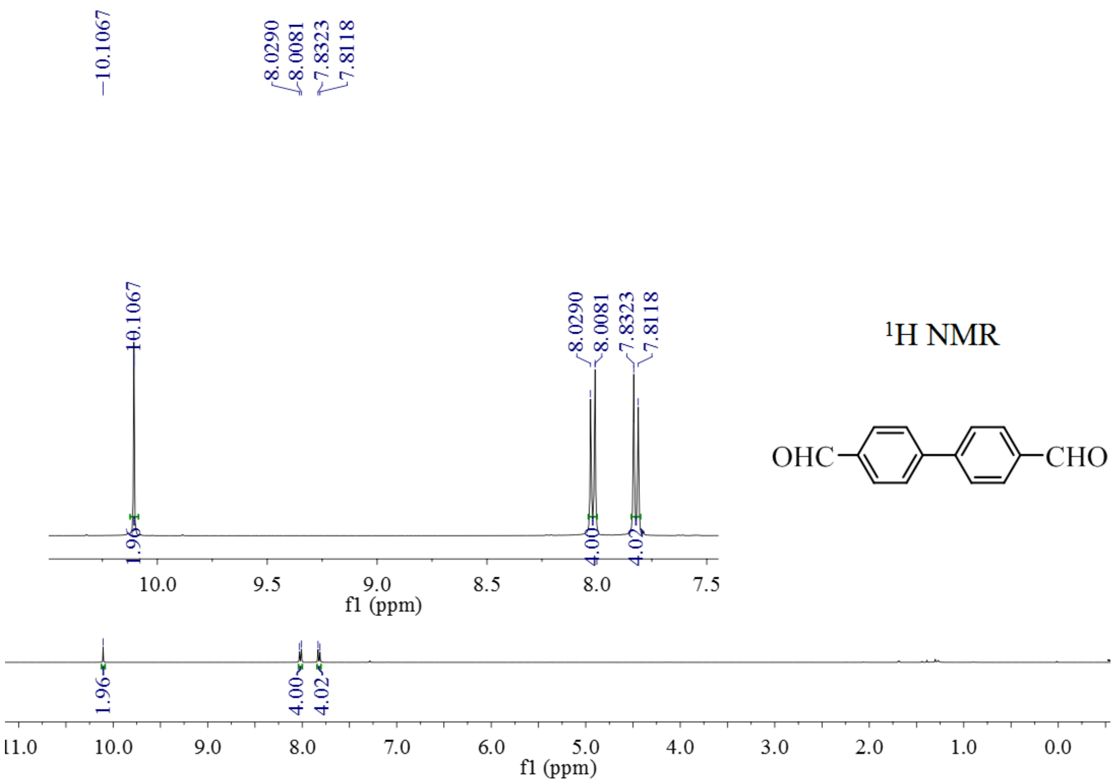

Supplementary Fig. 34: ^{1}H NMR spectrum of 4,4'-diformylbiphenyl. Note: this is a known compound.[21] Measurement conditions: 400 MHz, $CDCl_3$, room temperature.

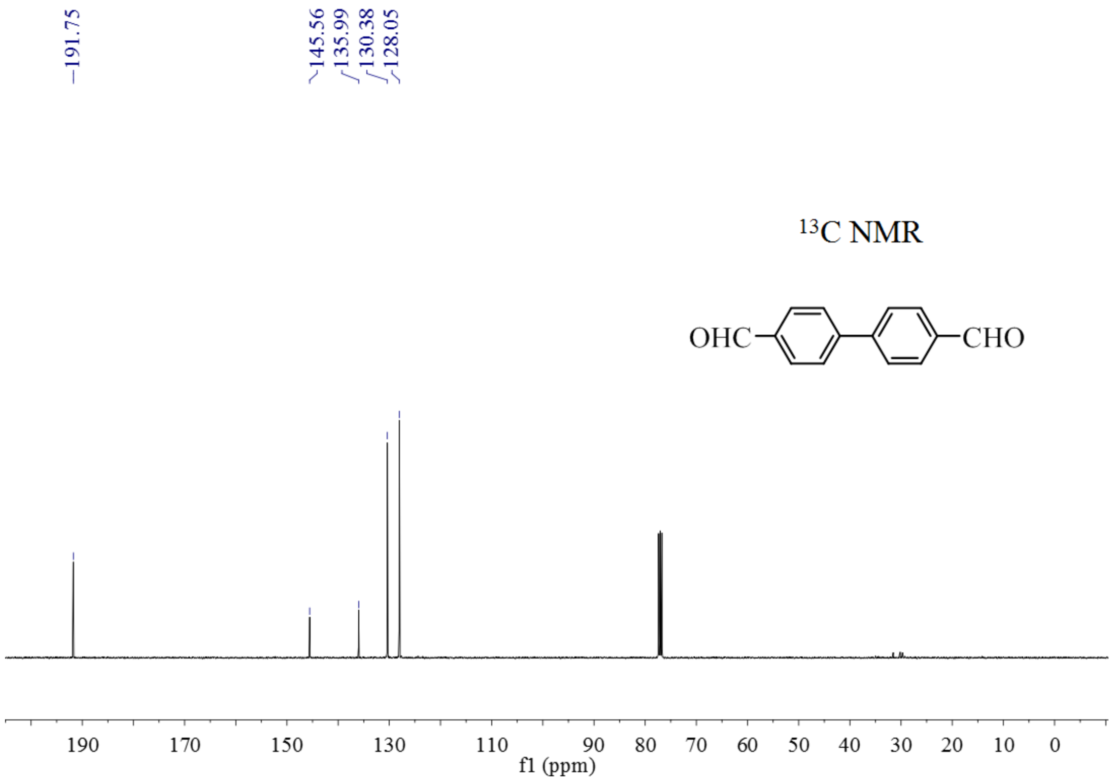

Supplementary Fig. 35: ^{13}C NMR spectrum of 4,4'-diformylbiphenyl. Note: this is a known compound.[21] Measurement conditions: 101 MHz, $CDCl_3$, room temperature.

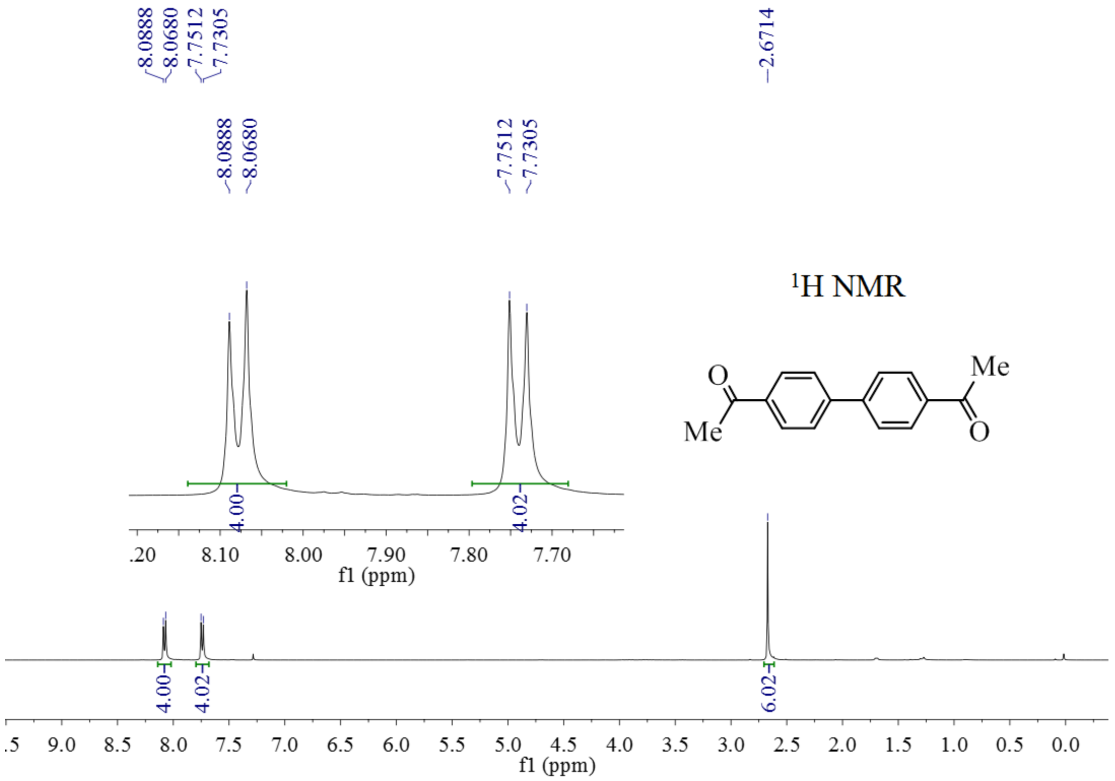

Supplementary Fig. 36: ^{1}H NMR spectrum of 4,4'-diacetylbiphenyl. Note: this is a known compound.[21] Measurement conditions: 400 MHz, $CDCl_3$, room temperature.

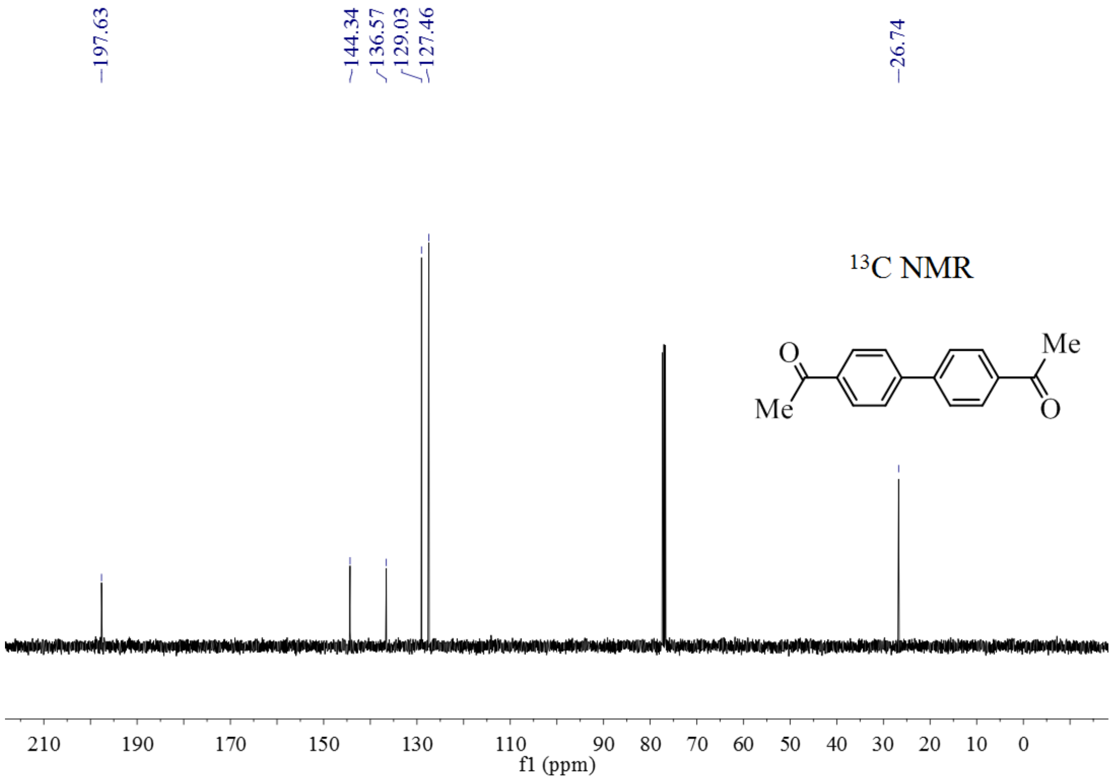

Supplementary Fig. 37: ^{13}C NMR spectrum of 4,4'-diacetylbiphenyl. Note: this is a known compound.[21] Measurement conditions: 101 MHz, $CDCl_3$, room temperature.

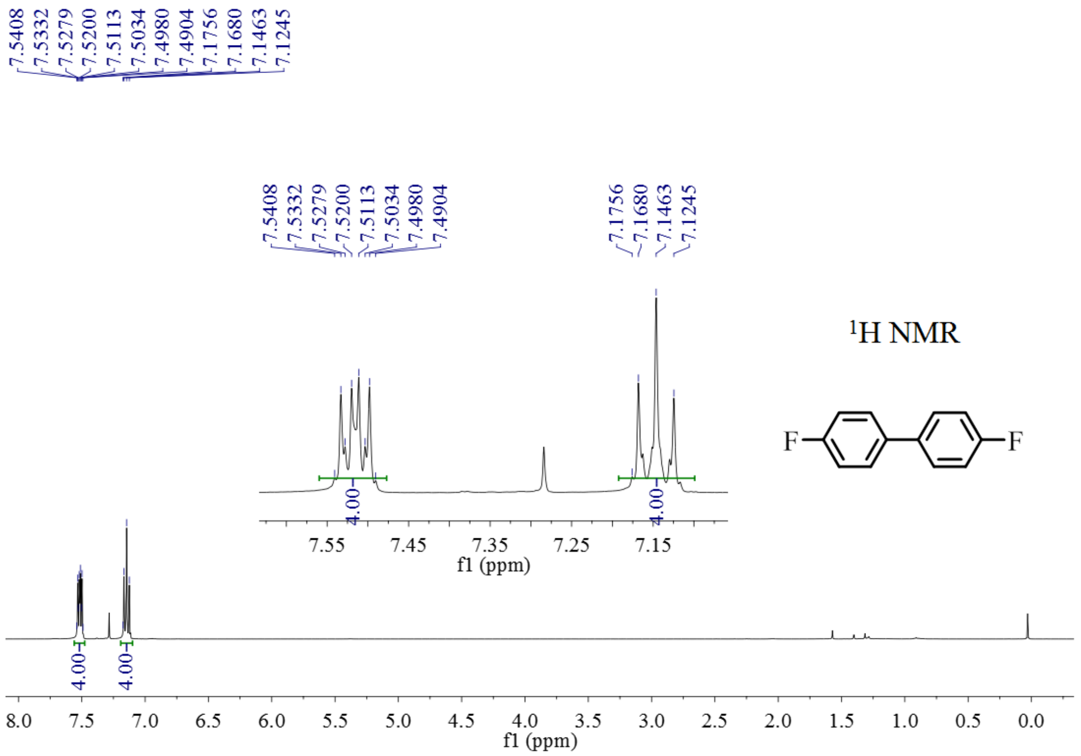

Supplementary Fig. 38: ^{1}H NMR spectrum of 4,4'-difluorobiphenyl. Note: this is a known compound.[21] Measurement conditions: 400 MHz, $CDCl_3$, room temperature.

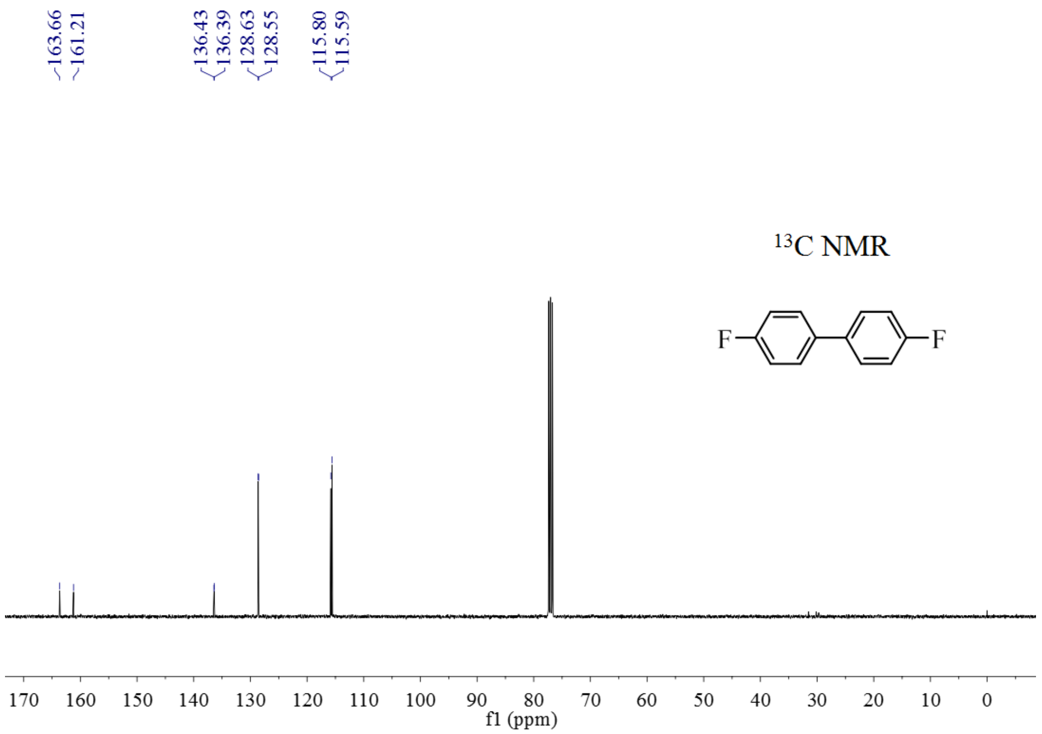

Supplementary Fig. 39: ^{13}C NMR spectrum of 4,4'-difluorobiphenyl. Note: this is a known compound.[21] Measurement conditions: 101 MHz, $CDCl_3$, room temperature.

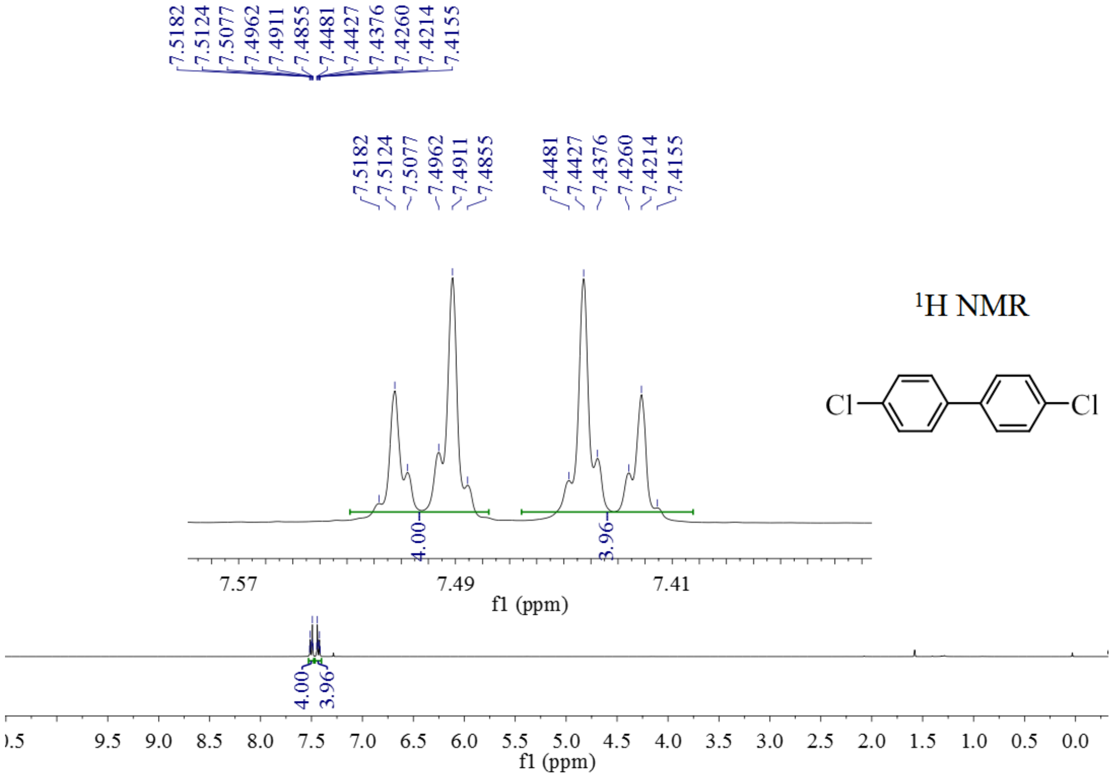

Supplementary Fig. 40: ^{1}H NMR spectrum of 4,4'-dichlorobiphenyl. Note: this is a known compound.[21] Measurement conditions: 400 MHz, $CDCl_3$, room temperature.

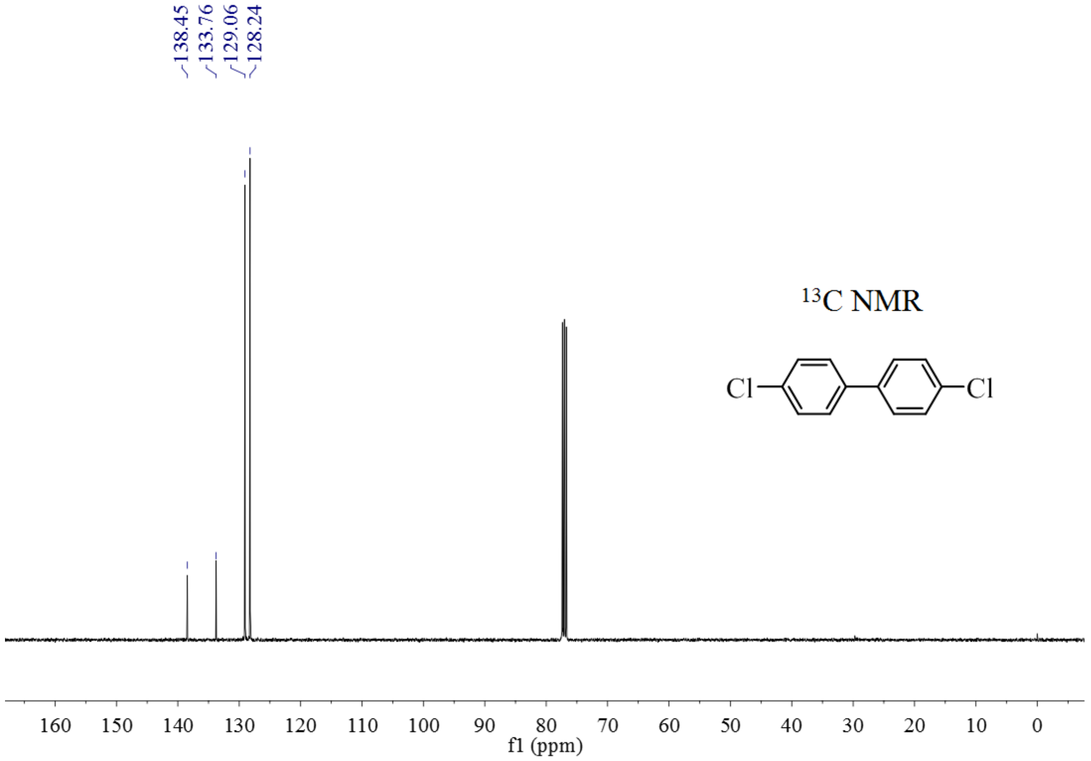

Supplementary Fig. 41: ^{13}C NMR spectrum of 4,4'-dichlorobiphenyl. Note: this is a known compound.[21] Measurement conditions: 101 MHz, $CDCl_3$, room temperature.

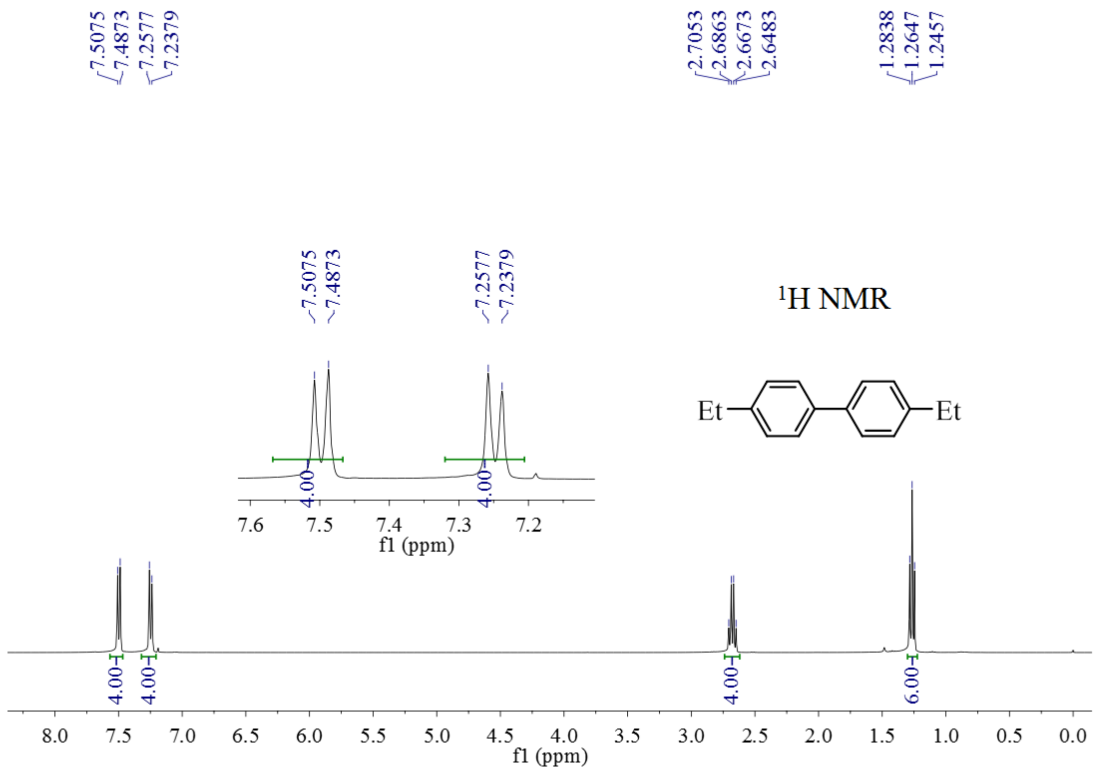

Supplementary Fig. 42: ^{1}H NMR spectrum of 4,4'-diethylbiphenyl. Note: this is a known compound.[22] Measurement conditions: 400 MHz, $CDCl_3$, room temperature.

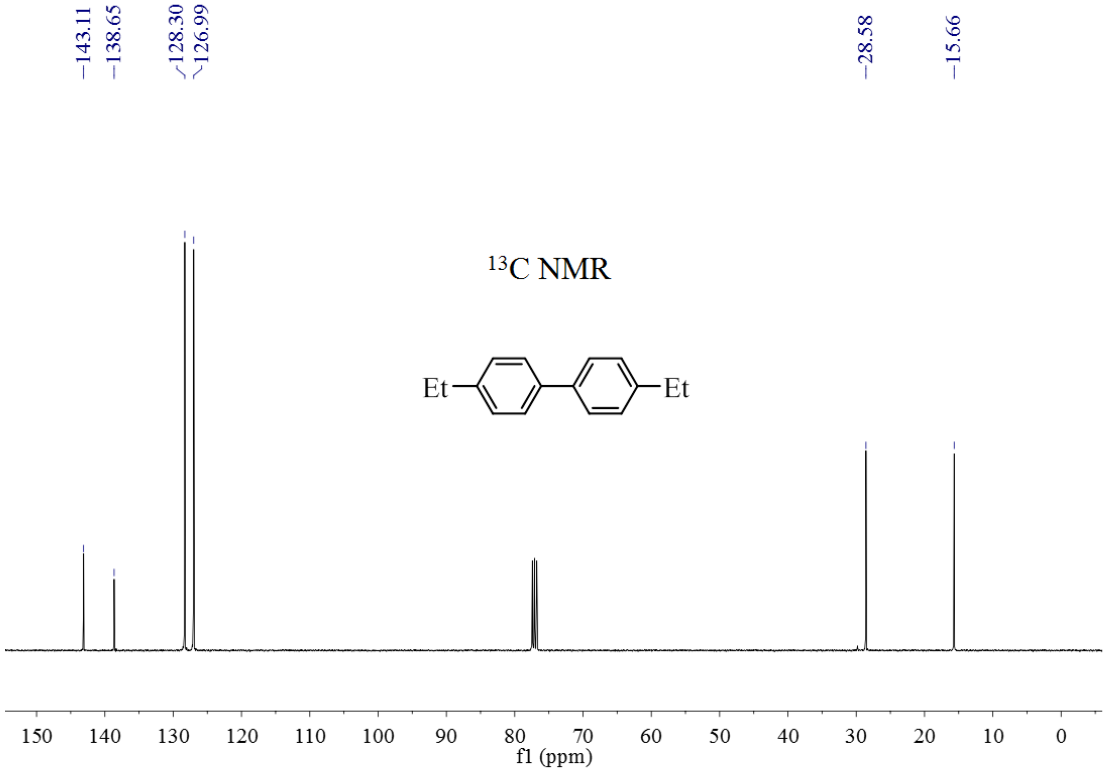

Supplementary Fig. 43: ^{13}C NMR spectrum of 4,4'-diethylbiphenyl. Note: this is a known compound.[22] Measurement conditions: 101 MHz, $CDCl_3$, room temperature.

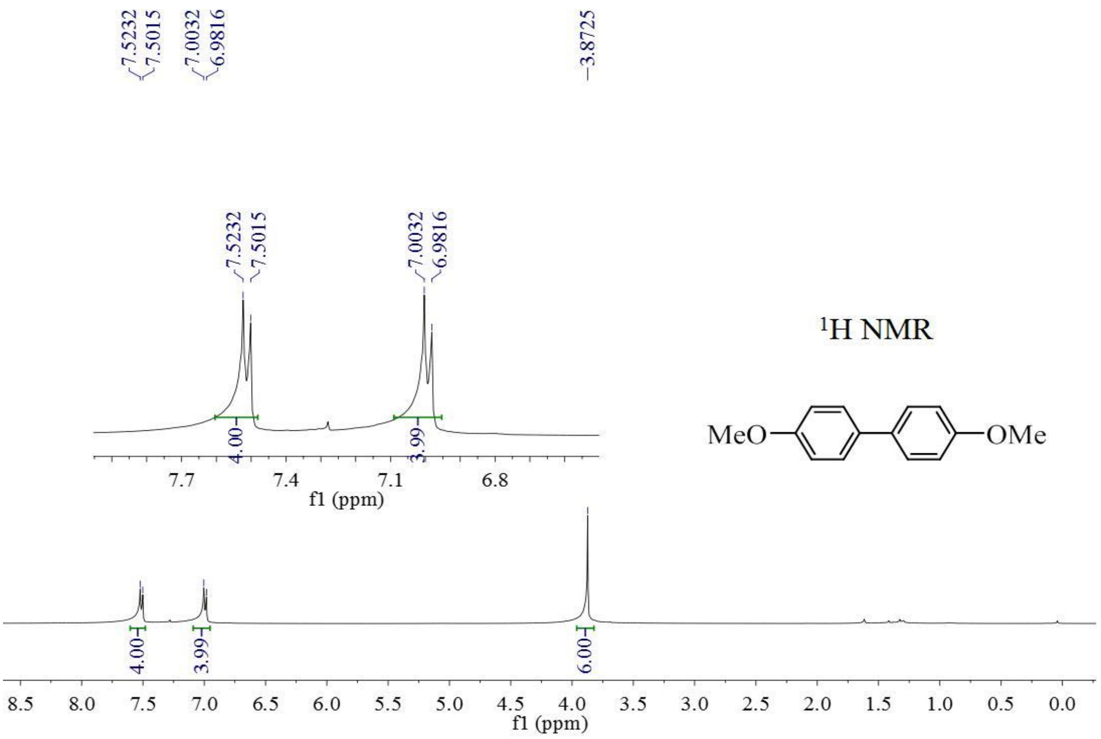

Supplementary Fig. 44: ^{1}H NMR spectrum of 4,4'-dimethoxylbiphenyl. Note: this is a known compound.[21] Measurement conditions: 400 MHz, $CDCl_3$, room temperature.

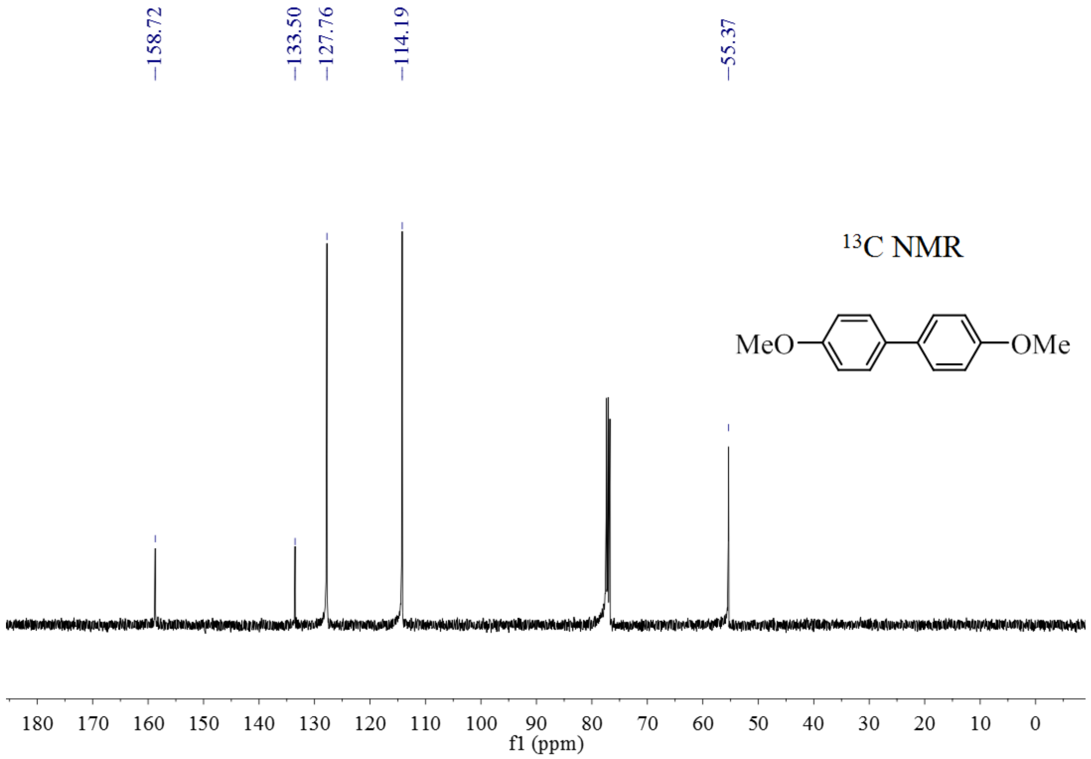

Supplementary Fig. 45: ^{13}C NMR spectrum of 4,4'-dimethoxylbiphenyl. Note: this is a known compound.[21] Measurement conditions: 101 MHz, $CDCl_3$, room temperature.

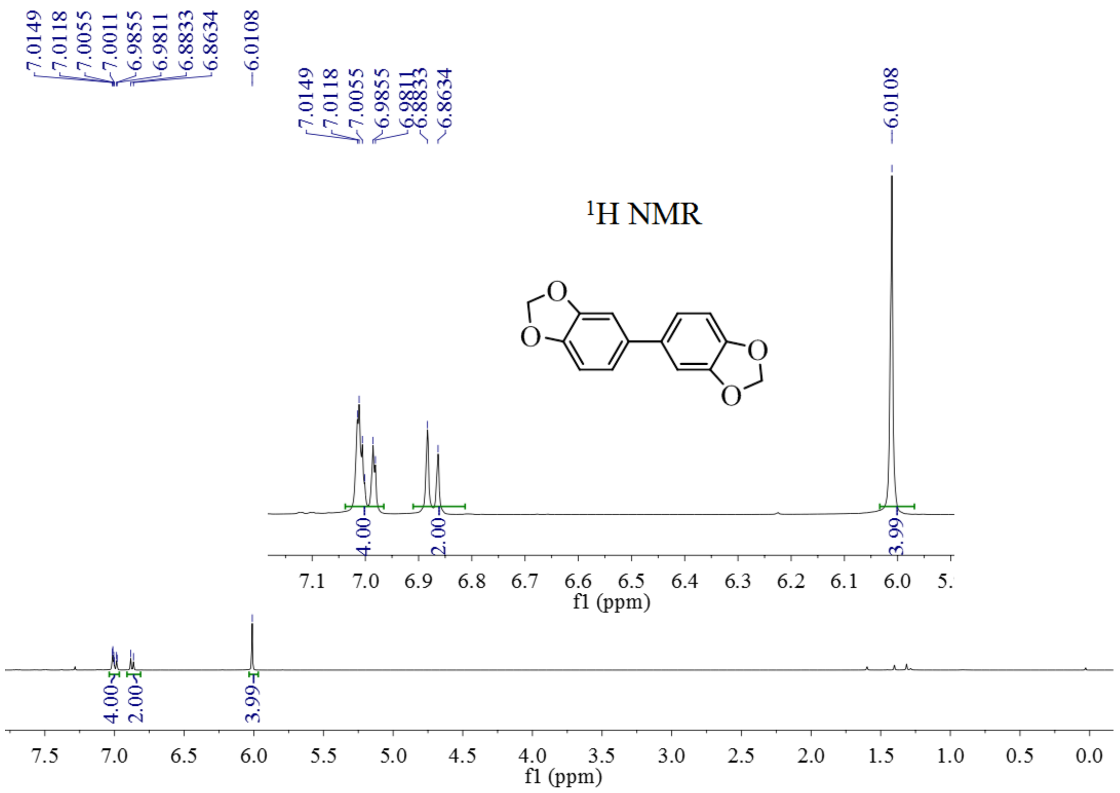

Supplementary Fig. 46: ^{1}H NMR spectrum of 3,3',4,4'-bis(methylenedioxy)biphenyl. Note: this is a known compound.[23] Measurement conditions: 400 MHz, $CDCl_3$, room temperature.

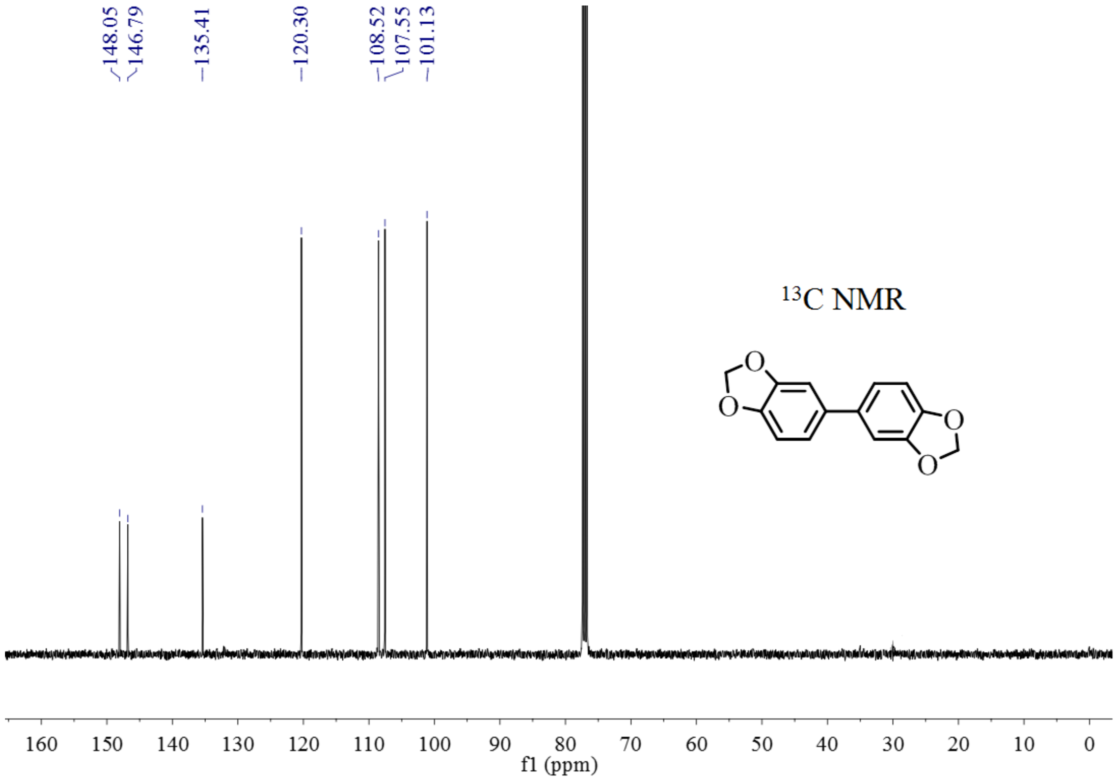

Supplementary Fig. 47: ^{13}C NMR spectrum of 3,3',4,4'-bis(methylenedioxy)biphenyl. Note: this is a known compound.[23] Measurement conditions: 101 MHz, $CDCl_3$, room temperature.

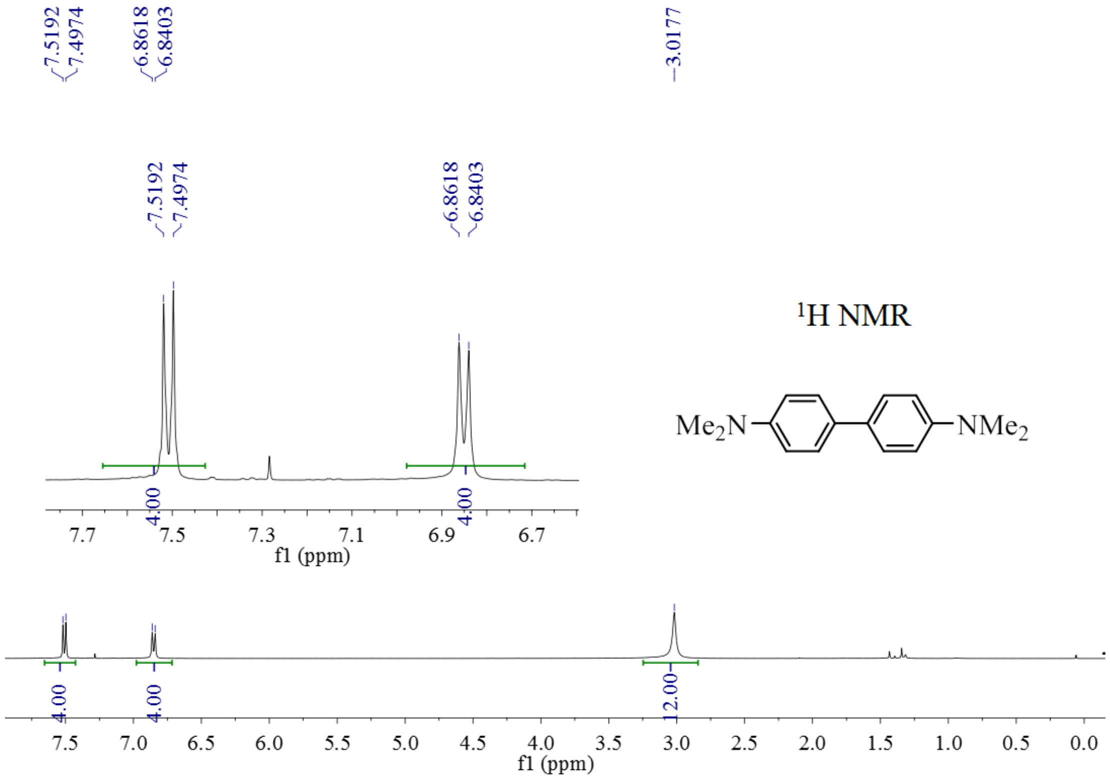

Supplementary Fig. 48: ^{1}H NMR spectrum of 4,4'-bis(dimethylamino)biphenyl. Note: this is a known compound.[24] Measurement conditions: 400 MHz, $CDCl_3$, room temperature.

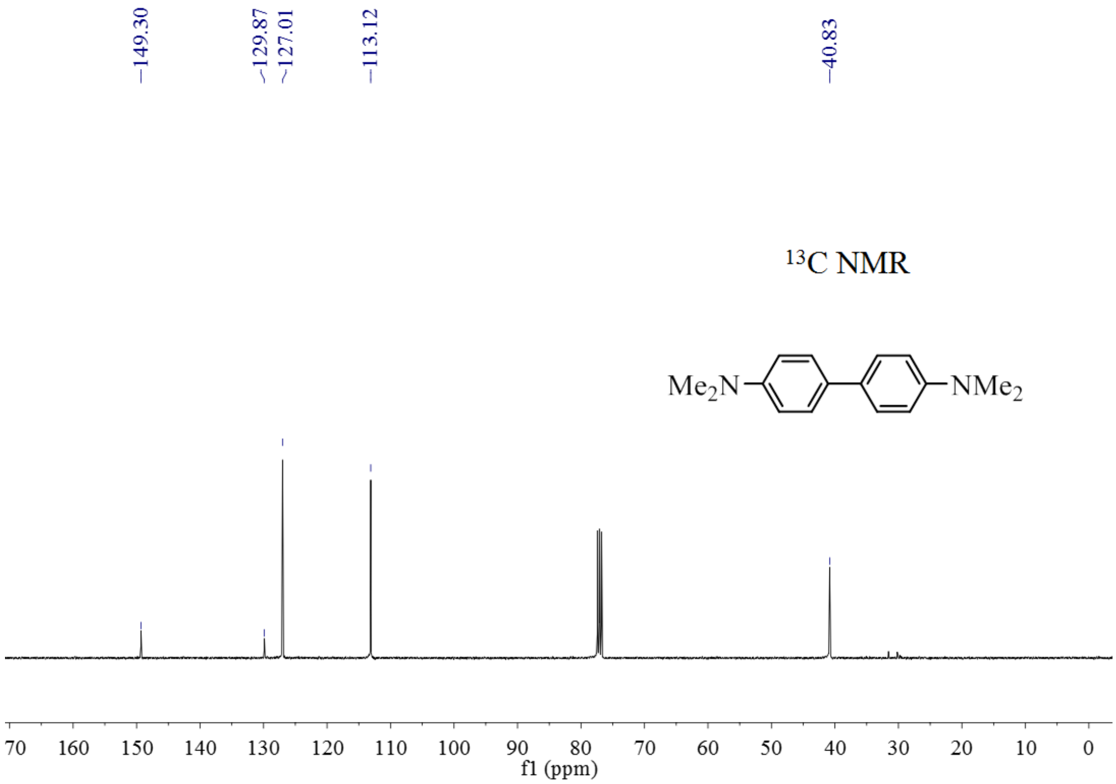

Supplementary Fig. 49: ^{13}C NMR spectrum of 4,4'-bis(dimethylamino)biphenyl. Note: this is a known compound.[24] Measurement conditions: 101 MHz, $CDCl_3$, room temperature.

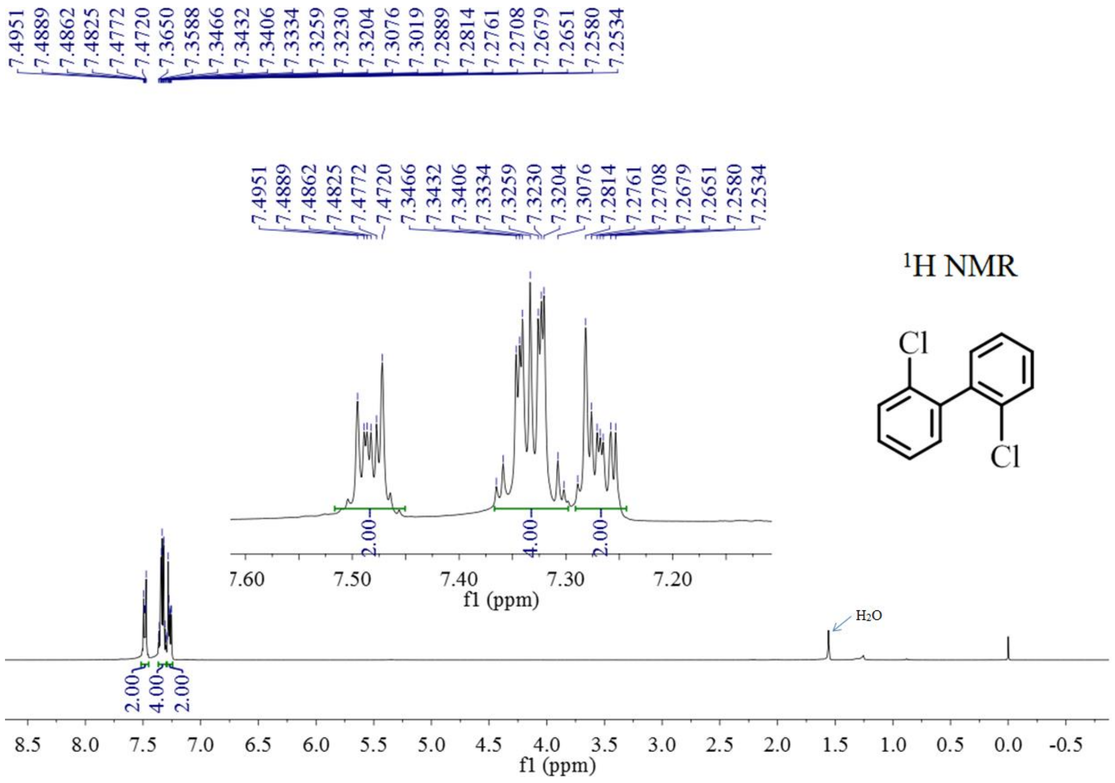

Supplementary Fig. 50: ^{1}H NMR spectrum of 2,2'-dichlorobiphenyl. Note: this is a known compound.[21] Measurement conditions: 400 MHz, $CDCl_3$, room temperature.

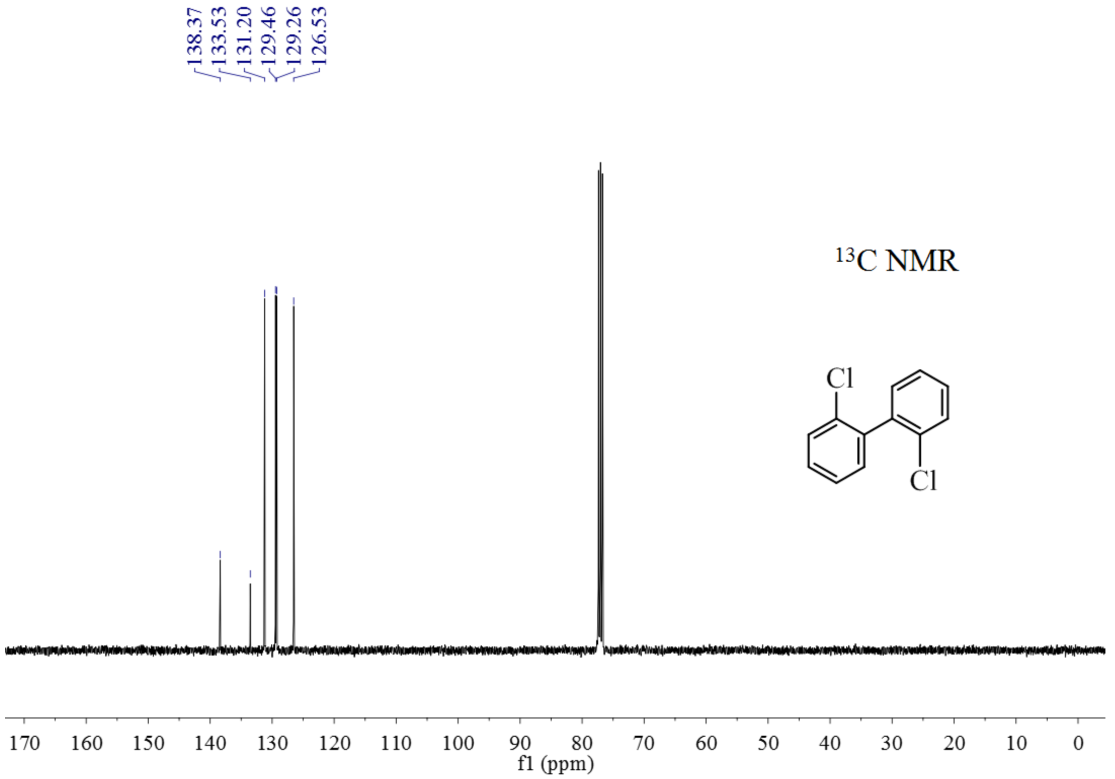

Supplementary Fig. 51: ^{13}C NMR spectrum of 2,2'-dichlorobiphenyl. Note: this is a known compound.[21] Measurement conditions: 101 MHz, $CDCl_3$, room temperature.

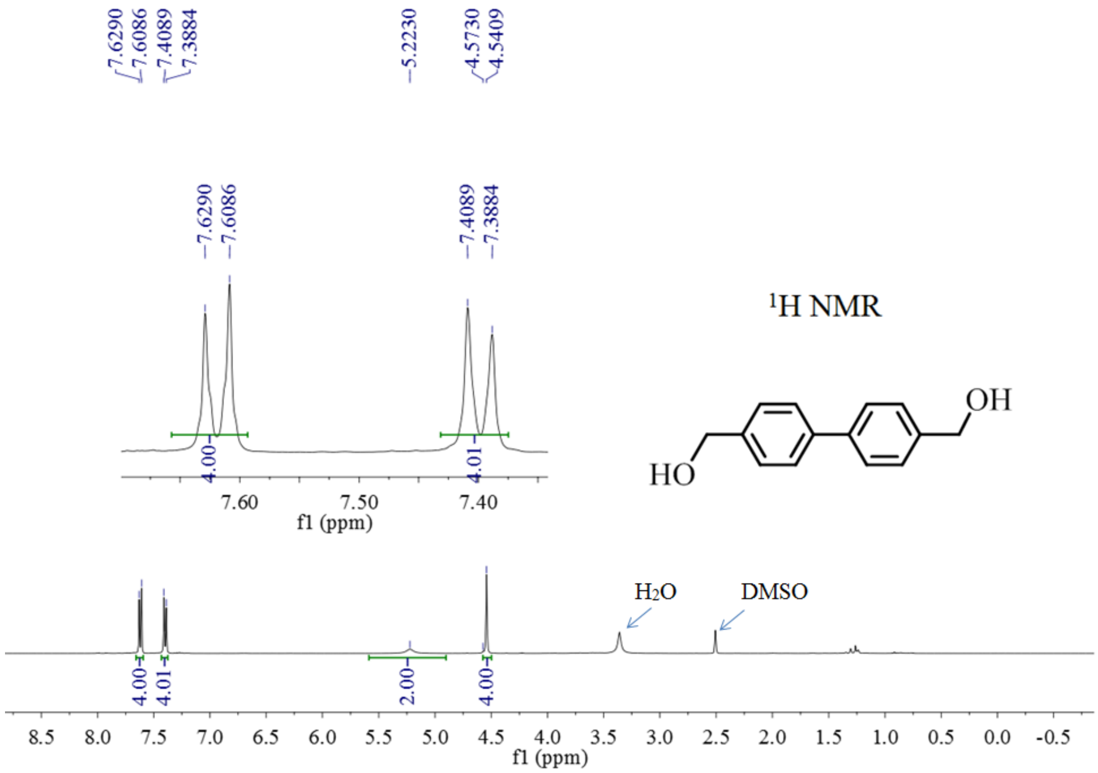

Supplementary Fig. 52: ^{1}H NMR spectrum of 4,4'-diyldimethanolbiphenyl. Note: this is a known compound.[26] Measurement conditions: 400 MHz, DMSO-d_6, room temperature.

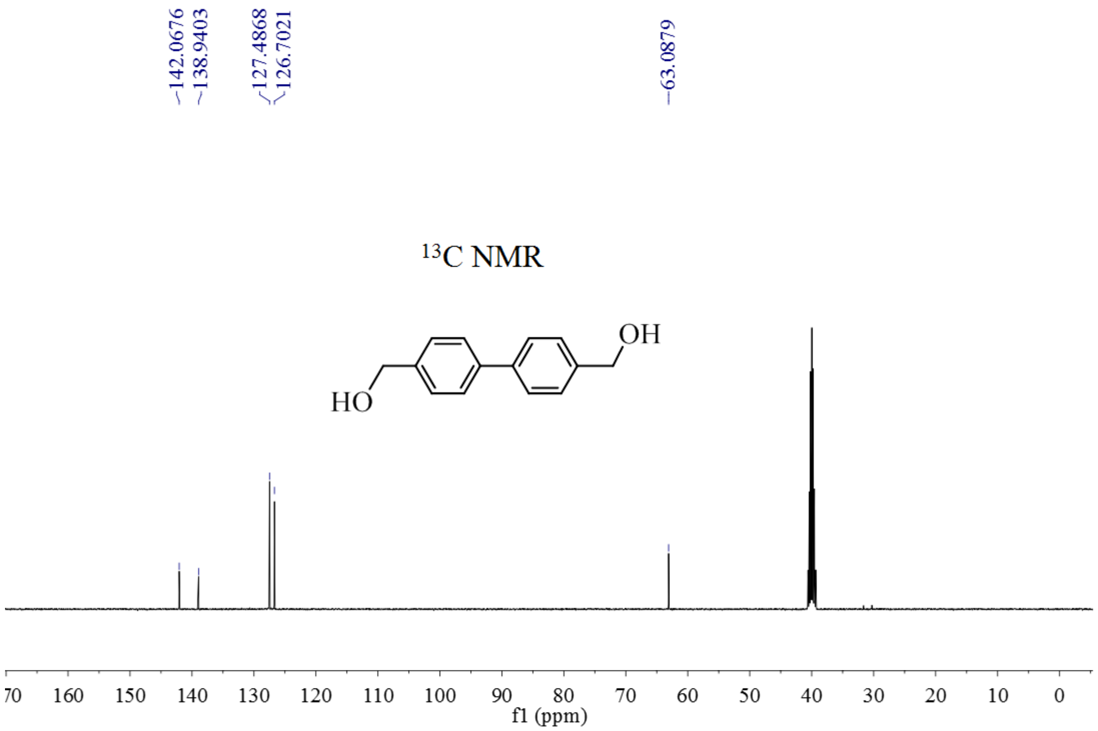

Supplementary Fig. 53: ^{13}C NMR spectrum of 4,4'-diyldimethanolbiphenyl. Note: this is a known compound.[26] Measurement conditions: 101 MHz, DMSO-d_6, room temperature.

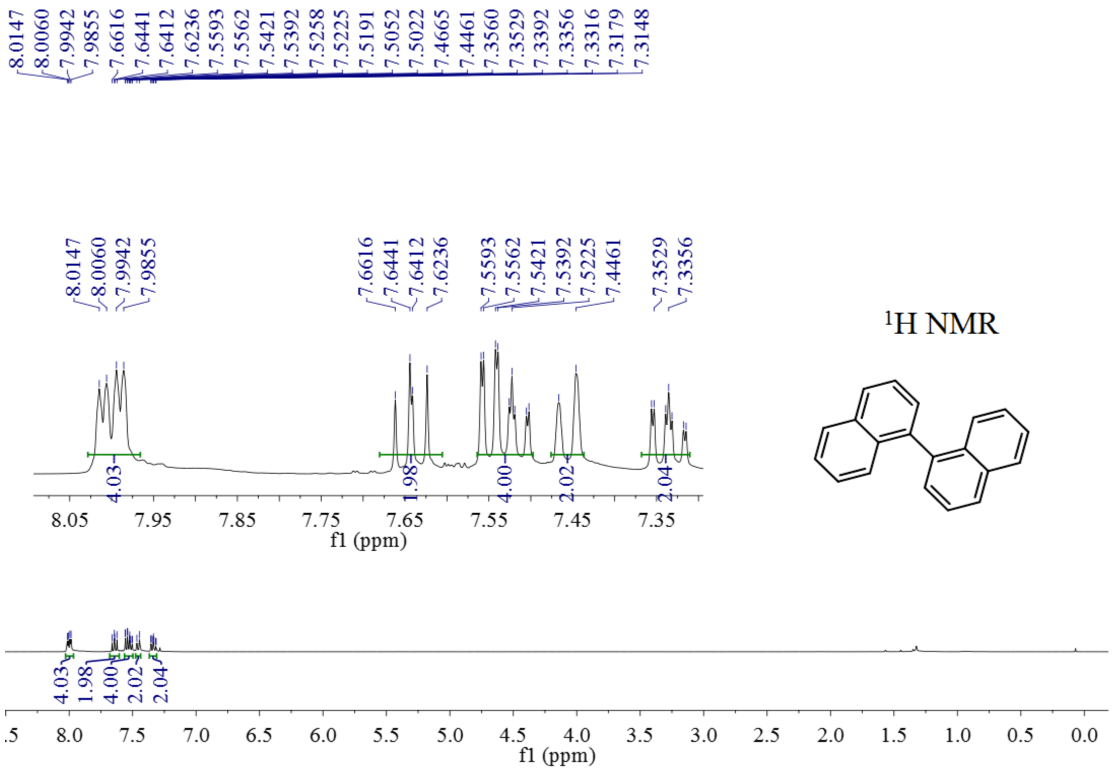

Supplementary Fig. 54: ^{1}H NMR spectrum of 1,1'-binaphthalene. Note: this is a known compound.[21] Measurement conditions: 400 MHz, $CDCl_3$, room temperature.

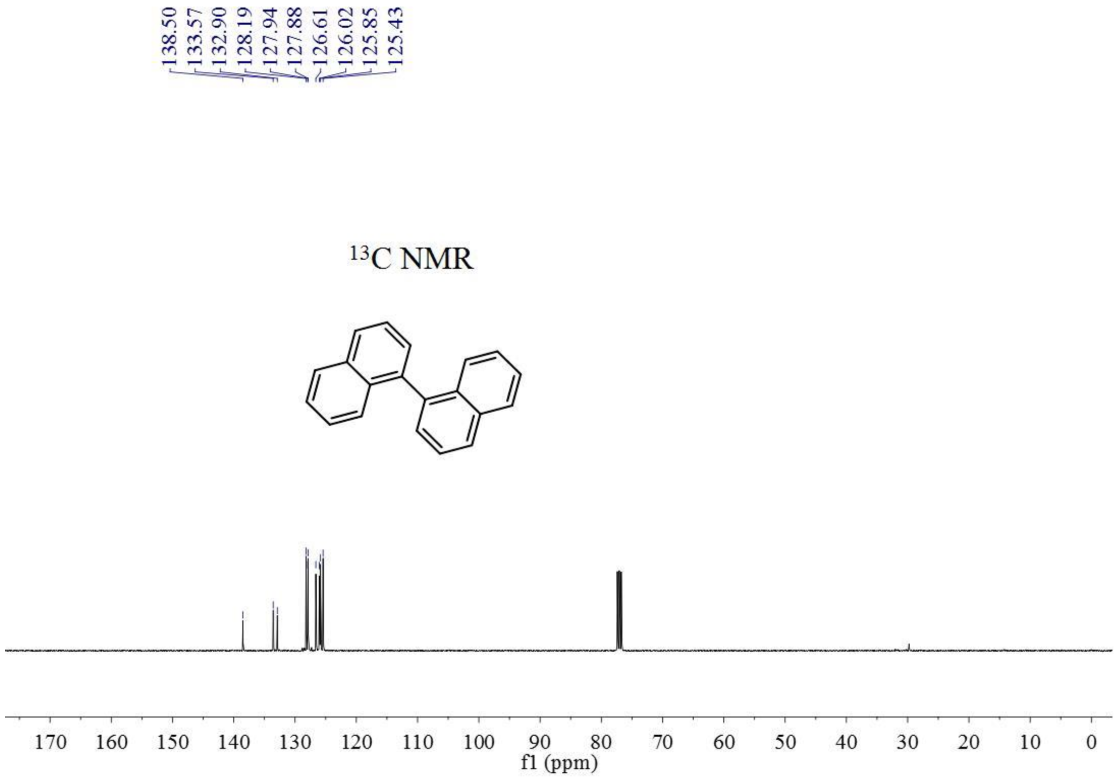

Supplementary Fig. 55: ^{13}C NMR spectrum of 1,1'-binaphthalene. Note: this is a known compound.[21] Measurement conditions: 101 MHz, $CDCl_3$, room temperature.

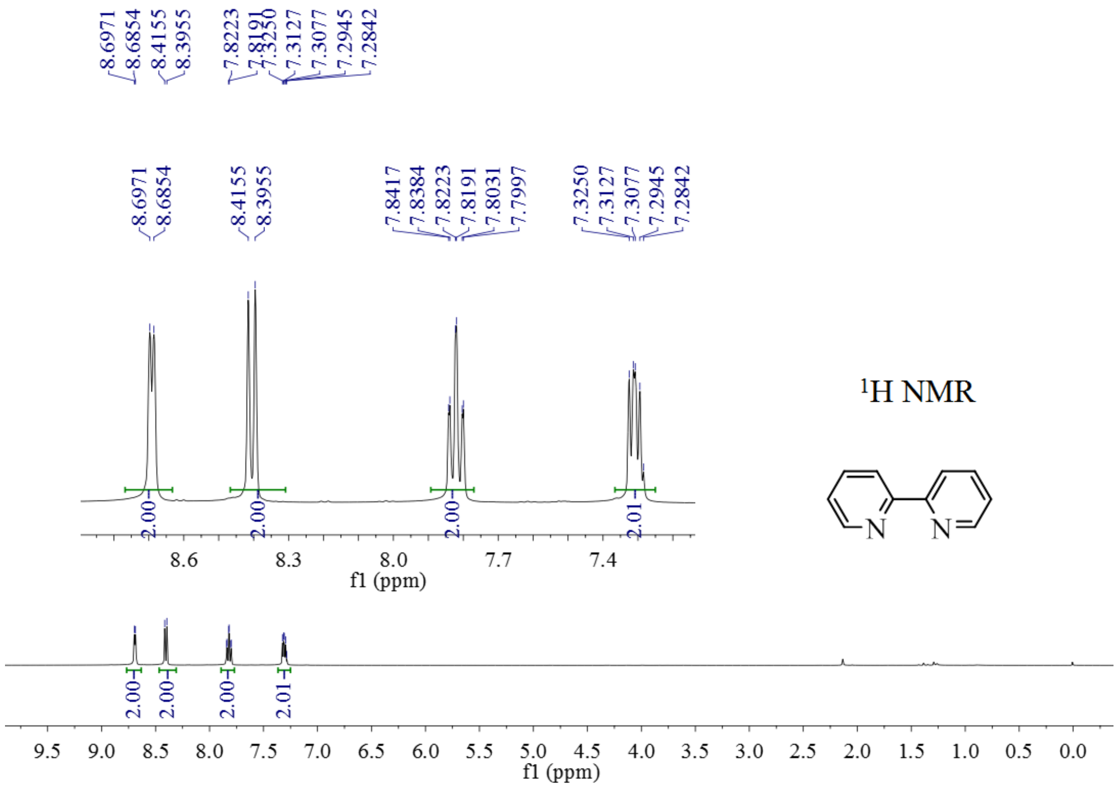

Supplementary Fig. 56: ¹H NMR spectrum of 2,2'-bipyridine. Note: this is a known compound.[24] Measurement conditions: 400 MHz, $CDCl_3$, room temperature.

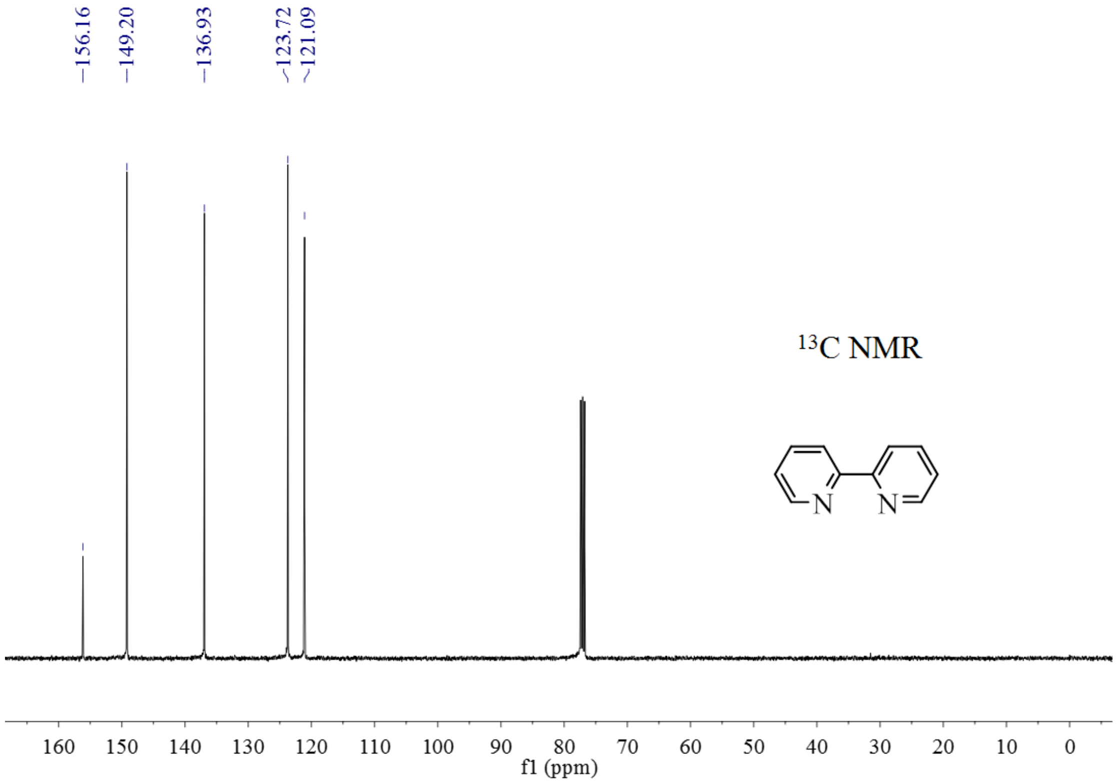

Supplementary Fig. 57: ¹³C NMR spectrum of 2,2'-bipyridine. Note: this is a known compound.[24] Measurement conditions: 101 MHz, $CDCl_3$, room temperature.

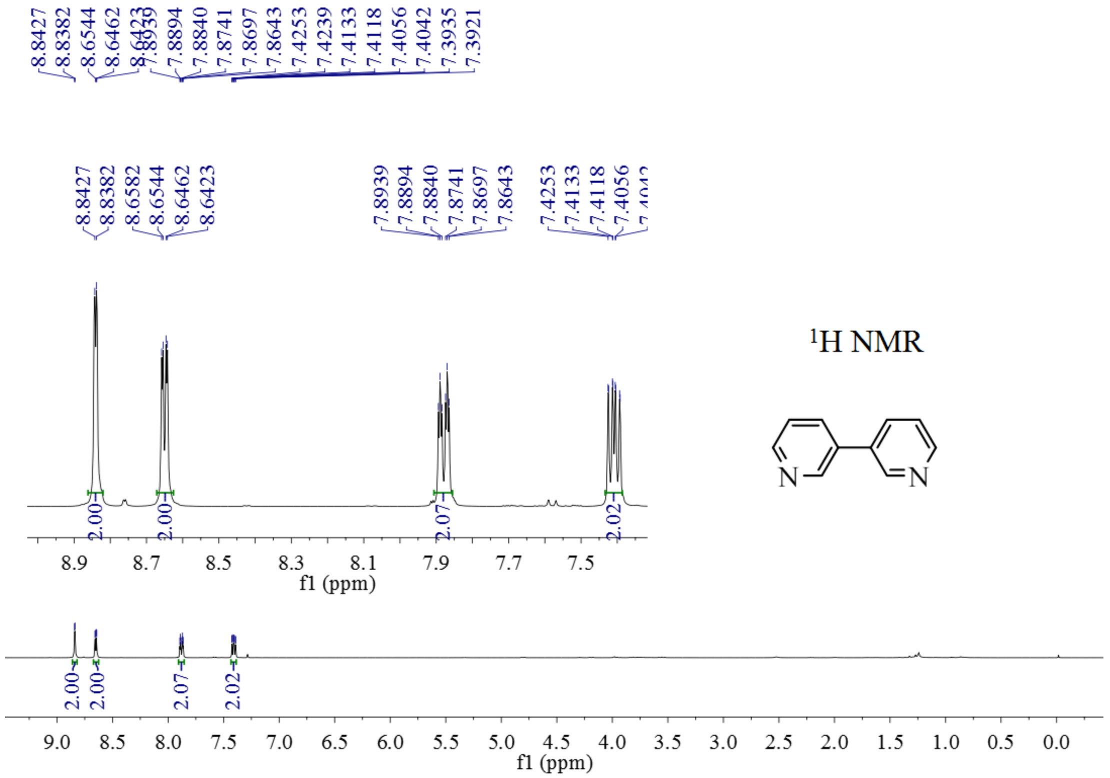

Supplementary Fig. 58: ^{1}H NMR spectrum of 3,3'-bipyridine. Note: this is a known compound.[24] Measurement conditions: 400 MHz, $CDCl_3$, room temperature.

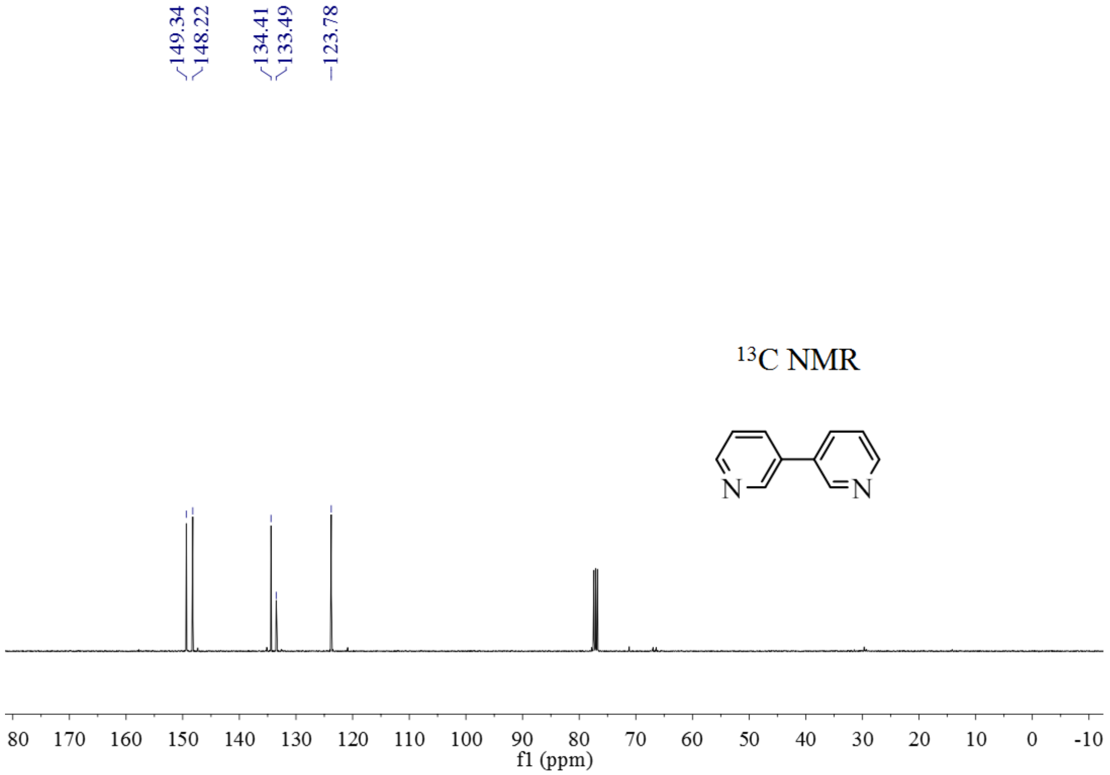

Supplementary Fig. 59: ^{13}C NMR spectrum of 3,3'-bipyridine. Note: this is a known compound.[24] Measurement conditions: 101 MHz, $CDCl_3$, room temperature.

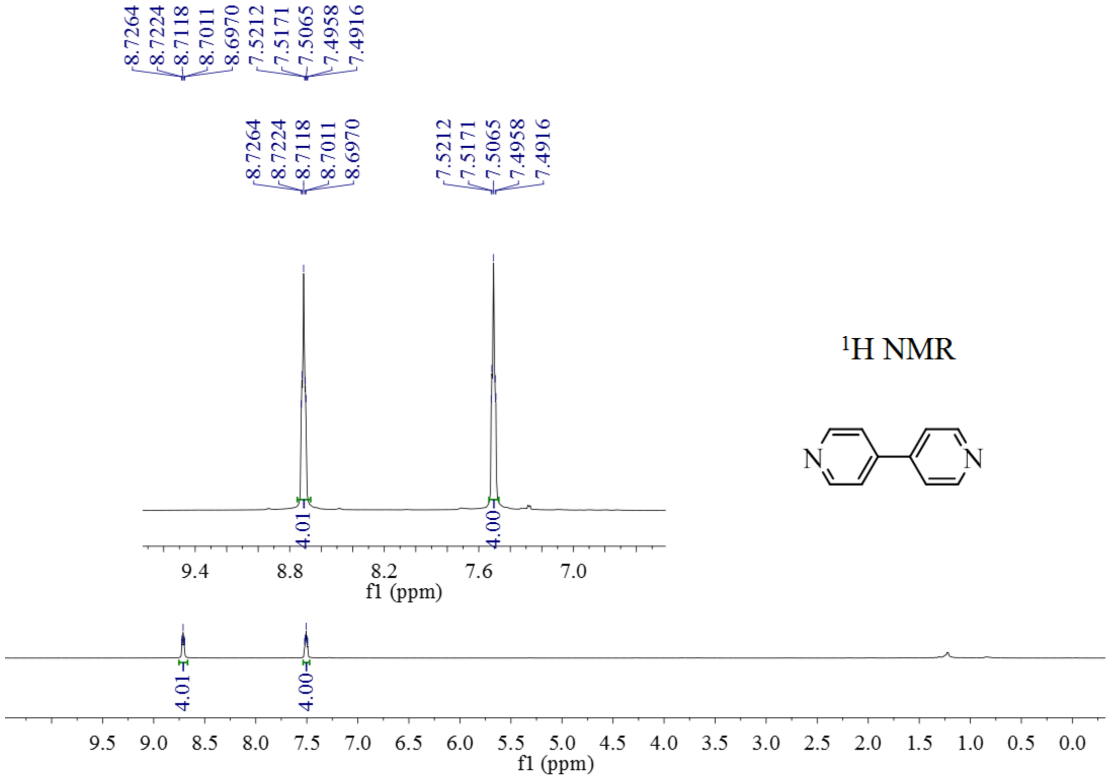

Supplementary Fig. 60: ^{1}H NMR spectrum of 4,4'-bipyridine. Note: this is a known compound.[23] Measurement conditions: 400 MHz, $CDCl_3$, room temperature.

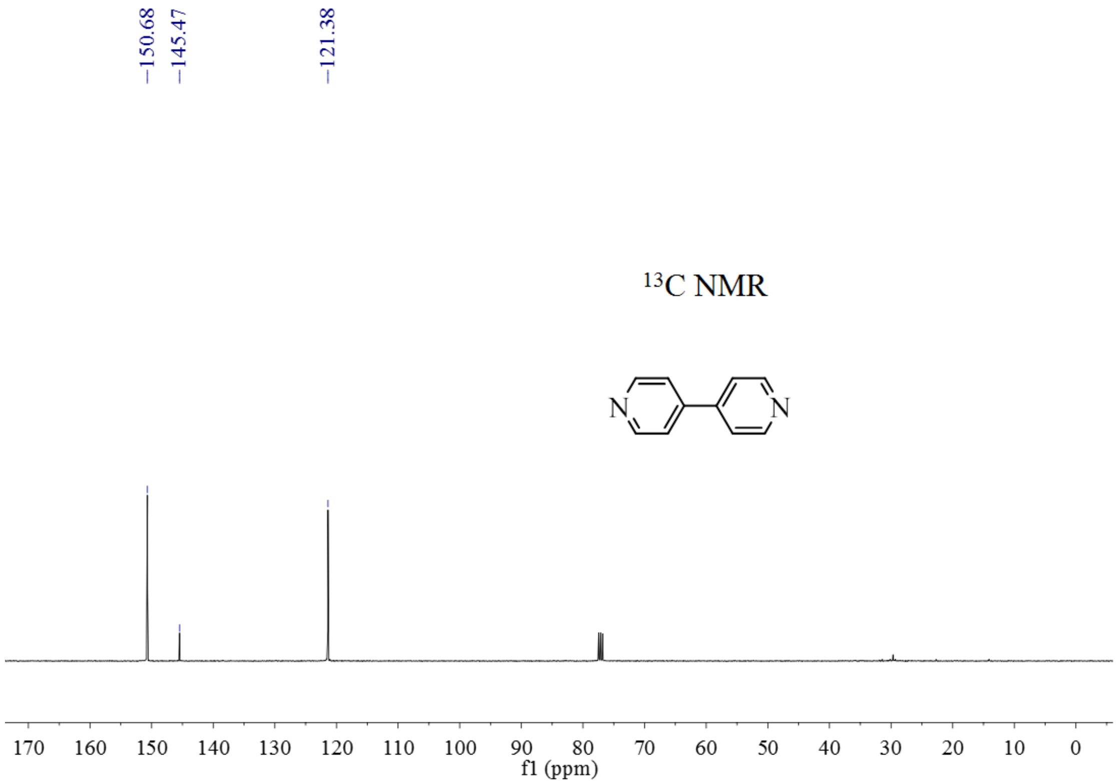

Supplementary Fig. 61: ^{13}C NMR spectrum of 4,4'-bipyridine. Note: this is a known compound.[23] Measurement conditions: 101 MHz, $CDCl_3$, room temperature.

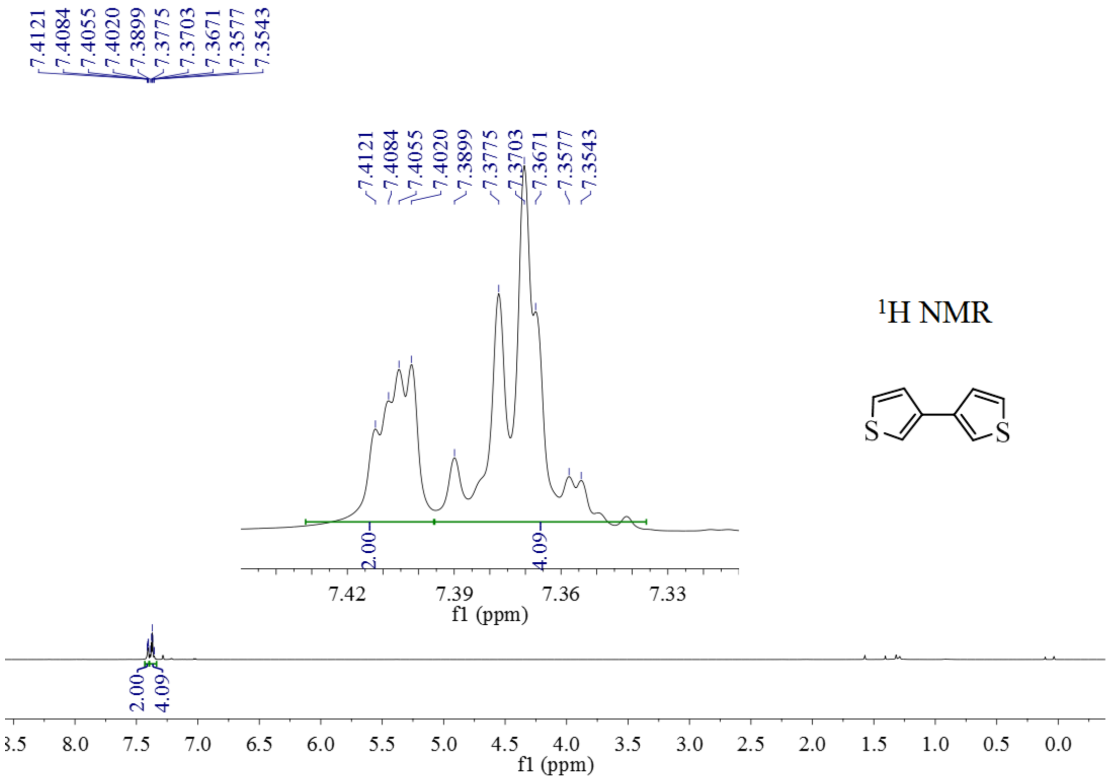

Supplementary Fig. 62: ^{1}H NMR spectrum of 3,3'-bithiophene. Note: this is a known compound.[24] Measurement conditions: 400 MHz, $CDCl_3$, room temperature.

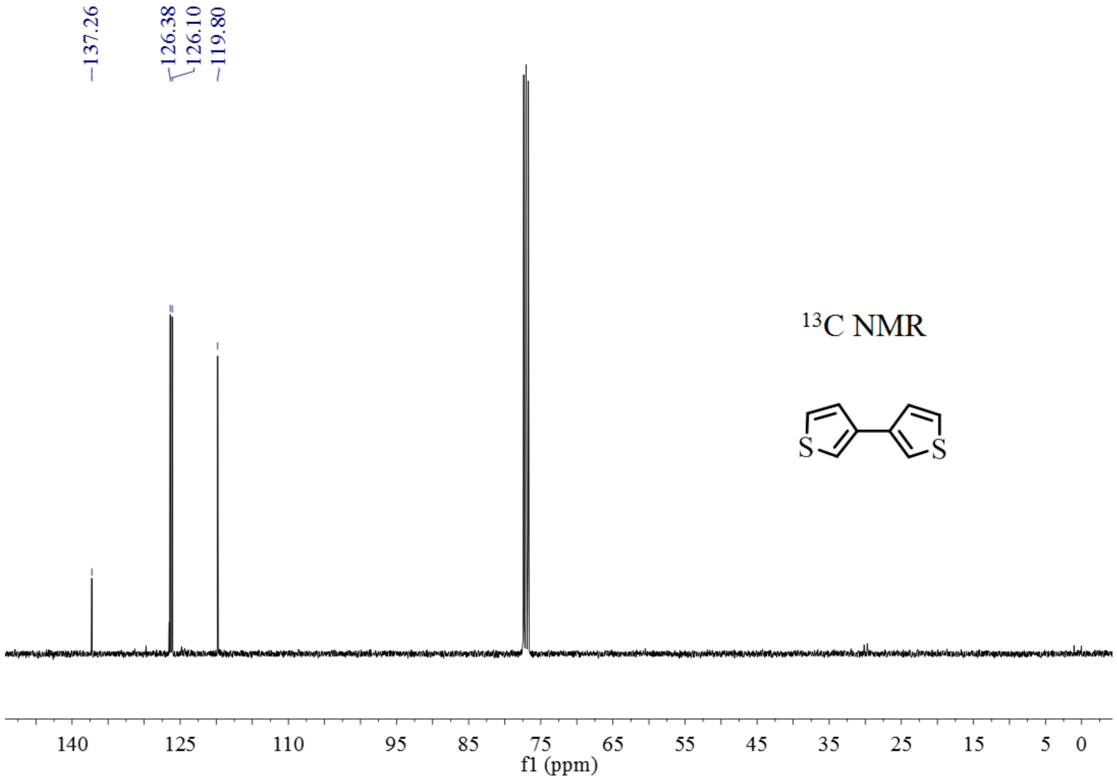

Supplementary Fig. 63: ^{13}C NMR spectrum of 3,3'-bithiophene. Note: this is a known compound.[24] Measurement conditions: 101 MHz, $CDCl_3$, room temperature.

7. Supplementary references

[1] Fan, X., Yao, Y. L., Xu, Y. S., Yu L. & Qiu, C. T. Visible-light-driven photocatalytic hydrogenation of olefins using water as the H source. *ChemCatChem* **11**, 2596–2599 (2019).

[2] Guo, Y. et al. Selectively catalytic hydrogenation of styrene-butadiene rubber over Pd/g-C_3N_4 catalyst. *Appl. Catal. A: Gen.* **589**, 117312 (2020).

[3] Han, C. H. et al. Beyond hydrogen evolution: solar driven water-donating transfer hydrogenation over platinum/carbon nitride. *ACS Catal.* **10**, 9227–9235 (2020).

[4] Rappoport, P., Weiser, B. J. & Kerker, H. *Ultraviolet-visible absorption spectra and photocatalysis of semiconductors.* American Academic Press, New York, 58–63 (2018).

[5] Frisch, M. J. et al. Gaussian 09 Revision D. 01, 2009.

[6] Mennucci, B. & Tomasi, J. Continuum solvation models: A new approach to the problem of solute's charge distribution and cavity boundaries. *J. Chem. Phys.* **106**, 5151–5158 (1997).

[7] Barone, V. & Cossi, M. Quantum calculation of molecular energies and energy gradients in solution by a conductor solvent model. *J. Chem. Phys. A* **102**, 1995–2001(1998).

[8] Lee, C., Yang, W. & Parr, R. G. Development of the colle-salvetti correlation-energy formula into a functional of the electron density. *Phys. Rev. B, Condens. Matter* **37**, 785–789 (1988).

[9] Becke, A. D. Density-functional thermochemistry. III. The role of exact exchange. *J. Chem. Phys.* **98**, 5648–5652 (1993).

[10] Hehre, W. J., Ditchfield, R. & Pople, J. A. Self-consistent molecular orbital methods. XII. Further extensions of Gaussian-type basis sets for use in molecular orbital studies of organic molecules. *J. Chem. Phys.* **56**, 2257–2261 (1972).

[11] Mclean, A. D. & Chandler, G. S. Contracted Gaussian basis sets for molecular calculations. I. Second row atoms, Z=11–18. *J. Chem. Phys.* **72**, 5639–5648 (1980).

[12] Krishnan R, Binkley J S, Seeger R, et al. Self-consistent molecular orbital methods. XX. A basis set for correlated wave functions. *J. Chem. Phys.* **72**, 650–654 (1980).

[13] Liu, Y. J., Zhang, D. M., Xiao, S. H., Qi, Y. & Liu, S. F. Copper-catalyzed homocoupling of alkyl halides in the presence of samarium. *Asian J. Org. Chem.* **8**, 858–862 (2019).

[14] Liu, J. H., Zhang, Y. W., Lu, L. H. Wu, G. & Chen, W. Self-regenerated solar-driven photocatalytic water-splitting by urea derived graphitic carbon nitride with platinum nanoparticles. *Chem. Commun.* **48**, 8826–8828 (2012).

[15] Liu, J. et al. Metal-free efficient photocatalyst for stable visible water splitting via a two-electron pathway. Science 347, 970–974 (2015).

[16] Jia, Q. H. et al. Photocatalytic coupled redox cycle for two organic transformations over Pd/carbon nitride composites. *Catal. Sci. Technol.* **9**, 5077–5089 (2019).

[17] Mo, Z. et al. Constructing Pd/2D-C_3N_4 composites for efficient photocatalytic H_2 evolution through nonplasmon-induced bound electrons. *Appl. Surf. Sci.* **467**, 151–157 (2019).

[18] Fukui, M., Koshida, W., Tanaka, A., Hashimoto, K., Kominami, H. Photocatalytic hydrogenation of nitrobenzenes to anilines over noble metal-free TiO_2 utilizing methylamine

as a hydrogen donor. *Appl. Catal. B: Environ.* **268**, 118446 (2020).

[19] Creighton J. A. & Eadont D. G. Ultraviolet-visible absorption spectra of the colloidal metallic elements. *J. Chem. Soc., Faraday Trans.* **87**, 3881–3891 (1991).

[20] Guo, X.-W. Visible light-driven photocatalytic Heck reaction over carbon nanocoil supported Pd nanoparticles. *Catal. Sci. Technol.* **6**, 7738–7743 (2016).

[21] Bao, F. Y. et al. Palladium/sensory component-catalyzed homocoupling reactions of aryl halides. *Synlett*. **31**, 1501-1506 (2020).

[22] Karimi, B, Behzadnia, H., Vali, H., Palladium on ionic liquid derived nanofibrillated mesoporous carbon: A recyclable catalyst for the Ullmann homocoupling reactions of aryl halides in water. *ChemCatChem* **6**, 745-748 (2014).

[23] Murugan, K., Nainamalai, D., Kanagaraj, P., Nagappan S. G.. Palaniswamy, S. Green-synthesized nickel nanoparticles on reduced graphene oxide as an active and selective catalyst for suzuki and glaser-hay coupling reactions. *Appl. Organomet. Chem.* **34**, e5778 (2020).

[24] Lv, L. Y., Qiu, Z. H., Li, J. B., Liu, M. X. & Li, C. J. N_2H_4 as traceless mediator for homo- and cross- aryl coupling. *Nat. Commun.* **9**, 4739 (2018).

[25] Chauhan, P. et al. Palladium and copper-catalyzed ligand-free coupling of phenylhydrazines in water. *RSC Adv*. **4**, 43336-43340 (2014).

[26] Chen, Z, Y., Chen, G. H., Aboo, A. H., Lggo,J. & Xiao, J. L. Methanol as hydrogen source: transfer hydrogenation of aldehydes near room temperature. *Asian. J. Org. Chem*. **9**, 1174–1178 (2020).

[27] Jiang, H. Y. et al. Efficient photocatalytic chemoselective and stereoselective C-C bond formation over AuPd@N-rich carbon nitride. *Catal. Sci. Technol.* **11**, 219–229 (2021).

[28] Crabbe, B. W., Kuehm, O. P., Bennett, J. C. & Hallett-Tapley, G. L. Light-activated Ullmann homocoupling of aryl halides catalyzed using gold nanoparticle-functionalized potassium niobium oxides. *Catal. Sci. Technol.* **8**, 4907–4915 (2018).

[29] Tran, H., McCallum, T., Morin, M. & Barriault, L. Homocoupling of iodoarenes and bromoalkanes using photoredox gold catalysis: a light enabled Au(III) reductive elimination. *Org. Lett.* **18**, 4308–4311 (2016).

[30] Feizpour, F., Jafarpour, M. & Rezaeifard, A. Band gap modification of TiO_2 nanoparticles by ascorbic acid-stabilized Pd nanoparticles for photocatalytic Suzuki-Miyaura and Ullmann coupling reactions. *Catal. Lett.***149**, 1595−1610 (2019).

[31] Karimi, B., Barzegara, H. & Vali, H. Au-Pd bimetallic nanoparticles supported on a high nitrogen-rich ordered mesoporous carbon as an efficient catalyst for room temperature Ullmann coupling of aryl chlorides in aqueous media. *Chem. Commun.* **54**, 7155−7158 (2018).

[32] Zuo, Z. Q., Kim, R. S. & Watson, D. A. Synthesis of axially chiral 2,2'-bisphosphobiarenes via a nickel-catalyzed asymmetric Ullmann coupling: general access to privileged chiral

ligands without optical resolution. *J. Am. Chem. Soc.* **143**, 1328–1333 (2021).

[33] Prasanna, Bhat, S. K., Usha, K. M. & Hegde, M. S. Ligand and base free synthesis of biaryls from aryl halides in aqueous media with recyclable $Ti_{0.97}Pd_{0.03}O_{1.97}$ catalyst. *Catal. Lett.* **151**, 3313–3322 (2021).

[34] Gong, X. C. et al. Ligand-free palladium catalyzed Ullmann biaryl synthesis: 'household'reagents and mild reaction conditions. *Green Chem.* **21**, 995–999 (2019).

[35] Wang, Z. Y., Peng, X. S. & Wong, H. N. C. Ligand-free iron-catalyzed homo-coupling of aryllithium reagents. *Asian J. Org. Chem.* **9**, 1834–1840 (2020).

[36] Dubey, A. V. & Kumar, A. V. A bio-inspired magnetically recoverable palladium nanocatalyst for the ullmann coupling reaction of aryl halides and arylboronic acids in aqueous media. *Appl. Organomet. Chem.* **34**, e5570 (2020).

[37] Rana, S., Varadwaj, G. B. B. & Jonnalagadda. S. B. Pd nanoparticle supported reduced graphene oxide and its excellent catalytic activity for the Ullmann C-C coupling reaction in a green solvent. *RSC Adv.* **9**, 13332–13335 (2019).

[38] Han, F. Y., Xia, J. W., Zhang, X. L. & Fu, Y. S. PdAu alloy nanoparticles supported on nitrogen-doped carbon black as highly active catalysts for Ullmann coupling and nitrophenol hydrogenation reactions. *RSC Adv.* **9**, 17812–17823 (2019).

[39] Lakshmidevi, J. et al. WEPA: a bio-derived medium for added base, pi-acid and ligand free Ullmann coupling of aryl halides using $Pd(OAc)_2$. *Chem. Commun.* **54**, 12333–12336 (2018).

[40] Schroeter, F., Lerch, S. & Strassner, T. Oxidative and reductive cross-coupling reactions catalyzed by an anionic "ligandless" palladium complex. *Org. Process. Res. Dev.* **22**, 1614–1621 (2018).

[41] Feiz, A., Loni, M., Naderi, S. & Bazgir, A. The β-cyclodextrin decorated with palladium nanoparticles without pretreatment: an efficient heterogeneous catalyst for biaryls synthesis. *Appl. Organomet. Chem.* **32**, e4608 (2018).

[42] Uzelac, M. et al. Tandem Mn-I exchange and homocoupling processes mediated by a synergistically operative lithium manganate. *Angew. Chem. Int. Ed.* **60**, 3247–3253 (2021).

[43] Yan, Z. H. et al. Electrodeposition of (hydro)oxides for an oxygen evolution electrode. *Chem. Sci.* **11**, 10614–10625 (2020).
